# Single-cell transcriptomics and surface epitope detection in human brain epileptic lesions identifies pro-inflammatory signaling

**DOI:** 10.1038/s41593-022-01095-5

**Published:** 2022-06-23

**Authors:** Pavanish Kumar, Amanda Lim, Sharifah Nur Hazirah, Camillus Jian Hui Chua, Adeline Ngoh, Su Li Poh, Tong Hong Yeo, Jocelyn Lim, Simon Ling, Nursyuhadah Binte Sutamam, Enrico Petretto, David Chyi Yeu Low, Li Zeng, Eng-King Tan, Thaschawee Arkachaisri, Joo Guan Yeo, Florent Ginhoux, Derrick Chan, Salvatore Albani

**Affiliations:** 1 Translational Immunology Institute, SingHealth/Duke-NUS Academic Medical Centre, Singapore, Singapore; 2 Paediatrics Academic Clinical Programme, KK Women’s and Children’s Hospital, Singapore, Singapore; 3 Duke-NUS Medical School and Paediatric Neurology Service, KK Women’s and Children’s Hospital, Singapore, Singapore; 4 Duke-NUS Medical School, Program in Cardiovascular and Metabolic Disorders (CVMD) and Centre for Computational Biology (CCB), Singapore, Singapore; 5 Duke-NUS Medical School and Neurosurgical Service, KK Women’s and Children’s Hospital, Singapore, Singapore; 6 Research Department, National Neuroscience Institute, Singapore, Singapore; 7 Department of Neurology, National Neuroscience Institute, Singapore, Singapore; 8 Neuroscience & Behavioral Disorders Program, DUKE-NUS Medical School, Singapore, Singapore; 9 Duke-NUS Medical School and Rheumatology and Immunology Service, KK Women’s and Children’s Hospital, Singapore, Singapore; 10 Singapore Immunology Network (SIgN), Agency for Science, Technology and Research (A*STAR), Singapore, Singapore

**Keywords:** Neuroimmunology, Epilepsy

## Abstract

Epileptogenic triggers are multifactorial and not well understood. Here we aimed to address the hypothesis that inappropriate pro-inflammatory mechanisms contribute to the pathogenesis of refractory epilepsy (non-responsiveness to antiepileptic drugs) in human patients. We used single-cell cellular indexing of transcriptomes and epitopes by sequencing (CITE-seq) to reveal the immunotranscriptome of surgically resected epileptic lesion tissues. Our approach uncovered a pro-inflammatory microenvironment, including extensive activation of microglia and infiltration of other pro-inflammatory immune cells. These findings were supported by ligand–receptor (LR) interactome analysis, which demonstrated potential mechanisms of infiltration and evidence of direct physical interactions between microglia and T cells. Together, these data provide insight into the immune microenvironment in epileptic tissue, which may aid the development of new therapeutics.

## Abstract (web summary)

Single-cell analysis of immune cells from surgically resected human epileptic brain tissues showed heterogeneity and pro-inflammatory signaling in microglia and evidence for direct interaction of microglia with T cells.

## Main

Epilepsy (recurrent unprovoked seizures) is common, with 50 million cases in the world^1,2^, often begins unpredictably and can lead to severe developmental and functional effects. After epilepsy is established, anticonvulsants are the current first-line treatment to attempt to control seizures by reducing neuronal excitability or increasing neuronal inhibition. However, one-third of patients have drug-refractory epilepsy (DRE), in which seizures persist despite the appropriate use of two or more anticonvulsants. DRE treatment is difficult, and currently only resective epilepsy surgery, in which part of the seizure-causing brain tissue is removed, offers a cure. Thus, there is a critical need for a greater understanding of epileptogenesis, the process that generates and perpetuates epilepsy^3^, which may enable the development of more effective and non-invasive therapies.

Neuroinflammation is thought to be a contributing factor to epileptogenesis. Activated glial cells producing inflammatory cytokines within 4 h after seizure induction in animal models and human chronic epileptic tissue have been reported^4–6^. Recent studies have shown brain infiltration of CD4^+^ and CD8^+^ and interleukin (IL)-17-producing γδ T cells from patients with pediatric epilepsy^7^. Our previous study^8^ using mass cytometry also showed increased frequency of IL-17-producing CD4^+^ and CD8^+^ T cells along with reduced numbers of inhibitory LAG3^+^CD8^+^ T cells in peripheral blood from patients with pediatric epilepsy. Case studies have shown effectiveness of anti-inflammatory treatments in epilepsy^9–12^. Thus, an in-depth understanding of immune mechanisms in epilepsy could pave the way for precise and effective immunotherapeutics.

Here, we sought to study immune mechanisms in DRE using single-cell genomics coupled with a systems biology analytical approach. We analyzed lesional brain tissues from patients with DRE using CITE-seq, a multimodal single-cell technology that captures both the transcriptome and protein expression profiles at single-cell resolution^13^. We developed network biology-based analytical approaches to define and interpret the complex relationships between cell types. These LR-based networks suggest enhanced integrin–collagen-mediated interaction as a potential mechanism of lymphocyte infiltration. Such recruitment of immune cells from the periphery creates an immune microenvironment with a striking resemblance to autoimmune diseases of the brain, such as multiple sclerosis (MS).

## Results

### Defining resident and infiltrating immune cells in the epileptic brain

**Fig. 1:**
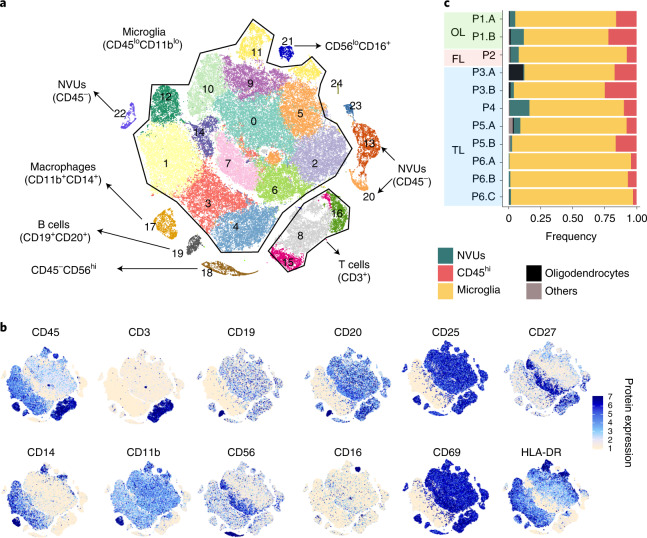
Microglia and infiltrating immune cells in brain tissue from patients with DRE. **a**, Position and phenotype of clusters on the *t*-SNE map. Color represents the cluster ID. **b**, Surface epitope expression of lineage-specific cellular markers quantified using antibody staining with the CITE-seq protocol was overlaid on the *t*-SNE map to identify the cluster phenotype. Color intensity reflects cellular surface epitope protein expression. **c**, Stacked bar chart shows the frequency of infiltrating, resident (microglial) immune cells and NVU cells from brain tissues of patients with DRE. Bar color reflects cell types as indicated in the figure. OL, olfactory lobe; FL, frontal lobe; TL, temporal lobe. P1.A, occipital cortex; P1.B, occipital core; P2, frontal lobe; P3.A, posterior mid-temporal gyrus; P3.B, superior frontal gyrus; P4, posterior mid-temporal gyrus; P5.A, posterior mid-temporal gyrus; P5.B, lateral mid-temporal gyrus; P6.A, mid-temporal gyrus; P6.B, posterior temporal gyrus; P6.C, lateral temporal gyrus.

**Extended Data Fig. 1:**
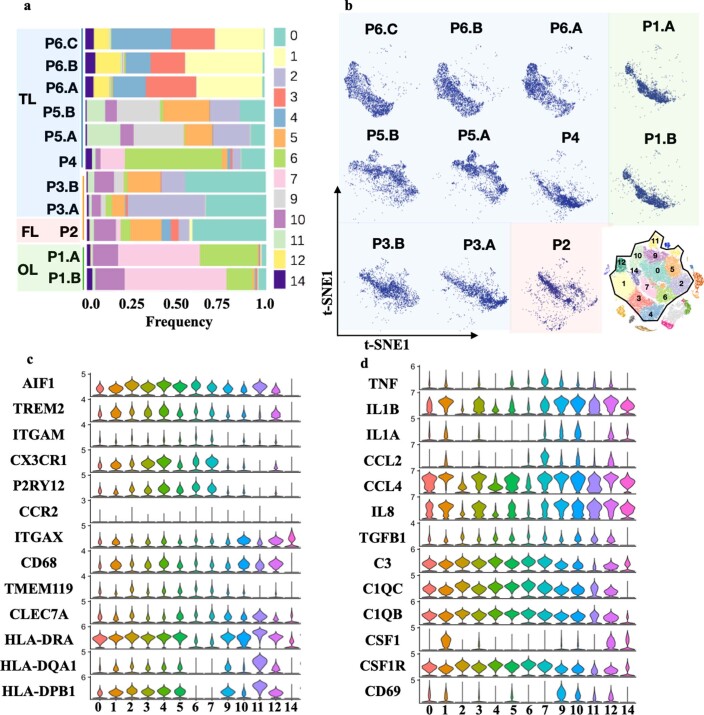
Microglia heterogeneity and differential abundance across the epilepsy patients brain tissue. (**a**) Stacked bar chart shows the frequency of various microglia clusters in each patient’s brain tissue. Color of bar shows the cluster identity. (**b**) Distribution of all the cells from each patient’s brain tissue was plotted as t-SNE map. Stacked bar chart and 2-D t-SNE map both shows differential abundance of microglia clusters across the patient’s brain. (**c**, **d**) To identify the phenotype of microglia clusters, normalized gene expression for each cluster was plotted as violin plot. (c, d) shows the gene expression profile of selected microglia specific, inflammatory and activation marker genes y-axis show normalized expression levels and x-axis shows cluster id.

To identify the immune mechanisms impacting epileptogenesis, single-cell suspensions from brain tissue obtained during resective epileptic brain surgery were subjected to the multimodal single-cell technology CITE-seq. Immune cells were isolated from 11 brain tissue samples from six individual patients and sequenced using the 10x Genomics Single Cell platform. After quality control, a total of 85,780 cells and 22,968 genes were retained for further analysis. The various cell types were clustered, identified and visualized in two-dimensional *t*-distributed stochastic neighbor-embedding (*t*-SNE) maps (Fig. 1a). Clustering and *t*-SNE were performed on gene expression levels using the Seurat R package^14^. Clusters were identified using surface protein markers (Fig. 1b) and gene expression levels (Supplementary Fig. 1 and Extended Data Fig. 1). Surface protein expression overlaid on clusters validates and improves the cluster’s phenotypic identification. CD45 protein surface expression levels discriminate immune cells from non-immune cells. Cell clustering into subsets using graph-based Louvain clustering algorithms resulted in 26 clusters. Based on CD45 expression levels, 13 clusters (0–7, 9–12 and 14) were identified as microglia (CD45^lo^) and six clusters (8, 15–17, 19 and 21) were identified as infiltrating immune cells (CD45^hi^). All other clusters were CD45^−^ and were identified using marker gene expression levels. Clusters 13, 20 and 22 expressed genes (*CLDN5*, *MYH11*, *ABCC9*, *VWF* and *ACTA2*) specific to cells of the neurovascular unit (NVU)^15^, while cluster 18 expressed genes (*MAG* and *MOG*) specific to oligodendrocytes (Supplementary Fig. 1). Among the immune cell subsets, cluster 19 had B cell marker proteins (CD19 and CD20) and cluster 17 had macrophage marker proteins (CD45^hi^, CD14 and CD11b). Clusters 8, 15 and 16 were identified as T cell clusters (CD45^hi^CD3^+^) (Fig. 1a). Infiltrating immune cells were observed in all 11 tissues from the olfactory, frontal or temporal lobes, irrespective of their location in the brain (Fig. 1c). Thus, our approach allowed simultaneous clear identification of microglia, immune cells and non-immune cells of the brain and enabled quantification of lineage-specific surface proteins along with gene expression levels.

### Microglia exhibit a pro-inflammatory phenotype in DRE tissue

**Fig. 2:**
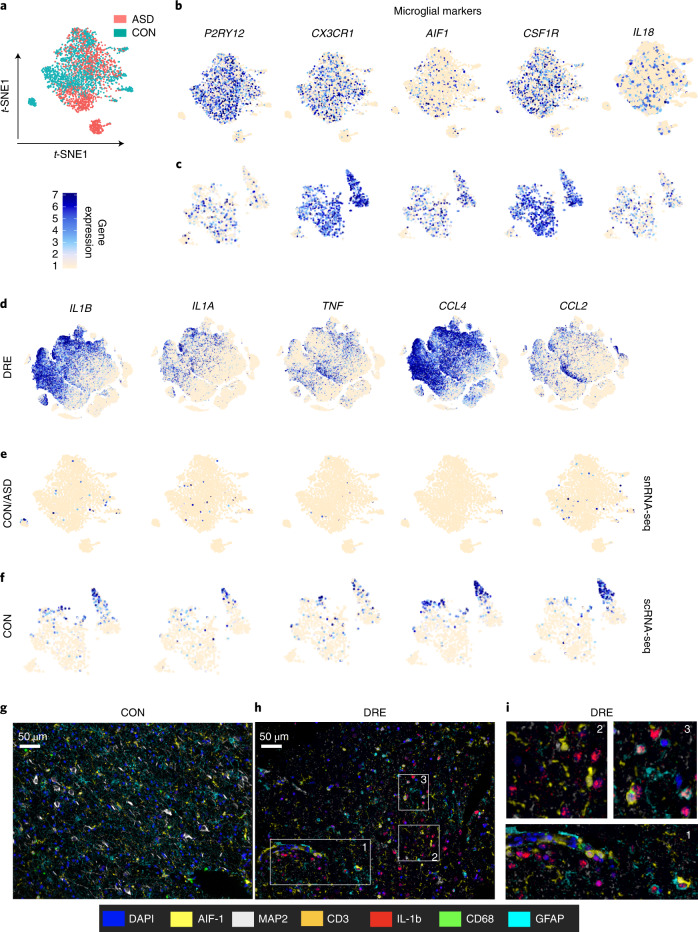
Expression of pro-inflammatory and microglial-specific genes in patients with DRE, non-neurological disease controls and brain tissue of patients with ASD. **a**, Distribution of microglial cells from non-neurological disease controls (CON) and patients with ASD on a t-SNE map. **b**,**c**, snRNA-seq dataset (**b**) and scRNA-seq dataset (**c**) show expression of microglial marker genes overlaid on a t-SNE map. **d**–**f**, Expression of pro-inflammatory cytokines and chemokine marker genes. Normalized gene expression levels were overlaid on the *t*-SNE map. **g**–**i**, Multispectral Opal dye IHC imaging of brain tissue sections from control tissue (**g**) and DRE lesion tissue (**h**). **i**, Magnified image from the DRE tissue section. FFPE (5-µm) tissue sections were stained with panels of antibodies for microglia (AIF-1), macrophages (CD68), T cells (CD3), neurons (microtubule-associated protein 2 (MAP2)), astrocytes (GFAP) and the pro-inflammatory cytokine IL-1b. After staining with all the antibodies, sections were stained with 4,6-diamidino-2-phenylindole (DAPI) for a nuclear stain. Tissues were imaged using the Vectra 3 imaging system with a 40× view finder. White boxes and numbers in **h** correspond to the magnified image in **i**. FFPE brain tissue sections from controls (*n* = 4) and DRE lesions (*n* = 4) were stained and imaged, and a representative image from one sample is shown. The colors that represent the antibody and nuclear stain are shown. Data from Velmeshev et al.^19^ (**a**,**b**,**e**); data from the Masuda et al.^18^ (**c**,**f**).

Microglia, the innate immune cells in the central nervous system (CNS), also play a role in neurogenesis, synapse formation and pruning and in maintaining neuronal homeostasis^16^. Transcriptional heterogeneity in microglia explains their broad functionality^17^. Single-cell genomic studies have revealed diverse subtypes of microglia, which are thought to reflect their distinct functions. Here, we employed single-cell transcriptomics to investigate whether activation of inflammatory pathways could be found in the microglia in DRE tissue. We observed heterogeneous microglial clusters in each patient, indicating the known multiplicity of roles (Extended Data Fig. 1a,b). *AIF1* and *CSF1R* were the most widely expressed genes in all the microglial clusters (Extended Data Fig. 1c). Other microglial-specific genes (*CX3CR1*, *P2RY12* and *TREM2*) showed differential expression across clusters (Extended Data Fig. 1c). Strikingly, the transcriptome of DRE tissue was characterized by a predominance of pro-inflammatory pathways. Indeed, the pro-inflammatory genes *IL1B*, *IL18*, *CXCL8* (IL-8) and *CCL4* were among the most widely expressed chemokine and cytokine genes in DRE microglia. Although epilepsy is not considered a primary immune-mediated disease, DRE microglial clusters 7, 5, 9 and 11 were characterized by high gene expression levels of *TNF*, *HLA-DRA* and *HLA-DPB1* and low *CX3CR1* and *P2RY12* gene expression levels (Extended Data Fig. 1c,d). Complement pathway genes were also highly expressed in all microglial clusters. These findings were homogeneously present in all DRE microglial clusters regardless of differences among individuals or even spatial locations. To compare the findings from DRE microglial single-cell gene expression, data from non-neurological controls and individuals with autism spectrum disorder (ASD) from Velmeshev et al. and Masuda et al. were analyzed^18,19^. Single-nucleus RNA sequencing (snRNA-seq) expression from 3,331 microglial cells (Fig. 2a,b) was obtained from the prefrontal cortex and the anterior cingulate cortex from non-neurological disease controls or human individuals with ASD. Single-cell RNA sequencing (scRNA-seq) expression data were obtained from 1,098 microglia (Fig. 2c) from human brain cortex tissue that was pathologically assessed as normal and resected during epilepsy surgery. Microglia from non-neurological disease controls and individuals with ASD in both the snRNA-seq and scRNA-seq datasets expressed characteristic microglial markers such as *P2RY12*, *CX3CR1*, *AIF1*, *CSF1R* and *IL18* (Fig. 2b,c). Microglia from patients with DRE showed expression of pro-inflammatory cytokine (*IL1B*, *IL1A*, *TNF*, *CCL2* and *CCL4*) and chemokine genes (Fig. 2d). However, microglia from non-neurological disease controls or from individuals with ASD did not show expression of these pro-inflammatory cytokines and chemokines in the snRNA-seq dataset (Fig. 2e). Also, scRNA-seq of brain tissue pathologically assessed as normal showed (Fig. 2f) a much lower proportion of pro-inflammatory cytokine- and/or chemokine-expressing microglial cells (6.9% of cells with *IL1B*-normalized counts >3) than DRE microglia (33.5% of cells with *IL1B*-normalized counts >3). This is in agreement with reported increased IL-1b levels in the cortex of rats with pilocarpine-induced epilepsy compared with control mouse cortex samples^4^. The comparison of microglial gene expression from patients with DRE, non-neurological disease controls and individuals with ASD clearly demonstrates a heightened inflammatory response in DRE microglia (Fig. 2d–f). The widespread expression of pro-inflammatory genes in microglial clusters from patients with DRE has unexpected similarities with microglial expression patterns found in MS, a bona fide autoimmune disease^18^. In particular, clusters 9–12 showed a phenotype similar to that of microglial cell types enriched in MS brain lesions. These clusters show high expression of *HLA-DRA* and *HLA-DPB1* and low expression of *CX3CR1* and *P2RY12* (Extended Data Fig. 1c,d), characteristic of microglia from MS^18^.

**Extended Data Fig. 2:**
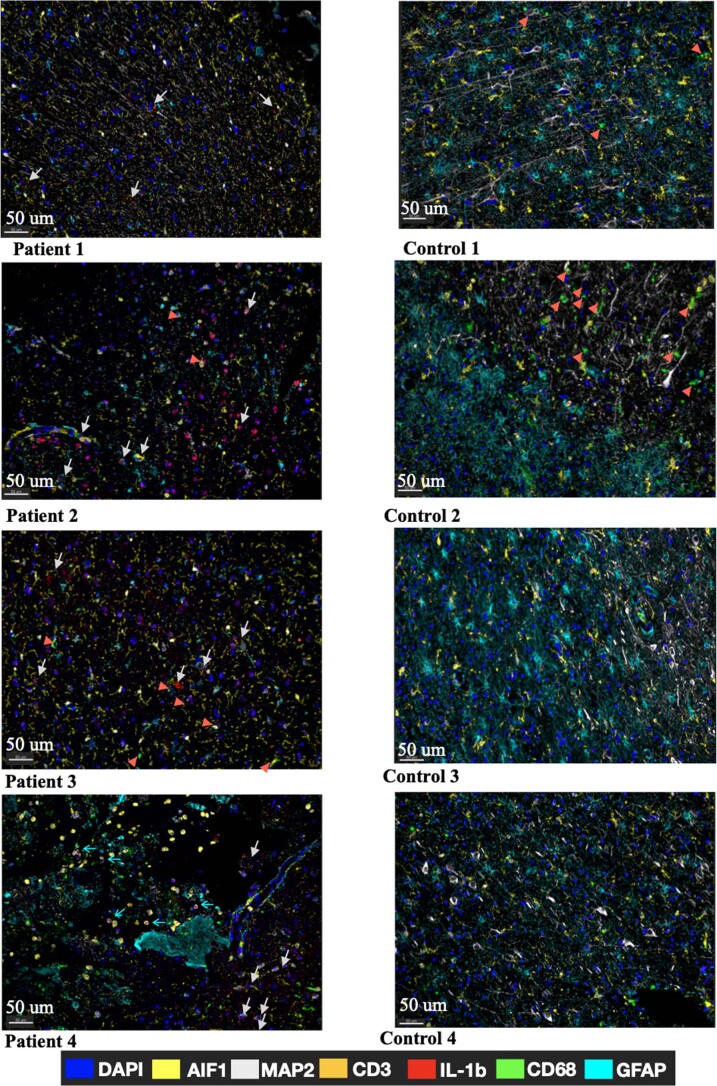
Multispectral 7 color immunohistochemistry (IHC) imaging analysis. Multispectral Opal^TM^ dye immunohistochemistry (IHC) imaging of brain tissue section from (**a**) DRE lesion tissue and (**b**) Control tissue. 5 µm FFPE (Formalin Fixed Paraffin Embedded) tissue sections from 4 DRE patients’ brain and 4 control brain tissue were stained with panels of antibody for microglia (AIF1), macrophage (CD68), T cells (CD3), Neurons (MAP2), Astrocytes (GFAP), Pro-inflammatory cytokine (IL-1b). After staining with all the antibodies sections were stained with DAPI for nuclear stain. Tissues were imaged using Vectra-3.0^TM^ imaging system with 40X view finder. Figure legend shows the colors that represent the antibody and nuclear stain. White arrow shows IL-1b staining, red arrowhead shows CD68 stain and thin cyan arrow shows CD3 stain.

To validate the transcriptomic findings, we sought to image the brain microenvironment using multispectral Opal dye 7 color immunohistochemistry (IHC) imaging analysis. This demonstrated pro-inflammatory cytokine IL-1b production by microglia from DRE lesions (Fig. 2g–i and Extended Data Fig. 2). Allograft inflammatory factor 1 (AIF1)-stained microglial cells in DRE brain lesions produced IL-1b (Fig. 2h), while IL-1b was not observed in control brain tissue sections (Fig. 2g). Aside from microglia, astrocytes stained for glial fibrillary acidic protein (GFAP) also produced IL-1b in DRE tissue sections (Extended Data Fig. 2). Our IHC imaging analysis also corroborates the findings in rat and human epilepsy of Ravizza et al.^4^.

**Extended Data Fig. 3:**
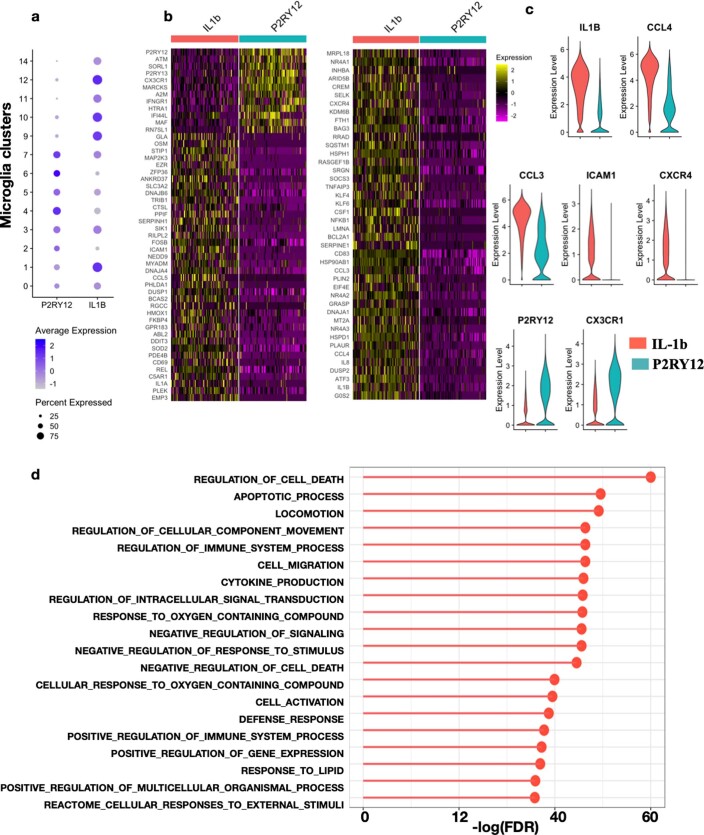
IL-1b expressing microglia genes expression profile compared with P2RY12 expressing microglia. (**a**) Dotplot shows the normalized expression P2RY12 and IL1B in microglia clusters from DRE patient’s brain. Dot size reflects the percentage of cells that showed genes while color shows the expression levels of genes. (**b**) Heatmap of genes significantly modulated in IL-1B expressing clusters compared to P2RY12 expressing clusters. For heat map 100 random sampled cells were shown for both IL1b and P2RY12 cluster. **c**) IL1B, P2RY12 clusters marker, Chemokine CCL4, CCL3 cell adhesion genes ICAM1 and chemokine receptors CXCR4 and CX3CR1 were shown as violin plot. (**d**) GO gene set enrichment analysis results was shown as lollipop plot where on x-axis -log of FDR adjusted p-value for GO terms were shown. Top 20 GO term were shown in the figure.

Purinergic receptor P2RY12 on the microglial surface responds to neuronal activation by sensing ATP released by activated neurons and activated astrocytes^20^. Our data showed reduced expression of the *P2RY12* gene in clusters that had higher *IL1B* expression levels (Extended Data Fig. 3a). We further analyzed the genes differentially regulated in *IL1B*-expressing clusters (1, 9, 10 and 12) compared with *P2RY12*-expressing clusters (4 and 6). Differential gene expression analysis showed that 81 genes were significantly upregulated (adjusted *P* value < 0.05, log_2_ (fold change) > 1) and 12 genes were significantly downregulated in *IL1B*-expressing cells compared with *P2RY12*-expressing cells (Extended Data Fig. 3b and Supplementary Table 1). *IL1B*-expressing cluster cells notably showed higher expression of pro-inflammatory cytokines, chemokines and adhesion molecules (Extended Data Fig. 3c). *IL1B*-expressing cluster cells also showed lower *CX3CR1* expression than *P2RY12*-expressing cluster cells. Furthermore, gene ontology term enrichment analysis showed genes enriched for apoptosis, locomotion, cell migration, the immune system, cytokine production and negative regulation of cell death (Extended Data Fig. 3d). Differential gene expression analysis clearly indicated changes in the functional and structural aspects of *P2RY12*-expressing microglia compared with *IL1B*-expressing pro-inflammatory microglia. Altogether, these findings strongly suggest an underlying primary immune imbalance contributed to by resident microglia, which generates a microenvironment conducive to chronic immune inflammation. These observations, along with previous studies^4,21^, strongly enforce the hypothesis that epileptic foci resected in patients with DRE possess an immune pathogenic environment, capable of interfacing with the immune system to attract and elicit innate and adaptive immune cells. Thus, we set out to characterize the immune cells infiltrating the DRE focus.

### Infiltration of leukocytes in the brains of patients with DRE

**Fig. 3:**
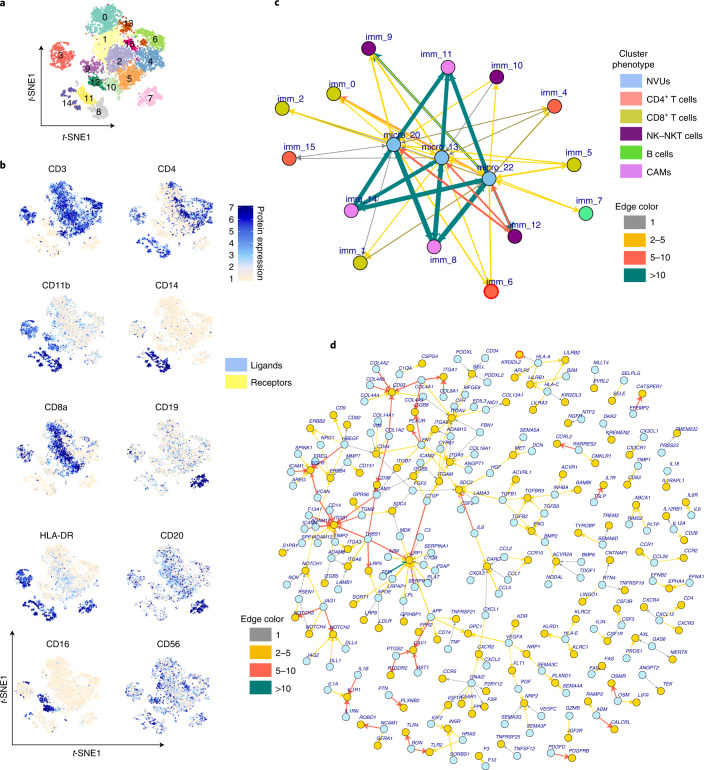
Infiltrating immune cells in the epileptic human brain and their interaction with NVU cells. **a**, Cluster positions are shown on a t-SNE map where color represents cluster identity. **b**, Surface epitope expression of lineage-specific cellular markers quantified using antibody staining with the CITE-seq protocol was overlaid on a t-SNE map to identify the cluster phenotypes. Color intensity reflects the expression of cellular surface proteins. **c**, LR interaction network between NVUs and immune cell clusters is shown as a directed network graph. Network nodes reflect cluster ID, and edges shown as colored arrows reflect the potential interaction between cognate LR pairs. Arrow direction shows signaling from ligands (arrow tail) to receptors (arrowhead). Thickness and color of the arrows reflect the number of LR pairs found between the two nodes. Node ID is shown as imm_ (for immune cell clusters) and micro_20, micro_13,and micro_22 for NVU clusters. Node colors show the cell type. **d**, Network of all ligand and receptor gene pairs found enriched between clusters of NVUs and immune cells. Here nodes represent ligands (blue circles) and receptors (yellow circles), and edges show cognate ligand and receptor interactions. Recently updated gene names include *CYR61 (CCN1), CTGF (CCN2), NOV (CCN3), MLLT4 (AFDN), PVRL2 (NECTIN2)*. Arrow directions show signaling from ligands (arrow tail) to receptors (arrowhead). Arrow colors show the number of cluster pairs for which an LR pair was enriched.

**Extended Data Fig. 4:**
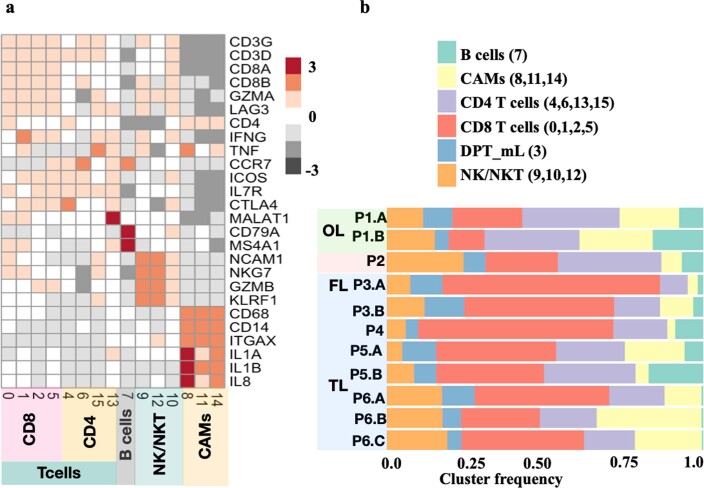
Infiltrating immune cell clusters phenotype and abundance across patients and brain tissues. (**a**) Heatmap shows the expression of cell type specific genes. Heatmap was plotted with normalized mean expression levels of genes in the clusters. Black color shows the low expression while red show higher levels. (**b**) Distribution of infiltrating immune cells shown as stacked bar chart. Clusters were merged into major cell type as shown in figure legend. Figure legends color shows major cell types and numbers in brackets shows clusters id that were grouped together to plot stacked bar chart.

Inspired by the initial observation of Xu et al.^7^, who described immune infiltrates in the brain parenchyma of a patient with pediatric epilepsy by using flow cytometry, we analyzed the data to show in-depth mechanistic characteristics of infiltrating immune cells using single-cell transcriptomic analysis. Immune cell clusters (8, 15–17 and 19) were identified using major lineage surface protein markers and gene expression levels (Fig. 1b). These immune cell clusters were reclustered to further resolve the major immune cell lineage into its functional subsets. Louvain clustering grouped immune cells into 16 clusters. The phenotypes and distributions of these clusters were visualized with a t-SNE map (Fig. 3a). Surface protein expression levels were overlaid on *t*-SNE maps to identify and visualize the cluster phenotype (Fig. 3b). Cluster phenotypes were further validated at the gene expression level (Extended Data Fig. 4a). We further validated RNA assay-based clusters with the antibody-derived tags (ADT) assay (protein expression) or integrated joint ADT and RNA assay clusters. The similarity network fusion (SNF) algorithm implemented in the CiteFuse R package^22^ was used for integrated data-clustering analysis. Spectral clustering was performed, and the optimal cluster number was obtained using eigen values as described in the CiteFuse package. Cluster information was overlaid on a t-SNE map (Supplementary Fig. 2).

**Extended Data Fig. 5:**
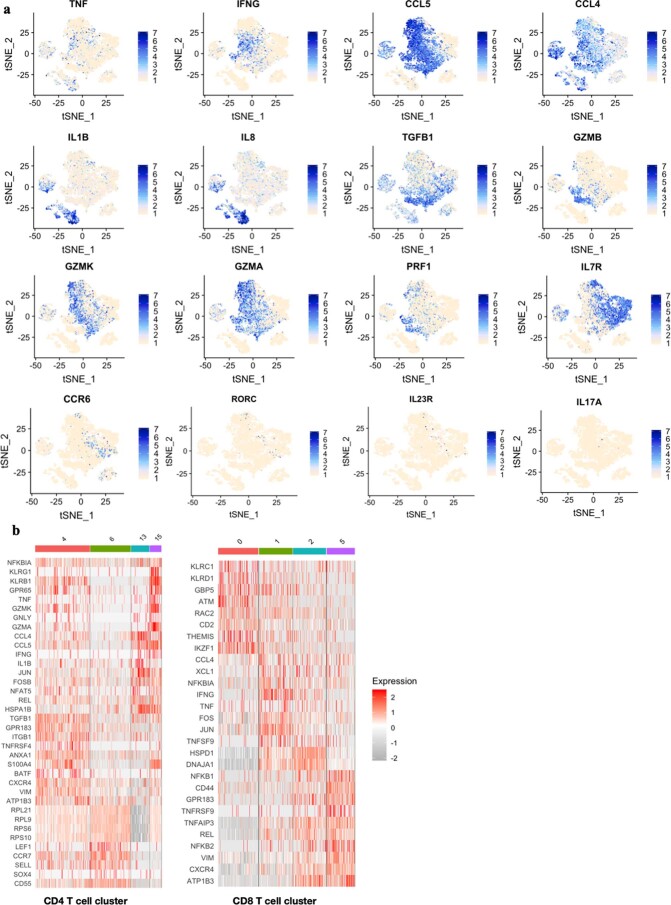
Pro-inflammatory cytokines and chemokine production by infiltrating immune cells. (**a**) Gene expression overlaid on t-SNE map to shows the expression levels of pro inflammatory, cytolytic genes in brain infiltrating immune cells. (**b**) Heatmap of genes expressed in CD4+ and CD8+ T cell clusters. Each column of heat map shows one single cell. Log normalized values are used for heat map and t-SNE overlay plots.

Spectral clustering based on ADT (Supplementary Fig. 2a) was able to capture the major lineages CD8^+^ T (cluster 2), CD4^+^ T (cluster 1) and B (clusters 5 and 3) cells, while CNS-associated macrophages (CAMs) and natural killer (NK) and NKT cells were mixed (clusters 4 and 6). SNF-enabled clustering improved the ADT-based clusters (Supplementary Fig. 2b) and was able to separate NK–NKT cells (cluster 3) from CAMs (cluster 2), and B cells were merged in one cluster (cluster 5). Major cell type clusters from SNF-based clustering were in concordance with RNA-based clustering. Altogether, RNA-based clustering (Supplementary Fig. 2c and Fig. 3a,b) could provide a higher level of granularity. Considering the similarity and concordance of SNF clustering with RNA clustering, we chose fine-resolved RNA-based clusters in further analyses. We found cell subsets of CD4^+^ and CD8^+^ T cells, B cells, macrophages, dendritic cells (DCs) and NK–NKT cells. Clusters 9 and 12 were CD3^−^CD16^+^ at the surface protein level, while cluster 10 was CD3^+^CD16^−^, discriminating NK cells (clusters 9 and 12) from NKT cells (cluster 10) (Fig. 3b). NK cells had a CD56^dim^CD16^+^ cytolytic NK cell subset phenotype. NK cells were *GZMK* (encoding granzyme K) negative but expressed *GZMB* (encoding granzyme B) (Extended Data Fig. 5a). By comparison, patients with MS who have responded to immunotherapy show an increase in CD56^bright^GZMK^+^ regulatory NK cells^23^. Hence, infiltration of mature cytotoxic NK cells in DRE may further contribute to neurodegeneration. Cluster 3 showed a mixed phenotype with surface protein expression of CD3, CD4, CD8, CD11b and HLA-DR, indicating doublets and as such possibly interacting cells. Cluster 3 was further analyzed and resolved (Direct interaction between microglia and T cells). All major immune cell subsets were found in the brain sections analyzed (Extended Data Fig. 4b). Infiltrating CD4^+^ and CD8^+^ T cells were the most abundant (Extended Data Fig. 4b). Intriguingly, DRE-M focus-infiltrating T cells clearly showed a pro-inflammatory function and expressed *IFNG*, *TNF*, *CCL5* and *CCL4* (Extended Data Fig. 5a). The cluster 6 CD4^+^ T cell subset expressed genes (*CCR7*, *LEF1*, *SOX4*, *RPL9* and *RPS6*) characteristic of naive and actively proliferating CD4^+^ T cells. Three other CD4^+^ T cell subsets (clusters 4, 13 and 15) showed an activated T cell phenotype and expressed higher levels of chemokines and cytokines (*CCL4*, *CCL5*, *TNF*, *IL1B* and *IFNG*) as well as adhesion and locomotion (*CXCR4*, *VIM*, *ATP1B3* and *ANXA1*)-associated genes (Extended Data Fig. 5b). Within the CD4^+^ T cell compartment, we observed cells with a helper T cell (T_H_)17 gene signature (*CCR6*, *RORC* and *IL23R*) (Extended Data Fig. 5a). Similar to CD4^+^ T cell subsets, CD8^+^ T cell subsets also showed an activated cell phenotype. CD8^+^ T cell subsets expressed higher levels of *GZMK* (encoding granzyme K) and *GZMA* (encoding granzyme A), indicating their effector cytotoxic functionality. In addition to pro-inflammatory and activated subsets, we observed immature and activated CD8^+^ T cell subsets (cluster 0). This immature subset expressed *CD2*, *THEMIS*, *IKZF1*, *KLRC1*, *ATM*, *GBP5* and *RAC2*. Both CD4^+^ and CD8^+^ T cell subsets expressed genes (*NFKBIA*, *REL*, *NFKB1*, *TNFRSF9*, *TNFRSF4*, *JUN* and *FOS*) associated with nuclear factor (NF)-κB-mediated signaling (Extended Data Fig. 5b). Despite the heterogeneity observed, most T cell subsets expressed pro-inflammatory genes and showed an activated phenotype along with enhanced trafficking and locomotion gene expression. CAM clusters (clusters 8, 11 and 14), similar to microglia, expressed *IL1B*, *IL8* and *CCL4* pro-inflammatory genes.

Using IHC imaging analysis, we observed infiltrating CD3^+^ T cells from formalin-fixed paraffin-embedded (FFPE) brain sections from patients with DRE and no infiltrating CD3^+^ T cells from control brain sections (Extended Data Fig. 2). Conventionally, FFPE sections and immunofluorescence and/or IHC staining is used to demonstrate infiltration of lymphocytes and other characteristics. However, analysis is often limited to an exceedingly small slice of brain (often thin brain tissue slides of 5–7 µM) and is further limited by availability of IHC-compatible antibodies. Analysis restricted to such a small tissue section could be a possible reason for identifying few infiltrating immune cells in our IHC analysis. The CITE-seq analysis approach precisely overcomes the limitations of IHC and FFPE tissue analysis. Together, most infiltrating immune cells also displayed pro-inflammatory and cytotoxic function, further corroborating inflammation as an underlying pathogenic mechanism. The functional interface between the resident pro-inflammatory microenvironment and the infiltrating immune cells was our next area of investigation.

### Functional LR interactions between NVUs and immune cells

**Extended Data Fig. 6:**
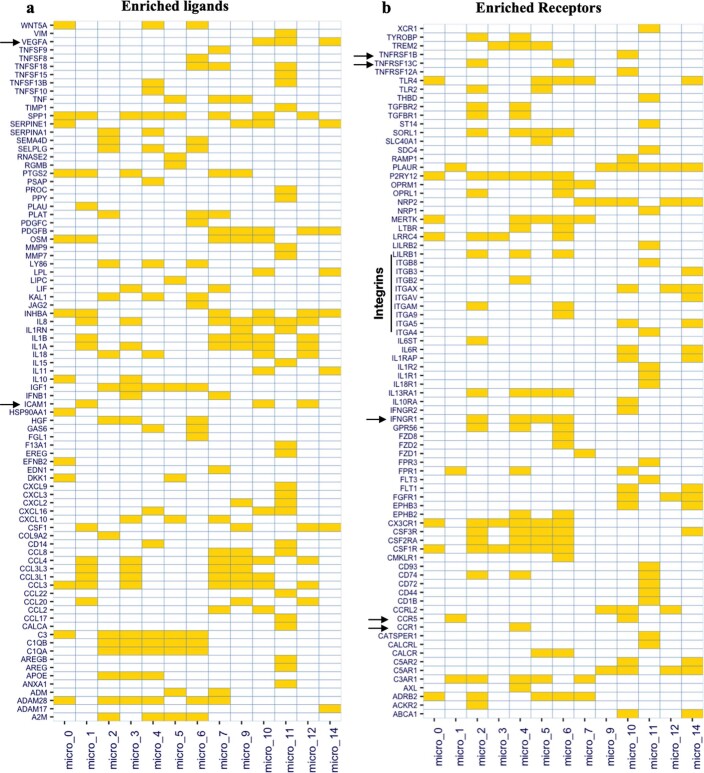
Enriched Ligands and Receptor in microglia clusters. Enriched Ligands (**a**) and Enriched receptors (**b**) in microglia clusters were shown as tilemap, where filled rectangle (yellow color) shows enrichment of ligand/receptor in clusters indicated on x-axis. Expression of ligand/receptor in a cluster compared to all other clusters with log2fold > 1 was considered enriched.

**Extended Data Fig. 7:**
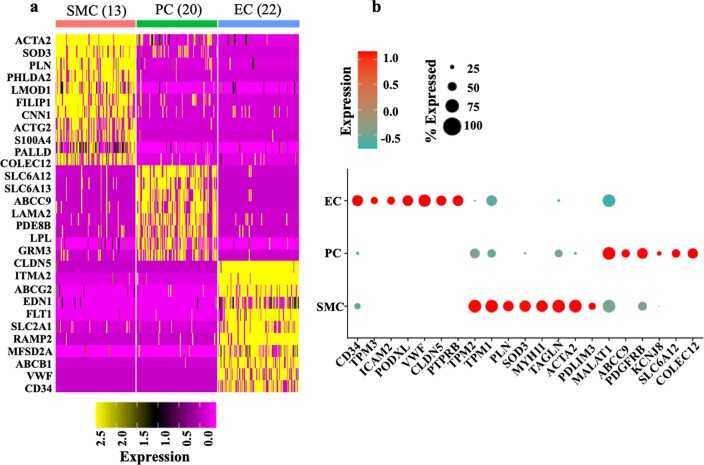
Neurovascular unit (NVU) clusters phenotype. (**a**) Top10 genes specifically expressed genes in NVU clusters (cluster 13, cluster 20 cluster 23) were displayed as Heatmap. Rows of Heatmap shows genes expression and columns shows randomly sampled 100 cells from each cluster. Top of the Heatmap colorbar shows cell type identity (red- smooth muscle cell (SMC), green- pericytes (PC), Blue- Endothelial cells (EC)) and in brackets cluster id was shown. Top 10 genes were found using FindMarker function of Seruat R package. (**b**) Dotplot of key genes specific to endothelial cells (EC- MYH11+ACTA2+), pericytes (KCNJ8+ABCC9+) and smooth muscle cells (CLDN5+VWF+) along with some other known specific markers were shown as dot plot. Color of dots represent expression of genes while size of the dot shows percentage of cells genes expressed. Heatmap and Dotplot are plotted using Seurat R package.

**Extended Data Fig. 8:**
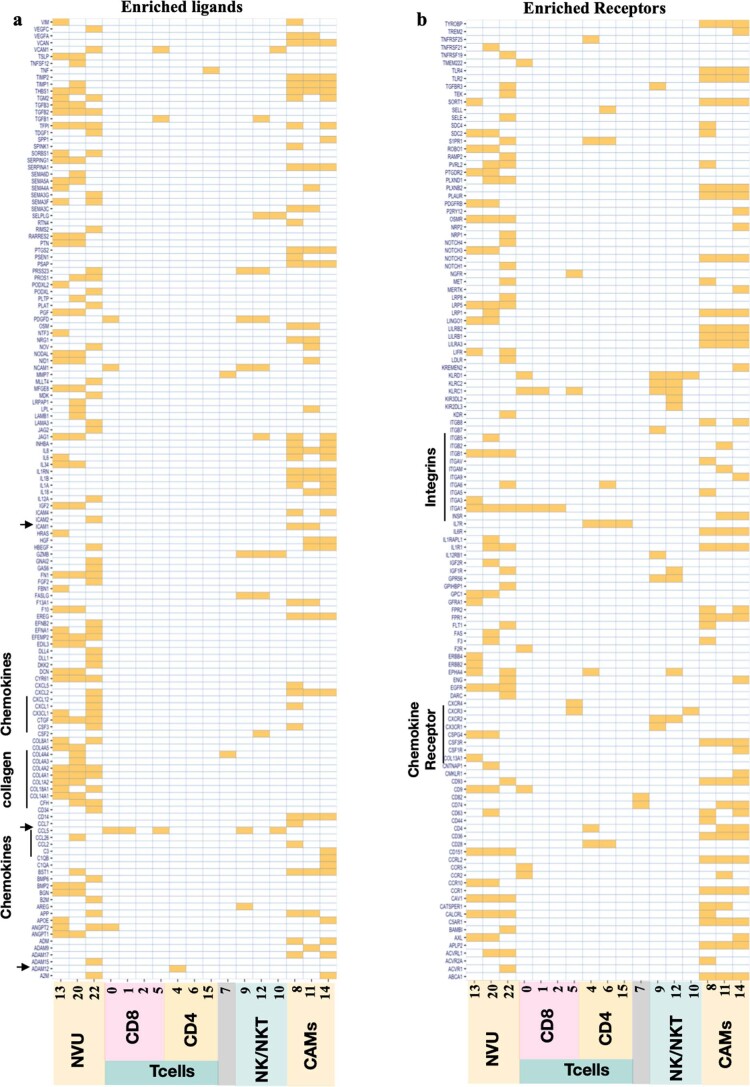
Enriched Ligands and Receptor in NVU and Immune cell clusters. Enriched Ligand (**a**) and Enriched Receptors (**b**) in immune cells and NVU cells clusters were shown as tile Heatmap, where filled rectangle (yellow color) shows enrichment of ligand in clusters indicated on x-axis. Expression of ligand/receptor in a cluster compared to all other clusters with log2fold > 1 was considered enriched.

We hypothesized that the pro-inflammatory DRE-M interfaces with circulating immune cells and induces their extravasation by modulating chemokine receptors and adhesion molecules, which would induce higher and coordinated expression of integrins, selectins and chemokine and cytokine receptors by infiltrating immune cells. These interactions facilitate adhesion, rolling and diapedesis of immune cells from the vasculature into the brain tissue^24–26^. Indeed, within the DRE-M, typical adhesion genes *ITGA (ITGA1, ITGA5, ITGA9)*, *ITGB (ITGB2, ITGB7, ITGB8)*, *VEGFA* and *ICAM1* and chemokine and cytokine receptor genes *CCR5*, *CCR1*, *IFNGR1*, *TNFRSF13C* and *TNFRSF1B* had enriched expression in microglial clusters (Extended Data Fig. 6). We also analyzed NVU (clusters 13, 20 and 22) cells from brain tissue. Based on known specific marker genes^14^, cluster 13 (*ACTA2*, *PLN* and *MYH11*) was identified as smooth muscle cells (SMCs), cluster 20 (*ABCC9* and *LPL*) was identified as pericytes and cluster 22 *(VWF* and *CLDN5*) was identified as endothelial cells (Extended Data Fig. 7). NVU cells also showed enriched expression of *ITGA*, *ITGB*, *VCAM1*, genes encoding collagens and *CCR10* among the prominent adhesion and chemokine receptor genes (Extended Data Fig. 8). Altogether, the data above clearly depict a strong fertile environment generated by the DRE-M for chemokine- and cytokine-mediated immune cell infiltration. This potential LR chemoattractive DRE-M was functionally effective. Indeed, the infiltrating immune cells expressed various chemokine- and cytokine-mediated trafficking (*CXCR4*, *CXCR3*, *CXCR6*, *CCR6*, *CCR4*, *CXCR5* and *CCR7*) and integrin receptor (*ITGB1*, *ITGB2*, *ITGA1* and *ITGA5*) genes (Extended Data Fig. 8) that facilitate immune surveillance and infiltration into brain tissue.

**Fig. 4:**
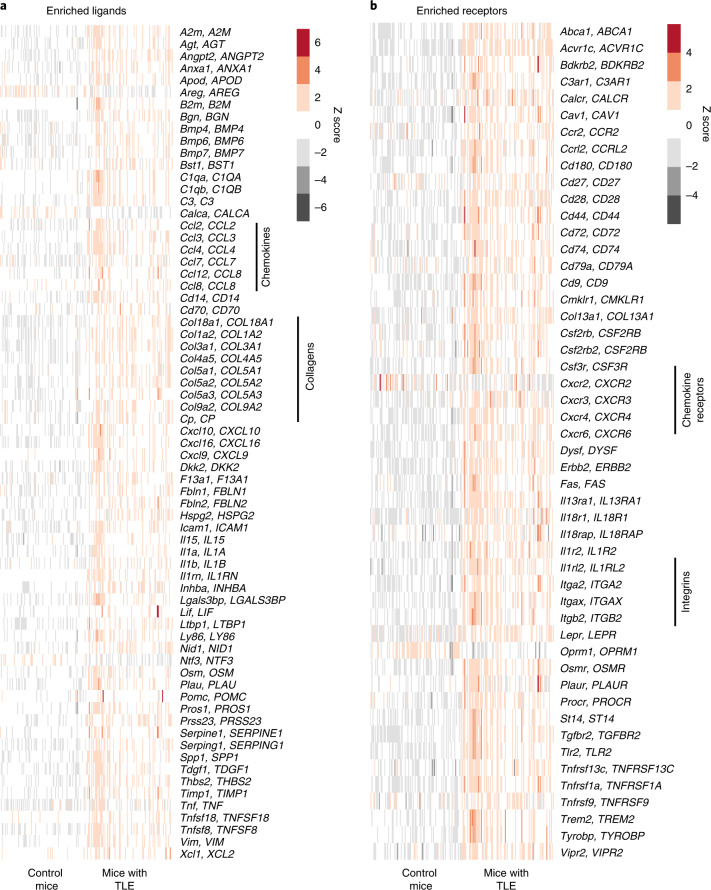
LR genes significantly modulated in a TLE epilepsy mouse model. **a**,**b**, LR network genes enriched in human epileptic brain foci (Fig. 3d and Extended Data Fig. 9) were investigated for differential gene regulation in a TLE mouse model compared with control mice. RNA-seq data from hippocampal brain tissue from mice with TLE (*n* = 100) and control mice (*n* = 100) were analyzed. Expression of significantly differentially regulated (exact test and FDR < 0.05) ligand (**a**) and receptor (**b**) genes is shown as a heatmap. Each row of the heatmap shows a mouse gene and its human ortholog gene (shown in uppercase letters), and each column represents data from an individual mouse.

To study the interactions between NVUs from the DRE-M and infiltrating leukocytes, we created a cellular network based on the LR interaction^27^ between the cell clusters within NVUs and infiltrating immune cells (Fig. 3c). Enriched ligand and receptor genes from each cluster were obtained, and the interaction network was created as described in the Methods. LR pairs between three NVU and 15 immune cell clusters were obtained to create the interaction network between neurovascular and immune cell subsets. If an LR pair was found between the clusters, an edge was established between the clusters. The cluster ID represents nodes, and the interacting LR pair represents the edge between the nodes. Of a maximum possible 90 edges between three NVU clusters and 15 immune cell clusters, we observed 68 edges. We counted the number of LR pairs between the clusters to represent the strength of interaction between the cells and considered a higher number of LR pairs between two cell clusters to indicate a stronger possibility of interaction between them. We found a total of 809 LR interactions among nodes, with 265 unique LR pairs. The majority of LR pairs (206 of 265) were found between NVU and CAM clusters, and these 206 unique LR pairs between CAMs and NVUs accounted for 657 of 809 total LR interactions and are reflected in the network (Fig. 3c) as thick green arrows. NK cell clusters (9 and 12) had higher numbers of interacting LR pairs with NVUs than T and B cell clusters. These results indicate increased infiltration potential of innate cells and high interaction potential with DRE-M. The greater LR-mediated interaction between macrophages and NVUs corroborates and explains previous reports of substantial macrophage infiltration in brain tissue from rodent and human epilepsy^4,6^. We further explored such relational mechanisms and analyzed LR pair genes. Accordingly, the LR gene network was created from 265 unique LR pairs observed between the NVU and immune cell clusters (Fig. 3d). Ligand and receptor genes are shown as nodes, and edges between the nodes show the interaction between nodes. Edge color shows the count of the LR pair genes found between any two inter-NVU and immune cell clusters. The LR gene network showed many collagen–integrin-mediated interactions as indicated by red edges (Fig. 3d). Many of the collagen proteins were expressed by NVUs (Extended Data Fig. 8a) that interact with integrins on immune cells (Fig. 3d and Extended Data Fig. 8b). Among the LR pairs, *ITGB1* was enriched in NVUs, while its cognate ligands (encoded by *CD14*, *ICAM4* and *ADAM17*) were expressed on CAMs (Extended Data Fig. 8a). *ITGA1* was enriched in NVUs but also in CD8^+^ clusters. The *ITGA1* cognate ligand *COL8A1* was expressed in SMCs and endothelial cells. All three CD4^+^ nodes had enriched *IL7R* expression (Extended Data Fig. 8b), and *TSLP*, encoding its potential cognate receptor (IL-7-like cytokine) was enriched in NVUs, particularly in SMCs and pericytes (Extended Data Fig. 8a). Many chemokines and cytokines expressed by immune cell clusters had cognate receptors enriched in NVUs (Fig. 3d and Extended Data Fig. 8). Interestingly, *TNF*, *CCL5* and *TGFB1* were expressed by immune cell clusters (Extended Data Fig. 8a), while their cognate receptors *TNFRSF21*, *ACKR1* and *ACRL1* were expressed by NVUs (Extended Data Fig. 8b). We further validated our interactome results using a recently published method in the CellChat R package^28^ (Supplementary Fig. 3). We extended our interactome analysis in a pilocarpine-induced mouse model of temporal lobe epilepsy (TLE). We analyzed the ligand and receptor genes that we found enriched in our DRE dataset (Fig. 3d) and compared them to the mouse TLE model and control mouse hippocampal brain tissue RNA-seq data generated by Srivastava et al.^29^ to determine whether the observed perturbations in ligand and receptor genes from our human DRE dataset were also present in the mouse TLE model. RNA-seq data were preprocessed and analyzed as described in the Methods. We found 1,600 genes (Supplementary Table 2) that were significantly modulated (false discovery rate (FDR) < 0.05 and fold change >2 or <0.5) in TLE mice (*n* = 100) brain tissue compared with that of control mice (*n* = 100). Among differentially modulated genes, 122 were LR genes that were enriched in human DRE (Fig. 4a,b). Among these LR genes, many key LR pairs, such as collagen (*COL1A2*, *COL3A1*, *COL4A5* and *COL5A3*)–integrin (*ITGA2*, *ITGB2* and *ITGAX*) and chemokine and cytokine (*CCL4*, *CCL2*, *CXCL10*, *IL1B*, *IL1A* and *TNF*)–chemokine and cytokine receptor (*CXCR4*, *CXCR6*, *IL1R2* and *TNFRSF9*) genes, were upregulated in the TLE mouse brain compared to in the control mouse brain. The data from the experimental animal epilepsy model further corroborate our findings in humans. Altogether, our LR interactome network defines the mechanism of immune cell infiltration pivoting on the DRE-M and capable of attracting immune cells with a clear pro-inflammatory bias.

### Direct interaction between microglia and T cells

**Fig. 5:**
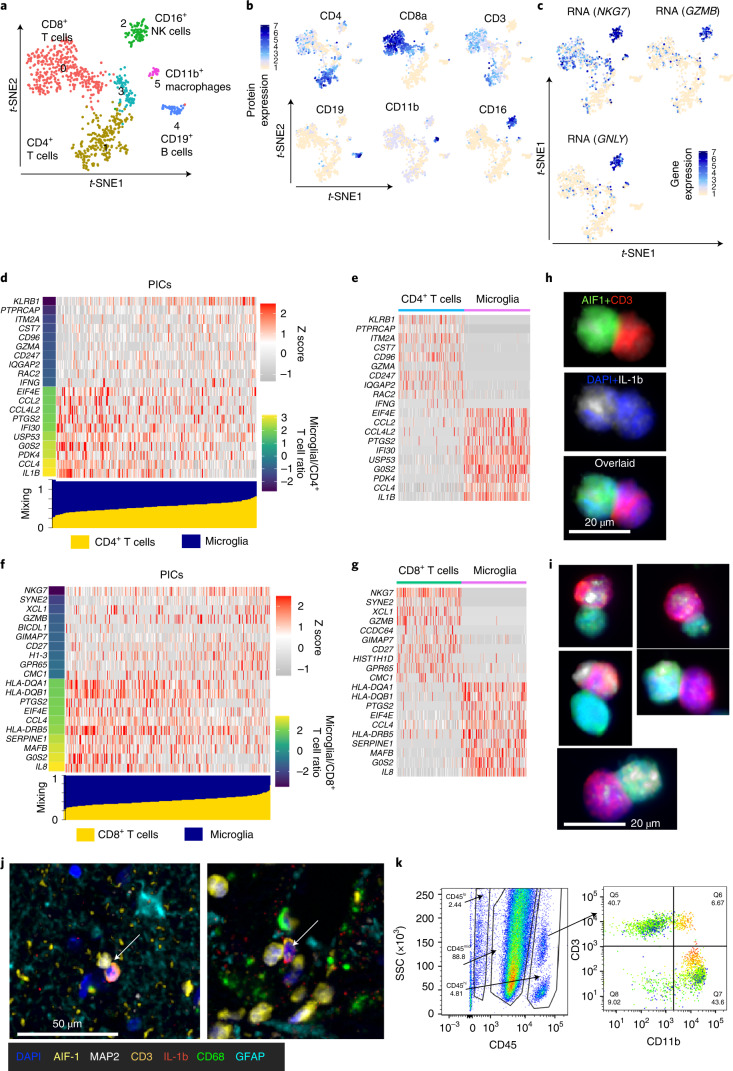
Direct interaction of microglia and infiltrating T cells in brain tissue from patients with refractory epilepsy. **a**, Doublet cell clusters are shown on the *t*-SNE map. Numbers and colors on the *t*-SNE map show the cluster ID. **b**, Surface epitope protein expression of major lineage markers are overlaid on the *t*-SNE map. **c**, Gene expression specific to NK cells is overlaid on the *t*-SNE map. **d**,**f**, Gene expression profile of physically interacting CD4^+^ T and microglial cells (**d**) and CD8^+^ T and microglial cells (**f**). The bar at the bottom (blue, microglia; yellow, T cells) shows the estimated mixing factor. Heatmap and mixing factor bars are ordered with increasing mixing factor value for T cells. Left, colored bar indicates the ratio of expected gene expression in microglia versus T cells. The top ten genes specific to T cells (lower microglial/T cell ratio) and specific to microglia (higher microglial/T cell ratio) are shown in the heatmap. **e**,**g**, Real gene expression values in microglia and T cells. Heatmaps were plotted for 500 randomly drawn cells, 250 each from microglia and T cells. **h**,**i**, Immune cells isolated from DRE tissue were formalin fixed, and cells were cytocentrifuged with Cytospin on slides for staining of CD3, AIF-1 and IL-1b. DAPI was used for the nuclear stain. Stained slides were imaged using a Vectra 3.0 imaging microscope. **h**, T cell (CD3^+^)–microglial (AIF-1) immune cell complex. **i**, Representative T cell–microglial immune cell complex producing IL-1b from the brain tissue of three patients with DRE. **j**, T cell–microglial immune cell complex from one of the FFPE tissue sections stained with a panel of six antibodies. A CD3^+^ T cell (orange) in physical interaction with an AIF-1^+^ microglia (yellow) is indicated with a white arrow, and IL-1b proteins are shown (red). **k**, Bivariate flow cytometry plot with gating for CD45, CD11b and CD3. Left, live gated cells with SSC on the *y* axis and CD45 expression on the *x* axis. Right, CD45^hi^-gated cells with CD3 expression on the *y* axis and CD11b expression on the *x* axis. CD115 levels were overlaid on the bivariate plot, where expression is indicated from low (green) to high (red). Flow cytometry analysis and plots were created using FlowJo software.

**Extended Data Fig. 9:**
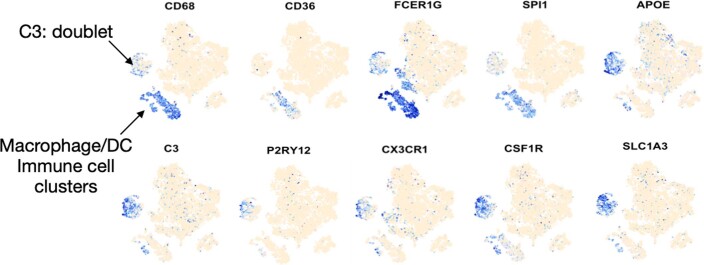
Microglia and macrophage specific genes. Gene expression were overload on t-SNE map to shows the expression of microglia and macrophage/DC specific cells and clusters.

**Extended Data Fig. 10:**
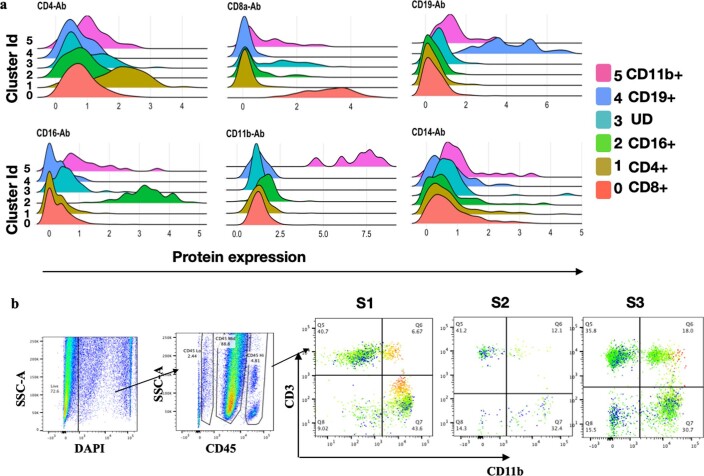
Phenotype of re-clustered doublets cells. (**a**) Protein expression density plot for major lineage marker proteins were visualized as overlaid density plots. Color fill shows the cluster identity. X-axis shows expression of proteins and y-axis shows cluster id. (**b**) Figure shows bivariate flow cytometry plots with gating strategy for doublets. Isolated immune cells from DRE brain tissue from 3 patients were stained with fluorochrome labeled antibodies and cells were analyzed using FACS ARIA II^TM^ Flow cytometer (BD biosciences). Flow cytometry analysis and plots were created using FlowJo^TM^ software. S1-S3 shows the doublet FlowJo gated plots for 3 samples analysed.

The combination of the following crucial elements: (1) a chemoattracting environment, which leads to an effective interactome (Fig. 3c,d and Extended Data Fig. 8), (2) production of pro-inflammatory cytokines from microglia (Fig. 2) and (3) brain infiltration of pro-inflammatory and cytotoxic immune cells (Fig. 3a,b) led to the next logical step of determining whether we could demonstrate direct interactions between infiltrating immune cells and microglia in the DRE microenvironment (DRE-M). These functional interactions would further elucidate the pivotal role for the DRE-M in maintaining and enhancing the pathogenic process. We discovered a mixed doublet cluster (cluster 3) by immune cell analysis (Fig. 3a,b). Cluster 3 clearly showed CD3, CD11b and HLA-DR surface protein expression, indicating doublets of T cells and microglia and/or macrophage cells. Transcriptomics coupled with epitope staining enabled us to clearly identify the doublets based on surface expression of lineage markers. Cluster 3 expressed microglial-specific genes, for example, *C3*, *CSF1R*, *APOE* and *CX3CR1*, while macrophage and DC immune cell clusters expressed higher levels of *CD68*, *FCER1G*, *SPI1* and *CD36* (Extended Data Fig. 9), clearly establishing a microglial phenotype for the doublet cluster. We further analyzed the components of these doublet clusters to segregate them into cell subsets of major immune cell lineages in direct interaction with microglia. We reclustered cluster 3 doublet cells using major lineage protein expression markers and obtained six clusters (Fig. 5a). Cluster phenotypes were identified using protein (Fig. 5b and Extended Data Fig. 10a) and gene (Fig. 5c) expression levels. A CD8^+^ T cell cluster (cluster 0) and a CD4^+^ T cell cluster (cluster 1) were the two main clusters (73% of the total doublet cells). We also observed B cell (cluster 4), NK cell (cluster 2) and macrophage clusters (cluster 5).

These results clearly showed microglial cells in direct contact with CD4^+^ and CD8^+^ T cells. As most doublets observed were microglial and T cell doublets, we examined the hypothesis that direct interaction between microglia and T cells reciprocally enhances their inflammatory function. We used a recently published method, sequencing of physically interacting cells (PICs) (PIC-seq)^30^, to find genes modulated due to physical interaction and to further dissect the gene signal specificity to the contributing interacting partner. The PIC-seq method estimates the mixing factor of contributing PICs and then calculates the expected counts of contributing cells and interacting cells. The *χ*^2^ test was performed to find significantly modulated genes using real versus expected counts of PICs. In microglial–CD4^+^ T cell PICs, 143 genes (Supplementary Table 3) were significantly modulated (FDR < 0.05), while, in microglial–CD8^+^ T cell PICs, 164 genes (Supplementary Table 4) were significantly modulated (FDR < 0.05). The differentially expressed genes were compared to their microglial and T cell expected contribution using relative distributions derived from both cell types. Each of the top ten genes from T cells and microglia are shown in a heatmap (Fig. 5d–g). We identified *CCL4* (encoding a chemokine) and *IL1B* (encoding a cytokine) from microglial cells, while *IFNG* and *GZMA* were from CD4^+^ T cells in CD4^+^ T cell–microglial PICs (Fig. 5d). CD8^+^ T cell–microglial PICs also upregulated *XCL1* (encoding a chemokine) and *GZMB* (encoding a cytotoxic product) as T cell genes, while *CCL4* (encoding a chemokine) and *IL8* (encoding a cytokine) were microglial genes (Fig. 5f). PIC dissection of signals suggests mutual enhancement of pro-inflammatory and cytotoxic function after physical interactions.

Finally, we validated the T cell–microglial doublet immune complex using IHC and flow cytometry (Fig. 5h–k). We clearly showed T cells (CD3^+^) in physical interaction with microglia (AIF-1^+^) and that the immune complex produced IL-1b (Fig. 5h–j). Indeed, based on PIC-seq (Fig. 5d–g) and IHC analysis (Fig.5h–j), microglial immune cell doublets clearly showed increased pro-inflammatory function when compared to microglia and T cells that were not in direct contact (Fig. 5d–i).

## Discussion

We used CITE-seq to characterize the DRE focus in the human brain as a pro-inflammatory microenvironment. Here, we characterized the immune milieu of DRE tissue and identified a potential mechanism of lymphocyte infiltration in the brain. Furthermore, we found physically interacting T cells with microglia. Using PIC-seq analysis, we further showed enhanced pro-inflammatory function in both the physically interacting microglia and T cells compared to cells not in direct physical interaction. Overall, microglial transcriptional similarity, infiltration of pro-inflammatory lymphocytes and direct interaction of microglia with T cells essentially place DRE closer to an immune-mediated disease, with many functional and transcriptional characteristics similar to MS^18,31^

Despite increasing evidence of neuroinflammation in animal models of epilepsy and human studies^4,6–8^, the benefits of anti-inflammatory therapy have been only anecdotally reported^9,10,12^, Treatment options for DRE reflect the limited knowledge on its etiopathogenesis. Hence, medical needs and scientific knowledge gaps coexist. These observations have led us to comprehensively investigate the general hypothesis that inappropriate, immunologically driven pro-inflammatory mechanisms contribute to the pathogenesis of DRE in humans, as in other brain inflammatory diseases such as MS and autoimmune encephalitis. In our previous study^8^, we employed a high-dimensionality mass cytometry, artificial intelligence-driven approach^32^ to examine the peripheral blood immunome in DRE in comparison with an age-matched standard dataset. We found DRE-specific aberrations, with an imbalance toward pro-inflammatory T cell subsets and a marked IL-17 signature^8^ as shown by another report^7^.

These aberrations raised the key question as to whether immune-mediated mechanisms directly affect the diseased tissue and, importantly, whether such immune-mediated inflammatory mechanisms are generated and sustained in the DRE-M. In this current work, we employed CITE-seq technology to comprehensively unravel the immune mechanism in the brain of patients with DRE.

CITE-seq allowed accurate identification of cellular identity and functional modality at single-cell resolution, with a clear separation between resident cells and infiltrating immune cells. Our analysis underscored microglia as a major source in inducing a pro-inflammatory microenvironment, both by primarily producing pro-inflammatory mediators and reduced microglial purinergic receptor-mediated signaling. Recent studies in mouse models demonstrated negative feedback control of neuronal activity by microglial purinergic (P2RY12) G protein-coupled receptor signaling^33,34^. Reductions in *P2RY12*-expressing microglia observed in DRE brain lesions (Extended Data Fig. 3) may lead to loss of negative feedback control and may contribute to hyperexcitability of neurons in an epileptic seizure. In a recent study, Badimon et al.^33^ showed enhanced expression of chemokine and motility genes upon selective activation of neurons in the mouse forebrain, indicating neuronal–microglial communication. Our data from patients with DRE show a similar expression profile in microglia, in which we observed an increase in pro-inflammatory, chemokine and motility-associated genes (Fig. 2 and Extended Data Fig. 3). Xu et al.^7^ had reported memory CD4^+^ and cytotoxic CD8^+^ T cells in human epileptic foci. We further extended this finding and showed transcriptional heterogeneity in T cell subsets and showed gene signatures specific to these T cell subsets. Increased frequency of T_H_1 and T_H_17 pro-inflammatory T cells from MS lesions is associated with disease activity^35,36^. T_H_1 and T_H_17 T cell signatures from DRE (Extended Data Fig. 5a) suggest a similarity in T cell-mediated signaling in MS and DRE. Single-cell transcriptomics further allowed us to study potential interactions with other cell subsets in the brain. Changes in leukocyte trafficking and blood–brain barrier permeability to immune cells have been implicated in human DRE and in animal models^37–39^ of seizure. In humans, improved epilepsy control has been reported in two patients with epilepsy and MS following treatment with natalizumab, a humanized anti-4 integrin antibody that mediates T cell migration in the brain and intestine^40^. In the mouse TLE model, blockade of colony-stimulating factor 1 receptor (CSF1R) was effective in attenuating seizures^29^. Our LR interactome network analysis supports and elucidates this complex relationship in the DRE brain. By developing and applying LR interactome network analysis, we unraveled the interface between resident and infiltrating cells. Within this interface, our new approach uncovered integrin–collagen-mediated interactions as the most common interaction mode between the immune and DRE resident cells. The integrin–collagen interaction was also validated by CellChat^28^ cellular interaction inference analysis (Supplementary Fig. 3). Integrin–collagen-mediated interactions in DRE and an effective anti-integrin therapeutic in MS^41,42^ further suggest functional similarity of T cells in MS and in DRE. Many inflammatory genes such as *IL1B*, *IL1A*, *TNF*, *CCL4*, *CCL2* and *IL15* were upregulated in the brain tissue of mice with TLE (Fig. 4 and Supplementary Table 2). Moreover, LR pairs enriched in human DRE were also upregulated in the brain of mice with TLE compared to control mouse brain tissue. Conservation of the LR interactome across species suggests its specificity to epilepsy.

Further, we clearly demonstrated a direct interaction between T cells and microglia inside epileptic brain tissue. We also showed enhanced pro-inflammatory activity in the directly interacting T cell–microglial immune complex. The microglia in direct contact with T cells showed an antigen-presenting cell phenotype that indicates a direct antigen-presenting role of microglia inside the brain. A previous study^43^ has reported colocalization of activated microglia and T cells in MS lesions and suggests physical interaction between them. This study shows a transcriptional profile of directly interacting microglia and T cells forming a mechanistically relevant stromal–immunological synapse within human brain tissue.

Our study was focused on brain-resident and infiltrating immune cells, and, in addition to a few key lineage surface markers, we analyzed cells at the transcriptional level. All the transcriptional-level information may not be translated to the protein level, and thus further validation at the protein level will be required before interventional therapeutic targeting. Astrocytes also contribute to neuroinflammation in epilepsy and are known to interact with and modulate cells of NVUs^44,45^. Whether a specific subset of astrocytes contributes to neuroinflammation in DRE and how it interfaces with other cells cannot be answered with our current data and is a limitation of the presented study. However, within these limitations, our dataset is a rich resource for future studies in neuroinflammation and may serve as a guide for interventional therapeutic development for DRE and possibly other neuroinflammatory diseases.

## Methods

### Selection of patients and acquisition of brain samples

Pediatric patients with DRE and who had a focal lesion amenable to surgical resection were identified through detailed seizure semiology, neuroimaging, electroencephalography monitoring studies and functional imaging (D.C.). The epileptogenic zone was identified in each patient, and epilepsy surgery was performed (D.C.Y.L.) to resect the epileptic brain to achieve seizure control. Histopathological examination was performed to identify etiologies such as neuronal migration disorders, cortical dysplasia, etc. A section of the excised sample was collected in RPMI medium and immediately processed to isolate brain-resident and infiltrating immune cells. Eleven samples were collected from six individual patients.

Patient clinical information is provided in Supplementary Table 5. Patients were recruited at the KK Women’s and Children’s Hospital, Singapore. The study was reviewed and approved by the SingHealth Central Institutional Review Board. Informed consent was obtained according to SingHealth Central Institutional Review Board requirements.

Control brain FFPE tissue sections were obtained from the UK Brain Bank. FFPE sections were from post-mortem brain tissue samples without neurological disorders.

### Isolation of human brain cells

Immune cells were isolated from brain tissues removed during brain surgery for the treatment of epilepsy. Only tissues from epileptic lesions were removed and used for the study. Within 2–6 h after resection, tissues were processed to isolate brain immune cells. Tissues were kept in cold RPMI medium with 10% serum during transportation. Immune cells were isolated as described previously^29^. Briefly, tissues were minced into small pieces and incubated with collagenase A (1 mg ml^−1^) and DNase 1 (100 U ml^−1^) for 1 h at 37 °C. Subsequently, digested tissue was mechanically dissociated using 5-ml syringe plungers and filtered through a 70-µm filter. Cells were washed with PBS and resuspended in 20 ml RPMI 10 medium. Percoll mix (10 ml; 9 ml Percoll and 1 ml 10× PBS) was added slowly through the wall of the tube on top of the cell suspension, and samples were centrifuged at 4,000 r.p.m. (3,750*g*) for 30 min with 0 acceleration and deceleration at 4 °C. Three layers appeared, and a middle layer that contained all the immune cells and microglia along with some other cells was collected, washed, counted and stored in freeze-mix medium (10% DMS in serum) for future use.

### CITE-seq library preparation and sequencing

PBMCs were thawed and stained with live–dead stain (Thermo Fisher Scientific). Live cells were then sorted using the FACSAria II SORP instrument (Becton Dickinson). A cocktail of 16 TotalSeq-B antibodies (BioLegend) was prepared at 1 µg ml^−1^, and staining was performed using the ‘TotalSeq-B with 10x Feature Barcoding Technology’ protocol according to the manufacturer’s recommendation. Single cells were encapsulated into droplets with a gel bead in the emulsion (GEM) method using the 10x Chromium controller. For gene expression and protein detection, the Chromium Single Cell 3ʹ Gene Expression protocol (version 3 chemistry, 10x Genomics) and the Chromium Single Cell 3ʹ Feature Barcode Library kit (10x Genomics) were used, respectively. Downstream library construction was performed using the 10x 3ʹ Gene Expression Library Construction kit, and barcoding was carried out with i7 Illumina adaptor indexes. Pooled libraries were then sequenced on the Illumina HiSeq 4000 platform using paired-end 151-bp reads to achieve 50,000 reads per cell for gene expression and 5,000 reads per marker for cell surface protein detection. Library construction and sequencing were performed in two batches (Supplementary Fig. 4).

### Multispectral immunohistochemistry and microscopy

For spatial analysis, brain tissues were fixed with 10% neutral buffered formalin, and paraffin blocks were prepared. A 5-µM-thin section was mounted on slides. For staining tissue sections, the Opal multiplexing assay was used, and imaged was performed with the Vectra 3 system^46^. Briefly, slides were deparaffinzed, and antigen retrieval was performed in citrate buffer (for anti-MAP2 antibody) or Tris–EDTA buffer (for all other antibodies). For protein stabilization and background reduction, goat serum (Dako) was used for blocking. Slides were then stained with the following antibodies: anti-AIF-1 (clone EPR16588, dilution 1:2,000, Abcam), anti-CD3 (polyclonal, dilution 1:300, Dako), anti-CD68 (clone PG-M1, dilution 1:50, Dako), anti-MAP2 (clone AP20, dilution 1:100, Thermo Fisher Scientific), anti-IL-1b (polyclonal, dilution 1:200, Thermo Fisher Scientific), anti-GFAP (clone GA5, 1:200, Thermo Fisher Scientific). Subsequently, slides were stained with anti-mouse or anti-rabbit secondary antibodies, and Alexa Fluor tyramides (PerkinElmer) from the Opal 7 color kit were used to detect antibody staining. Slides were counterstained with DAPI for 5 min, mounted with Glycergel (Dako) and imaged using the Vectra 3 imaging microscope.

### Cytospin sample preparation

Cells were fixed using 10% neutral buffered formalin (Sigma-Aldrich) for 1 h at 37 °C and washed with PBS. Cell were diluted to 1 × 10^6^ cells per ml and cytocentrifuged at 700 RCF for 30 min in a StatSpin Cytofuge 2. The slides were air dried for 20 min before they were used for multiplex IF staining. The slides were stained with the same primary antibodies at the same dilutions as the FFPE tissues.

### Flow cytometry

Cells were thawed and kept at 37 °C for 30 min. Cells were washed and suspended in ice-cold flow cytometry buffer (PBS supplemented with 0.2 mM EDTA and 0.5% BSA). Samples were stained using an antibody mix (anti-CD3–AF647, anti-CD45–AF488, anti-CD11b–PE-Dazzle and anti-CD115–PE). DAPI was used for live–dead staining. All antibodies were purchased from BioLegend. Cells were acquired using the FACSAria II instrument (BD Biosciences), and data were analyzed using FlowJo software (FlowJo).

### Single-cell transcriptomics and feature barcode analysis

Raw reads for transcriptome and protein markers were aligned to the human genome (hg19-3.0.0) using Cell Ranger version 3 software with feature barcoding methods; Cell Ranger’s count utility was used to count the features. Cell barcodes and feature count matrices were created by aggregating filtered feature counts of each sample using the Cell Ranger aggregate utility. Subsequent data normalization and analysis were performed using the Seurat R package and custom R code. Cell data were quality controlled and filtered based on cellular complexity (number of genes per cell) and mitochondrial reads. Cells with between 300 and 5,000 genes and mitochondrial percent reads less than 20 were kept for analysis. Data scaling, normalization, variable gene identification and clustering were performed using the Seurat pipeline. Principal-component (PC) analysis was performed on the 2,000 most variable genes, and the first 20 PCs were used for *t*-SNE and UMAP for data embedding into two dimensions. The nearest neighbor graph (SNN) was created from the first 20 PCs, and the SNN graph was used for clustering the cells. The cellular identity of the clusters was determined by finding cluster-specific genes using the FindMarkers function. This function implements the Wilcoxon rank-sum test to find the most differentially modulated genes in a cluster. Data were analyzed for batch effects by plotting cells on *t*-SNE coordinates (Supplementary Fig. 4a), where samples from both batches were mixed, and no batch effect was observed. We quantified the batch effect using Shannon entropy as described in the CellMixS R package^47^. Mean entropy of the transcript data was 0.91 and 0.92 for ADT data (Supplementary Fig. 4c,d). We observed that cells from a single cluster (indicated by the black arrow) had lower entropy values and contributed most to the batch effect. This cluster is for the most part from a single patient (P3.A and P3.B in Supplementary Fig. 4b), explaining its lower entropy value. Mean entropy was high and above 0.9 (0.91 and 0.92); therefore, no batch correction of raw data was performed.

### Ligand–receptor interaction network

To find the potential interaction between clusters, networks of clusters created based on known LR pairs were enriched in clusters. LR pairs from the dataset described by Ramilowski et al. were used^27^. The LR pairs that had literature-supported evidence (1,894 LR pairs) were included for network creation. To find ligand and receptor genes that were enriched in a cluster, we collapsed the single-cell profile into a bulk RNA-seq profile by summing the count of each gene, and then each cluster count was normalized to library size using the ‘library.size.normalize(data)’ function from the phateR package. This function normalizes for sequencing depth of each cluster. A combined matrix of normalized gene counts for 13 microglial and three NVU clusters and 16 immune cell clusters was created. A subset of normalized gene count matrices with genes that were in the LR pair gene list was kept for LR-enrichment analysis. Fold change for gene expression in each cluster was calculated in comparison to genes expressed in the rest of all clusters for each sample. Average fold change was calculated for each gene, and genes that showed log_2_ (fold change) greater than 1 were considered enriched ligand or receptor genes.

In the LR interaction network, clusters are nodes and, if two nodes contain an enriched LR pair, then a potential interaction between the nodes is established and an edge between the nodes is created. To represent the strength of the network, the number of enriched LR pairs between each pair of nodes is counted, and the count is represented as color and thickness of the edge in the network. Ligand-to-receptor signaling is indicated using the edge direction represented with an arrow. Arrow tail indicates the enriched ligand, and arrowheads show enriched receptors, thus clearly visualizing LR association with the clusters. To create, analyze and visualize the network, igraph, visNetwork and ggplot2 R packages were used.

### PIC-seq analysis

PICs were analyzed using PIC-seq methods^30^. Scripts provided by authors of the PIC-seq study were used for analyzing PICs; briefly, a gene-over-cell expression matrix for PICs and two background single-cell nonconjugated cells were fed as input in addition to a MetaCell^48^ background model for each singlet cell subset to the PIC-seq algorithm. The algorithm calculates mixing factor *α* using linear regression trained on simulated doublets. The algorithm also returns MetaCell assignment for each PIC. MetaCell IDs and mixing factors were subsequently used for estimating expected counts of PICs and singlet cells. The *χ*^2^ test was performed to find genes modulated in real PICs versus expected PIC counts.

### Mouse brain tissue RNA-seq analysis

Raw data (accession number PRJEB18790) in FASTQ format were downloaded from the ENA repository. QC on FASTQ data was performed using FastQC software, and sequences were aligned to the mouse genome (mm.GRCm38.97 version) using the STAR2 aligner with default parameters. Aligned BAM files were used for counting gene expression using the featureCounts utility in the subread R package. Differential gene expression was performed using the edgeR R package. The exact test as implemented in the edgeR package was used for differential gene expression analysis between mice with TLE and control mice. Genes with FDR < 0.05 and greater than 1-fold (100%) upregulated or 0.5-fold (50%) downregulated expression were considered significant.

### Statistics and reproducibility

For statistical programming and data visualization, R version 4.0.3 and R studio version 1.3 were used. The following R packages were used: Seurat (version 3.2), edgeR (3.32), igraph (1.26), visNetwork (2.0.9), CellChat (1.1), CiteFuse (1.1), ggplot2 (3.3.2). Appropriate statistical tests, procedures and software have been described in Methods. No statistical method was used to predetermine sample size, and no data were excluded from the analyses.

### Reporting summary

Further information on research design is available in the Nature Research Reporting Summary linked to this article.

## Online content

Any methods, additional references, Nature Research reporting summaries, source data, extended data, supplementary information, acknowledgements, peer review information; details of author contributions and competing interests; and statements of data and code availability are available at 10.1038/s41593-022-01095-5.

## Supplementary information

Supplementary Information Supplementary Figs. 1–4 and Tables 1–5.

Reporting Summary

## Data Availability

Raw count data are deposited in the GEO public repository (GEO accession number GSE201048), and raw counts and analyzed R objects are also available at https://epicimmuneatlas.org/NatNeu2022. Associated data used to produce figures are also deposited in the Zenodo repository (10.5281/zenodo.6477100).

## Code availability

Code used for the analysis and to produce figures is deposited in the Zenodo repository (10.5281/zenodo.6477100).
