# Supplementary material for: Single-cell transcriptomics and surface epitope detection in human brain epileptic lesions identifies pro-inflammatory signaling
Source: Nat Neurosci. 2022 Jun 23;25(7):956–66. doi: 10.1038/s41593-022-01095-5 (PMC9276529; doi:10.1038/s41593-022-01095-5)
Supplement: Supplementary file 1 — Supplementary Figs. 1–4 and Tables 1–5. [file 41593_2022_1095_MOESM1_ESM.pdf]

nature portfolio

https://doi.org/10.1038/s41593-022-01095-5

Supplementary information

Single-cell transcriptomics and surface epitope detection in human brain epileptic lesions identifies pro-inflammatory signaling

In the format provided by the
authors and unedited

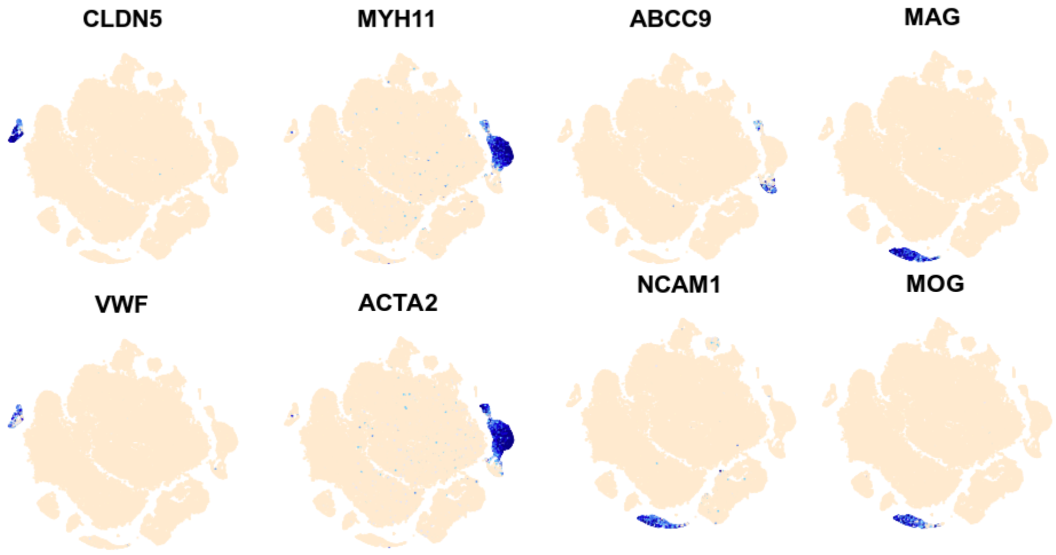

Supplementary Figure 1

Figure 1 —Phenotype of CD45-ve non-immune cells in the brain tissue

Gene expressions overlaid over t-SNE map to show the phenotype of CD45-ve non- immune cells. Cluster 13,20,22 together shows phenotype of cells from neurovascular unit (NVU). Cluster 22 shows expression of markers (CLDN5+ VWF+) specific to Endothelial cells (EC), Cluster 20 shows expression of markers (MYH11+ ACTA2+) specific to Pericytes (PC) and Cluster 13 shows expression of markers (ABCC9+) specific to Smooth Muscle cells (SMC). Cluster 18 expresses markers (MOG +MAG+) specific to Olgigodendrocytes (Oligo).

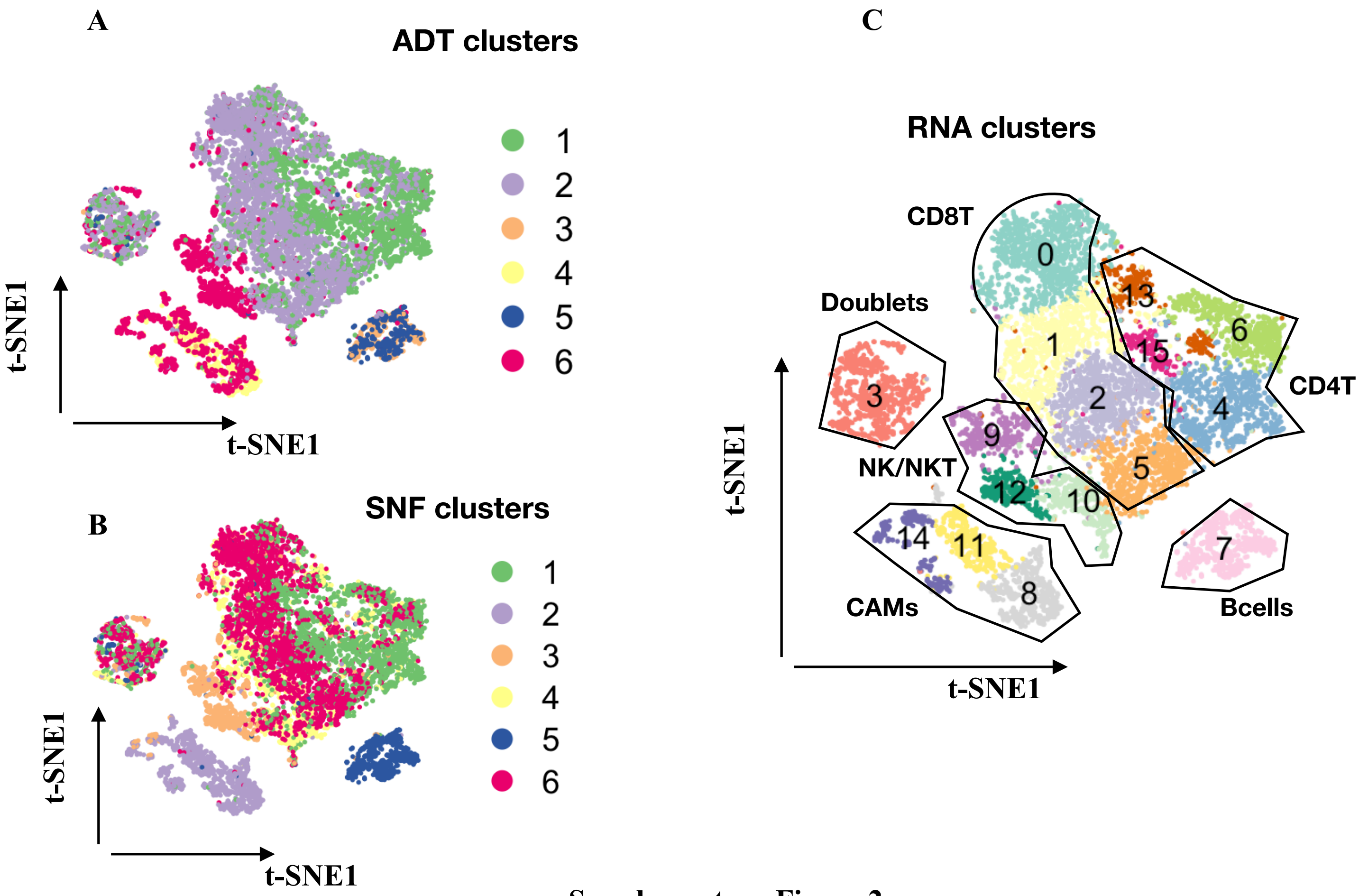

Supplementary Figure 2

Figure 2 — ADT and RNA expression Integrated clustering analysis

a, and **b** shows tSNE plot with overlaid cluster information. tSNE was performed on joint integrated ADT and RNA expression data. Similarity Network Fusion (SNF) algorithm was used to integrate the ADT and RNA data using CiteFuse R package. Spectral clustering was performed on integrated data (**a**) and ADT data (**b**). Optimal number of clusters was obtained using eigen value from SNF based clustering algorithm. (**c**) shows expression overlays of various protein levels on tSNE plots. (**d**) shows expression of mRNA levels corresponding to proteins in figure **d**.

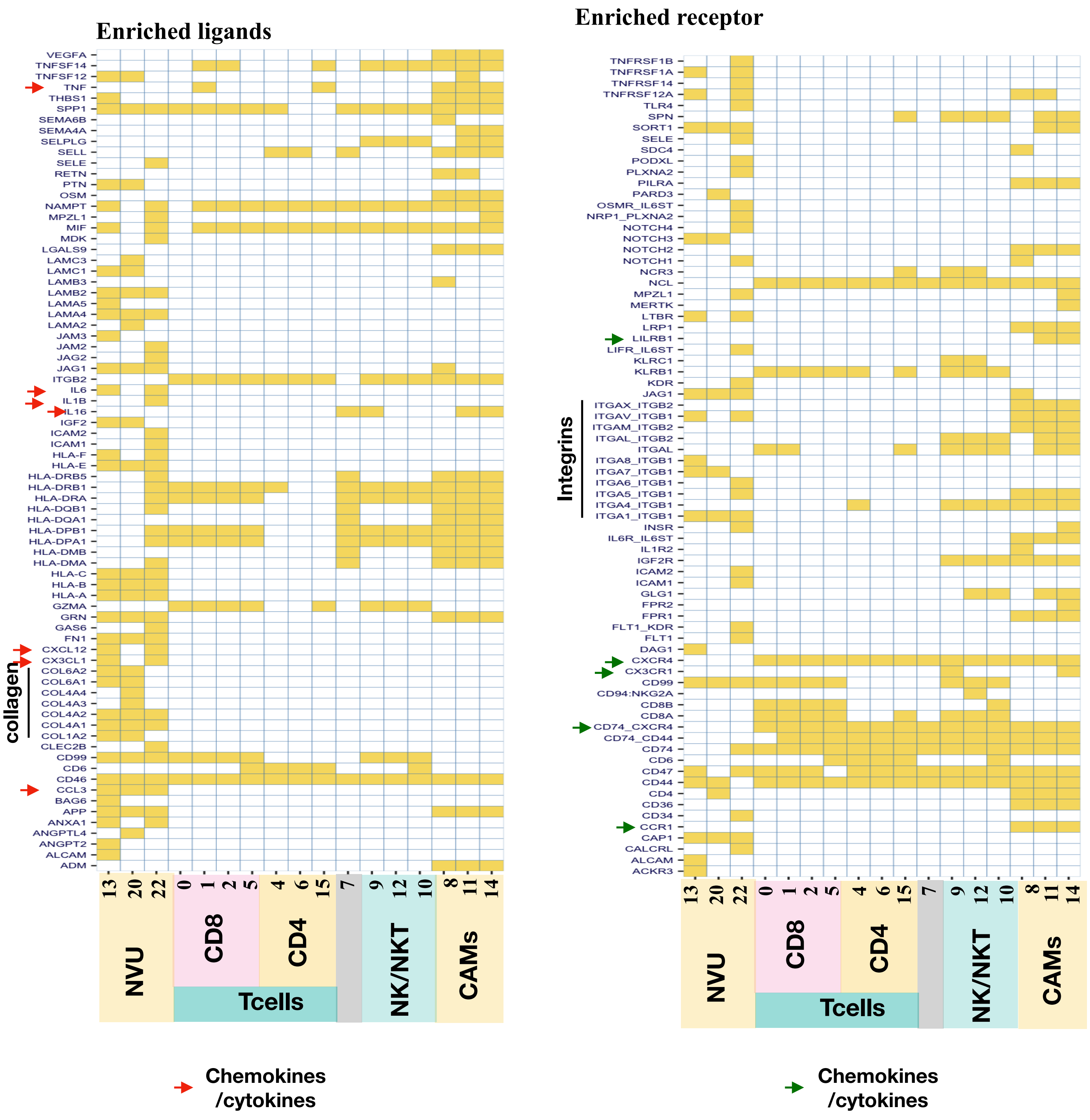

Supplementary Figure 3

Figure 3— CellChat analysis for enriched ligands and receptor in NVU and immune cell clusters

CellChat R package interaction analysis was performed to find the interaction between the NVU and immune cells clusters. Interacting ligands (**a**) and receptors (**b**) in each cluster were plotted as tilemap where filled rectangle (yellow color) shows enrichment of ligand/receptor in clusters indicated on x-axis

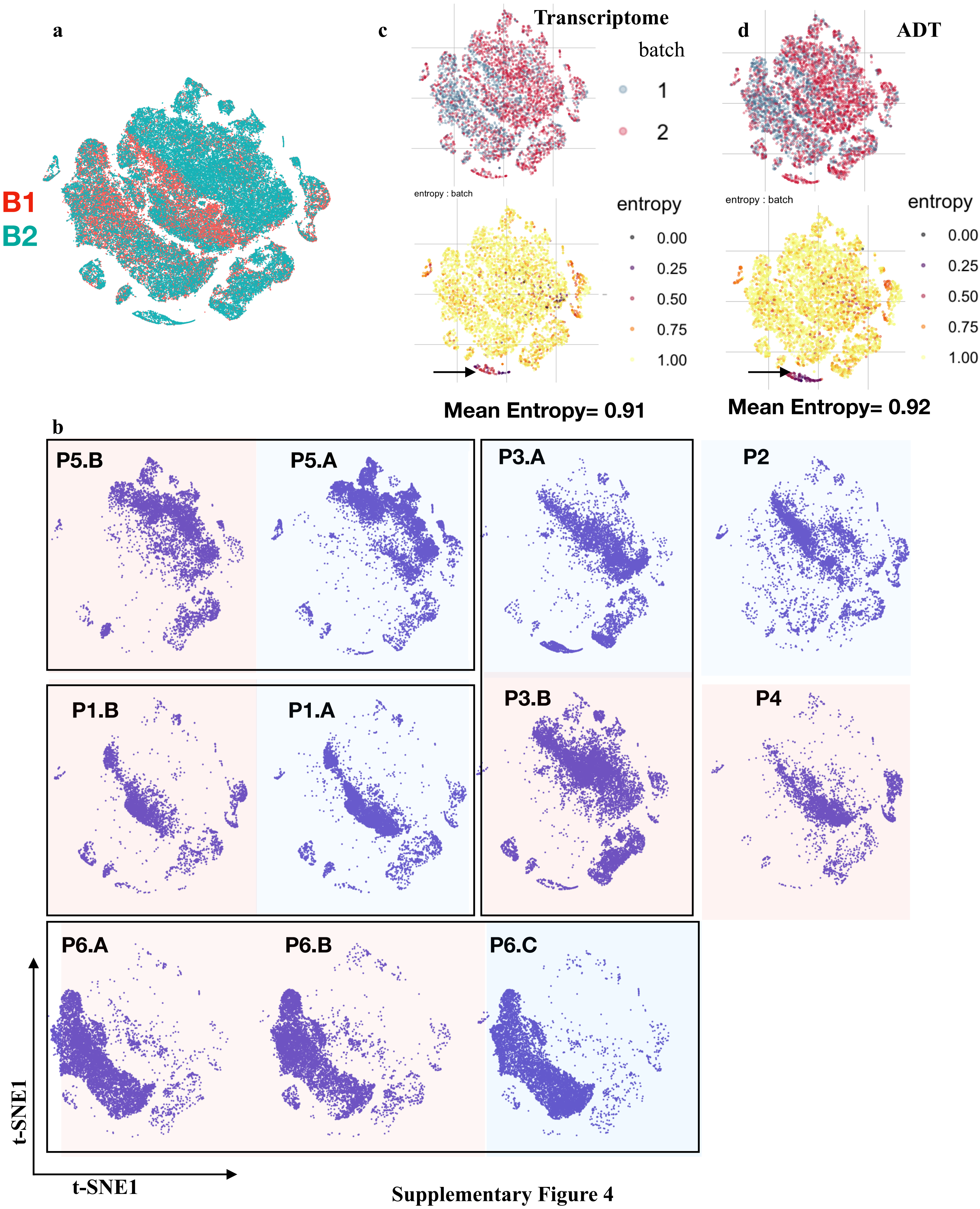

Supplementary Figure 4

Figure 4— Cells across samples and batch were plotted on t-SNE coordinates did not showed batch effect

11 samples from 6 patients were sequenced in 2 batches. (**a**) Sample distribution plot on t-SNE map showed mixed distribution of cells from batch 1 (B1) and batch 2 samples. (**b**) Each individuals tissue expression data was plotted shows tissue from same patients had similar expression profile across the batch. Batch 1 samples were shaded with light red background while batch 2 samples were shaded with light blue background. Tissues from same patients are shown in black box outline. (**c**) Shannon entropy for each batch variable was calculated to quantify the batch effect. Here 0 corresponds to low level of randomness in the data or higher batch effect. Mean entropy for transcript and ADT data were shown in the plots. Entropy value for each cell was overlaid on t-SNE plot.

Supplementary Table S1 (IL1b-P2RY12)

gene_name	p_val	avg_logFC	pct.1	pct.2	p_val_adj
P2RY12	0	1.23345125	0.719	0.423	0
ATM	0	1.215938255	0.764	0.465	0
SORL1	0	1.187349867	0.857	0.705	0
P2RY13	0	1.175604113	0.71	0.416	0
CX3CR1	0	1.174081717	0.751	0.541	0
MARCKS	0	1.121717878	0.915	0.848	0
A2M	0	1.074646631	0.876	0.776	0
IFNGR1	0	1.074182369	0.841	0.731	0
HTRA1	0	1.070556233	0.755	0.575	0
IFI44L	0	1.055384097	0.513	0.397	0
MAF	0	1.034042124	0.743	0.557	0
RN7SL1	0	1.029569065	0.472	0.289	0
GLA	0	-1.002604916	0.125	0.579	0
OSM	0	-1.008000146	0.059	0.561	0
STIP1	0	-1.01862186	0.168	0.796	0
MAP2K3	0	-1.019120912	0.089	0.722	0
EZR	0	-1.032374196	0.128	0.683	0
ZFP36	0	-1.033032454	0.443	0.922	0
ANKRD37	0	-1.034768286	0.103	0.602	0
SLC3A2	0	-1.036269133	0.275	0.86	0
DNAJB6	0	-1.052080402	0.543	0.961	0
TRIB1	0	-1.074507047	0.196	0.797	0
CTSL	0	-1.077282155	0.372	0.772	0
PPIF	0	-1.078737699	0.109	0.64	0
SERPINH1	0	-1.08027771	0.138	0.588	0
SIK1	0	-1.09396875	0.079	0.739	0
RILPL2	0	-1.112824556	0.206	0.77	0
FOSB	0	-1.11516272	0.643	0.987	0
ICAM1	0	-1.118840195	0.085	0.684	0
NEDD9	0	-1.123463657	0.097	0.708	0
MYADM	0	-1.139504495	0.177	0.796	0
DNAJA4	0	-1.140234832	0.409	0.883	0

Wilcoxon Rank Sum Test as implemented in FindMarker() function from Seurat R package was used for differential gene analysis

**CCL5**	0	-1.143578147	0.069	0.31	0
**PHLDA1**	0	-1.145725333	0.073	0.548	0
**DUSP1**	0	-1.147041886	0.75	0.992	0
**BCAS2**	0	-1.148205555	0.215	0.767	0
**RGCC**	0	-1.160869401	0.042	0.321	0
**HMOX1**	0	-1.16866354	0.434	0.843	0
**FKBP4**	0	-1.177570824	0.23	0.797	0
**GPR183**	0	-1.205065984	0.353	0.833	0
**ABL2**	0	-1.212006779	0.169	0.749	0
**DDIT3**	0	-1.215373333	0.209	0.815	0
**SOD2**	0	-1.22461842	0.54	0.956	0
**PDE4B**	0	-1.233547487	0.148	0.729	0
**CD69**	0	-1.23657026	0.093	0.588	0
**REL**	0	-1.240662481	0.696	0.986	0
**C5AR1**	0	-1.247952749	0.139	0.783	0
**IL1A**	0	-1.25299558	0.099	0.582	0
**PLEK**	0	-1.267928787	0.502	0.889	0
**EMP3**	0	-1.274759735	0.057	0.582	0
**MRPL18**	0	-1.296821951	0.241	0.817	0
**NR4A1**	0	-1.319359101	0.413	0.982	0
**INHBA**	0	-1.31954858	0.068	0.424	0
**ARID5B**	0	-1.320550272	0.189	0.679	0
**CREM**	0	-1.323246106	0.174	0.802	0
**SELK**	0	-1.332117315	0.301	0.876	0
**CXCR4**	0	-1.36120746	0.134	0.691	0
**KDM6B**	0	-1.364016576	0.2	0.901	0
**FTH1**	0	-1.371330473	0.946	0.999	0
**BAG3**	0	-1.379831085	0.469	0.932	0
**RRAD**	0	-1.396647094	0.056	0.399	0
**SQSTM1**	0	-1.416787611	0.415	0.921	0
**HSPH1**	0	-1.424353094	0.726	0.985	0
**RASGEF1B**	0	-1.432585909	0.15	0.835	0
**SRGN**	0	-1.450720655	0.748	0.992	0
**SOCS3**	0	-1.461890781	0.066	0.717	0

**TNFAIP3**	0	-1.483776011	0.21	0.805	0
**KLF4**	0	-1.494600966	0.17	0.85	0
**KLF6**	0	-1.505363003	0.714	0.991	0
**CSF1**	0	-1.515501018	0.073	0.565	0
**NFKB1**	0	-1.532723279	0.206	0.857	0
**LMNA**	0	-1.536936339	0.035	0.579	0
**BCL2A1**	0	-1.549222325	0.171	0.774	0
**SERPINE1**	0	-1.551354085	0.103	0.607	0
**CD83**	0	-1.552198598	0.611	0.996	0
**HSP90AB1**	0	-1.565863545	0.836	0.999	0
**CCL3**	0	-1.566679117	0.8	0.994	0
**PLIN2**	0	-1.57374083	0.2	0.724	0
**EIF4E**	0	-1.577771466	0.268	0.829	0
**NR4A2**	0	-1.595775973	0.34	0.952	0
**GRASP**	0	-1.622058126	0.067	0.777	0
**DNAJA1**	0	-1.629639535	0.659	0.99	0
**MT2A**	0	-1.653323799	0.236	0.593	0
**NR4A3**	0	-1.692434426	0.153	0.88	0
**HSPD1**	0	-1.813685696	0.742	0.987	0
**PLAUR**	0	-1.825519389	0.175	0.867	0
**CCL4**	0	-1.8852239	0.74	0.984	0
**IL8**	0	-1.922521159	0.496	0.91	0
**DUSP2**	0	-2.115542935	0.109	0.755	0
**ATF3**	0	-2.125679405	0.213	0.911	0
**IL1B**	0	-2.165356979	0.516	0.95	0
**G0S2**	0	-2.660971324	0.287	0.777	0

Exact Test as implemented in edgeR R package was used for TLE vs Control mice differential gene expression analysis

Supplementary Table S2 (DEGs in TLE vs Control mice)

logFC	logCPM	PValue	FDR	gene_id	gene_name
1.49212	5.75970	1.20336152949756E-234	2.04427056631046E-230	ENSMUSG00000075334	Rprm
1.76393	2.84970	1.73811316321922E-212	1.47635332083841E-208	ENSMUSG00000025572	Tmc6
1.66323	1.19602	2.2291073655426E-198	1.26226919752792E-194	ENSMUSG00000053399	Adamts18
3.18168	-1.06267	1.1902872880075E-194	5.05515011216785E-191	ENSMUSG00000009654	Oit3
1.68716	3.64232	2.94979281451898E-193	1.00222160666097E-189	ENSMUSG00000021478	Drd1
1.34329	5.54339	2.85869639090743E-167	6.93764775553362E-164	ENSMUSG00000031557	Plekha2
-2.20693	0.70673	1.92021168279655E-163	4.07756950841847E-160	ENSMUSG00000047686	Rtl3
-0.96585	3.98159	8.75141354299569E-163	1.65187792520456E-159	ENSMUSG00000024427	Spry4
1.58112	6.00687	1.19116135389856E-151	2.02354490800287E-148	ENSMUSG00000036907	C1ql2
1.30366	5.85589	1.04295860501148E-146	1.61070734381227E-143	ENSMUSG00000026834	Acvr1c
1.95946	0.73659	2.87962957348546E-142	4.07659559953092E-139	ENSMUSG00000109588	Lnp1
2.66614	6.48992	5.05327529761546E-141	6.60346467353011E-138	ENSMUSG00000073418	C4b
2.10047	2.02947	6.00048027684258E-140	7.28115421021441E-137	ENSMUSG00000031273	Col4a6
1.32321	1.78815	7.44099943208959E-131	7.90048114702112E-128	ENSMUSG00000029005	Draxin
2.05681	1.33496	8.05645627949689E-130	8.05076936918195E-127	ENSMUSG00000035279	Ssc5d
1.93613	3.86148	8.56082793738358E-130	8.07951916668179E-127	ENSMUSG00000001025	S100a6
1.73029	2.99250	6.40832292073407E-128	5.72971525144371E-125	ENSMUSG00000042436	Mfap4
2.61684	2.90459	8.65903515255695E-124	7.00474710341131E-121	ENSMUSG00000023918	Adgrf4
-1.35332	4.95651	2.31641034280028E-122	1.78868995015868E-119	ENSMUSG00000049892	Rasd1
1.82748	5.81506	6.57989748885465E-121	4.85996950176795E-118	ENSMUSG00000045573	Penk
1.85850	5.57930	2.75872201645741E-120	1.95271540064911E-117	ENSMUSG00000031375	Bgn
1.88729	1.16808	2.43245830600236E-119	1.65290406809473E-116	ENSMUSG00000025355	Mmp19
-1.39855	2.14710	1.70562966347155E-118	1.11443218165595E-115	ENSMUSG00000050534	Htr5b
2.08923	10.22311	1.63512758804137E-117	1.02879805428321E-114	ENSMUSG00000020932	Gfap
-0.74984	5.49258	6.20253185518199E-115	3.76316468413684E-112	ENSMUSG00000024867	Pip5k1b
-0.76432	6.92140	1.11250775917238E-114	6.51699372855874E-112	ENSMUSG00000029471	Camkk2
2.01047	2.27407	1.59123655776771E-114	9.01064221445261E-112	ENSMUSG00000056737	Capg
1.73544	2.90730	9.19165441742819E-113	5.03702662075065E-110	ENSMUSG00000035551	Igfbpl1
-0.85102	6.00425	5.2681323083256E-112	2.79671973918235E-109	ENSMUSG00000005483	Dnajb1
0.90432	4.64530	9.18011393725096E-111	4.72581138078846E-108	ENSMUSG00000045912	C2cd4c
1.35598	2.72856	1.98597600500743E-110	9.92287069796066E-108	ENSMUSG00000028108	Ecm1
2.30168	3.07708	4.86351197333201E-108	2.3606097543704E-105	ENSMUSG00000068699	Flnc
2.07398	7.03767	1.22675411067525E-106	5.7889163422642E-104	ENSMUSG00000026728	Vim
0.96672	1.24746	3.46088212560917E-104	1.58901258242834E-101	ENSMUSG00000027661	Slc2a10
3.24830	-0.18609	3.03538216063789E-102	1.35697558276096E-99	ENSMUSG00000045502	Hcar2
-0.95237	6.06378	3.45680108148561E-101	1.50574709672507E-98	ENSMUSG00000019960	Dusp6
1.57944	3.94756	7.82423951150339E-101	3.32295452053549E-98	ENSMUSG00000001119	Col6a1
0.99682	3.08697	4.92036905272283E-100	2.03871291384526E-97	ENSMUSG00000045382	Cxcr4
1.12072	3.18927	6.5408714630005E-100	2.64562677174887E-97	ENSMUSG00000020334	Slc22a4
2.04337	4.52317	9.0091478439815E-100	3.55924194357111E-97	ENSMUSG00000030111	A2m
2.79221	0.29802	2.21811046723498E-99	8.37361347053062E-97	ENSMUSG00000068117	Mei1
1.72227	2.89632	6.18245425491718E-99	2.26981233775022E-96	ENSMUSG00000035778	Ggta1
2.41344	2.61189	4.62881928531478E-98	1.63821629206099E-95	ENSMUSG00000046167	Gldn
2.29367	1.60284	4.97949256248585E-98	1.72635958472469E-95	ENSMUSG00000005124	Ccn4
-0.75672	2.17383	7.9245409605377E-98	2.69244203675229E-95	ENSMUSG00000027859	Ngf
1.55506	1.99820	1.41926278526773E-96	4.46489559187559E-94	ENSMUSG00000039457	Ppl
1.46754	-0.69046	2.36202911009643E-96	7.29566373133056E-94	ENSMUSG00000051054	1700014D04Rik
2.83747	-0.09081	1.70834009329893E-94	5.18237169731469E-92	ENSMUSG00000040706	Agmat

-0.75678	2.68968	2.5921296105697E-94	7.72545575865931E-92	ENSMUSG00000031872	Bean1
2.16418	0.09617	1.3214453526191E-93	3.80486672038869E-91	ENSMUSG00000029762	Akr1b8
1.90898	2.44559	4.20384398860355E-93	1.19024836130662E-90	ENSMUSG00000036198	Arhgap36
1.76790	-0.10266	1.22798672030391E-92	3.41984236139719E-90	ENSMUSG00000024210	Ip6k3
4.53264	1.33633	1.84414473105288E-91	4.89505167048848E-89	ENSMUSG00000039728	Slc6a5
1.29222	1.48098	3.3184953026692E-91	8.67301510796067E-89	ENSMUSG00000027315	Spint1
1.74238	4.08813	5.24435056338561E-91	1.34986405107265E-88	ENSMUSG00000029335	Bmp3
1.06089	5.25716	5.73992218402475E-90	1.43396761856195E-87	ENSMUSG00000059336	Slc14a1
1.62738	3.30902	7.3017044671374E-90	1.79770080417E-87	ENSMUSG00000090122	Kcne1l
1.12292	1.53372	7.93367765250945E-90	1.92539022801187E-87	ENSMUSG00000060572	Mfap2
2.83809	1.39418	9.23308473591306E-90	2.20917807737593E-87	ENSMUSG00000024907	Gal
2.15849	-0.87640	1.26533592964848E-89	2.98548982956506E-87	ENSMUSG00000079330	Lemd1
3.88054	-0.19519	2.33614302206705E-89	5.43649282998289E-87	ENSMUSG00000025496	Drd4
1.38064	3.37191	9.95202508025292E-89	2.28466219004509E-86	ENSMUSG00000032332	Col12a1
0.85780	2.95405	6.67816021247293E-88	1.49274454854592E-85	ENSMUSG00000031373	Car5b
-0.71520	2.40183	1.76018847208629E-87	3.83360022612846E-85	ENSMUSG00000031112	Stk26
1.26954	4.12298	2.26327481367437E-87	4.86690032084812E-85	ENSMUSG00000032231	Anxa2
1.48141	1.71218	9.03222743602959E-87	1.91799349604088E-84	ENSMUSG00000037406	Htra4
1.86912	1.78745	1.09917588358212E-86	2.30528393954235E-84	ENSMUSG00000041481	Serpina3g
1.57206	2.07661	1.48517541992319E-86	3.07684878459209E-84	ENSMUSG00000070469	Adamtsl3
2.65442	-0.80648	2.21080368682871E-86	4.52495578696942E-84	ENSMUSG00000050201	Otop2
-0.76730	1.70781	3.16105148293338E-86	6.39285030858004E-84	ENSMUSG00000029755	Dlx5
0.77925	3.70162	3.24406877949818E-86	6.48355769719E-84	ENSMUSG00000019027	Dnah1
0.77072	4.49089	3.43682083532793E-86	6.78892004076173E-84	ENSMUSG00000075224	Lrrc55
1.29725	3.02356	7.53395198130537E-86	1.45439518475472E-83	ENSMUSG00000051048	P4ha3
0.81576	4.40970	7.662255114525E-85	1.46254370657922E-82	ENSMUSG00000024713	Pcsk5
4.57206	2.13424	1.30477229562301E-84	2.4628301953382E-82	ENSMUSG00000030789	Itgax
-0.90379	5.91248	5.12135863602491E-84	9.56061983613089E-82	ENSMUSG00000031431	Tsc22d3
2.59934	4.44670	1.10307573403756E-83	2.03685332280762E-81	ENSMUSG00000026043	Col3a1
-1.26996	4.28326	2.21923791247873E-83	4.05380792012782E-81	ENSMUSG00000043439	Epop
1.62385	3.92706	2.54141792655936E-83	4.59293699323302E-81	ENSMUSG00000023224	Serping1
1.29699	1.73182	1.87763423810839E-82	3.35760530915635E-80	ENSMUSG00000039116	Adgrg6
0.83967	5.75494	7.33589576747994E-82	1.29814788852031E-79	ENSMUSG00000030256	Bhlhe41
3.67626	0.79393	4.98417538527072E-81	8.72898674690506E-79	ENSMUSG00000001131	Timp1
0.83160	2.58108	1.14314880762353E-80	1.98161346366414E-78	ENSMUSG00000046561	Arsj
1.60745	3.53245	1.36722988427263E-80	2.34611123980035E-78	ENSMUSG00000022146	Osmr
1.27688	1.59669	9.45834825713031E-80	1.6067842019213E-77	ENSMUSG00000018819	Lsp1
0.72196	3.41434	1.15841734066455E-79	1.94843502804054E-77	ENSMUSG00000001870	Ltbp1
3.35283	2.23125	1.29326588764318E-79	2.15392165679239E-77	ENSMUSG00000038224	Serpinf2
6.52021	2.00812	2.22165842865394E-79	3.63469155132865E-77	ENSMUSG00000068129	Cst7
0.95450	0.93767	2.22514669965964E-79	3.63469155132865E-77	ENSMUSG00000039616	Mocos
2.51490	3.41382	5.30806951650068E-79	8.58795094726795E-77	ENSMUSG00000029816	Gpnmb
1.86566	1.81794	7.17657554969367E-79	1.15014778715279E-76	ENSMUSG00000037686	Aspg
-0.94760	6.15423	7.90565556641207E-79	1.25515211927297E-76	ENSMUSG00000026826	Nr4a2
1.44313	0.90241	1.99971299722563E-78	3.11661691714394E-76	ENSMUSG00000063727	Tnfrsf11b
0.87683	5.58345	3.50858189734557E-78	5.41852629746423E-76	ENSMUSG00000027712	Anxa5
1.43407	-0.17757	4.85462721986735E-78	7.42976641541501E-76	ENSMUSG00000034918	Cdhr2
1.12334	3.98572	1.53135745037344E-77	2.32274110419143E-75	ENSMUSG00000068220	Lgals1
1.16116	2.68825	2.17330024247183E-77	3.23859864202733E-75	ENSMUSG00000029994	Anxa4
1.31567	3.12595	2.88643959031169E-77	4.26389876175782E-75	ENSMUSG00000031274	Col4a5
1.78468	2.18458	3.39531661336979E-77	4.97238264033845E-75	ENSMUSG00000026656	Fcgr2b

0.93724	2.32284	5.63885853004943E-77	8.18742980414357E-75	ENSMUSG00000046768	Rhoj
0.78602	3.38705	7.55663941142027E-77	1.08789991797634E-74	ENSMUSG00000028413	B4galt1
2.83670	-0.33626	2.8456820266416E-76	3.99524349327169E-74	ENSMUSG00000001020	S100a4
0.89374	4.90068	4.426680098378E-76	6.16397061567585E-74	ENSMUSG00000032440	Tgfbr2
1.18069	3.47557	4.6351855488441E-76	6.40183187835476E-74	ENSMUSG00000023885	Thbs2
2.22531	4.23475	8.09666732163214E-76	1.1092434230636E-73	ENSMUSG00000005087	Cd44
2.06344	4.53718	1.06409245735268E-75	1.44614421324058E-73	ENSMUSG00000001506	Col1a1
0.73866	1.13985	1.20962158331656E-75	1.62813966368635E-73	ENSMUSG00000036292	Gramd1c
1.16914	4.82950	3.54110881549078E-75	4.66328345407422E-73	ENSMUSG00000000753	Serpinf1
2.06282	1.75277	4.56000405843466E-75	5.95887299574523E-73	ENSMUSG00000040552	C3ar1
3.88559	-0.32890	5.80984076545458E-75	7.47708900935927E-73	ENSMUSG00000025469	Msx3
1.26240	-0.03002	8.01357445819245E-75	1.01592987235652E-72	ENSMUSG00000026387	Sctr
1.56426	-0.51543	1.41853953686795E-74	1.78504812239354E-72	ENSMUSG00000026073	Il1r2
1.45615	0.52188	2.2964571096973E-74	2.82697198402448E-72	ENSMUSG00000020169	Best3
2.42763	1.71217	4.02528986426179E-74	4.91954131036541E-72	ENSMUSG00000024529	Lox
4.40506	0.66288	5.20583278800441E-74	6.3169062430442E-72	ENSMUSG00000022218	Tgm1
1.67352	1.86297	8.37740825425495E-74	1.00932915903038E-71	ENSMUSG00000031465	Angpt2
1.27921	2.88628	1.11797765578485E-73	1.32812618297014E-71	ENSMUSG00000020256	Aldh1l2
1.14224	2.91947	2.71818137224156E-73	3.18458380356136E-71	ENSMUSG00000028883	Sema3a
4.32401	1.14834	2.90291866000369E-73	3.37772480795498E-71	ENSMUSG00000004552	Ctse
1.01186	3.68403	3.16158701594257E-73	3.65367620590696E-71	ENSMUSG00000017057	Il13ra1
0.80582	3.56835	5.4383845396202E-73	6.24238355128838E-71	ENSMUSG00000015085	Entpd2
1.81692	0.71634	6.30595830978377E-73	7.18963891051052E-71	ENSMUSG00000072812	Ahnak2
1.07634	4.18606	1.04535687025492E-71	1.1606877458752E-69	ENSMUSG00000023046	Igfbp6
1.18704	3.07008	1.55456352763057E-71	1.71486527320702E-69	ENSMUSG00000020363	Gfpt2
0.98422	5.46712	3.82131950856972E-71	4.18816618139242E-69	ENSMUSG00000026437	Cdk18
-1.43005	0.58412	4.20249484489413E-71	4.57640912981163E-69	ENSMUSG00000028971	Cort
3.73733	-0.31679	4.4514614562178E-71	4.81665141517376E-69	ENSMUSG00000000263	Glra1
1.09216	2.97605	1.55147808649238E-70	1.63705029399581E-68	ENSMUSG00000050666	Vstm4
3.41856	-0.26850	4.11792390696333E-70	4.3182278599687E-68	ENSMUSG00000004707	Ly9
-0.82805	3.14024	7.78165348434953E-70	8.11010609767668E-68	ENSMUSG00000049511	Htr1b
2.24904	-0.62714	1.205642704634E-69	1.24886940648307E-67	ENSMUSG00000067597	Dgat2l6
3.86758	-0.73112	8.95919106381639E-69	9.05944867810196E-67	ENSMUSG00000026285	Pdcd1
0.90565	1.72513	1.07846751711484E-68	1.08408320596136E-66	ENSMUSG00000019832	Rab32
2.63089	4.58158	1.21968139291286E-68	1.21882044134139E-66	ENSMUSG00000069516	Lyz2
1.94620	3.64812	1.24285638324333E-68	1.22753745572893E-66	ENSMUSG00000026247	Ecel1
1.66699	0.23962	1.47968273869935E-68	1.45299713092627E-66	ENSMUSG00000074570	Cass4
1.04144	3.55655	3.23541242966795E-68	3.15880381351719E-66	ENSMUSG00000027015	Cybrd1
1.45396	1.31161	4.11122517586732E-68	3.99094247357908E-66	ENSMUSG00000040212	Emp3
1.42220	0.80279	4.53508933335412E-68	4.37739190880794E-66	ENSMUSG00000006219	Fblim1
0.70323	2.31296	4.7625585529993E-68	4.57097992646057E-66	ENSMUSG00000042961	Egflam
0.83210	1.05105	5.67023217733732E-68	5.38133543176572E-66	ENSMUSG00000044006	Cilp2
1.33552	1.48310	7.48982697578457E-68	7.06873225914601E-66	ENSMUSG00000023031	Cela1
1.19041	1.49298	8.09211828565598E-68	7.5949671512002E-66	ENSMUSG00000033633	Clec18a
1.08067	3.27669	1.147883577222E-67	1.07144209944216E-65	ENSMUSG00000029070	Mxra8
1.16403	-0.13349	1.35996807374323E-67	1.25560530634511E-65	ENSMUSG00000032271	Nnmt
0.74531	2.27180	1.47452422954781E-67	1.35401176278693E-65	ENSMUSG00000036390	Gadd45a
0.76006	4.46965	1.92671912845084E-67	1.7597368039851E-65	ENSMUSG00000021136	Smoc1
0.93925	1.21660	4.41256080089956E-67	4.00858732008993E-65	ENSMUSG00000021319	Sfrp4
1.80877	4.83880	6.68213828390095E-67	6.00614630512748E-65	ENSMUSG00000020473	Aebp1
0.94203	4.75065	1.08924039897366E-66	9.73895573566554E-65	ENSMUSG00000048332	Lhfp

0.84357	2.02925	1.38052812984839E-66	1.22787496700861E-64	ENSMUSG00000040093	Bmf
1.30446	1.25257	1.91880987870297E-66	1.69774699059407E-64	ENSMUSG00000047407	Tgif1
1.00416	5.62528	3.23134240118116E-66	2.8007165669013E-64	ENSMUSG00000031207	Msn
-0.82049	5.03011	4.0141518159526E-66	3.46154370809151E-64	ENSMUSG00000027270	Lamp5
0.88773	4.41786	4.67745484932922E-66	4.01316176668711E-64	ENSMUSG00000022912	Pros1
1.77389	0.09737	4.91155968689571E-66	4.19284301311479E-64	ENSMUSG00000047497	Adamts12
1.15441	3.80571	8.76180115603911E-66	7.44227390193962E-64	ENSMUSG00000020388	Pdlim4
-0.88890	4.75517	1.26138856478181E-65	1.06609298201559E-63	ENSMUSG00000027004	Frzb
1.02654	5.12833	1.49992399927679E-65	1.26142123265911E-63	ENSMUSG00000007655	Cav1
3.25681	0.78421	1.52056716037207E-65	1.27248250839413E-63	ENSMUSG00000026535	Ifi202b
0.76602	2.43777	2.53224879620303E-65	2.10871777205378E-63	ENSMUSG00000032744	Heyl
1.14294	0.08955	3.1297020144063E-65	2.58094067090943E-63	ENSMUSG00000032860	P2ry2
1.00338	2.72331	7.18272169382077E-65	5.86634981416477E-63	ENSMUSG00000028497	Hacd4
0.72329	2.92311	1.0624049948643E-64	8.5943505013118E-63	ENSMUSG00000029826	Zc3hav1
1.07818	2.37282	3.93068584739025E-64	3.14974014978611E-62	ENSMUSG00000036334	Igsf10
3.97926	-0.58605	6.68776046770809E-64	5.2842639453686E-62	ENSMUSG00000030577	Cd22
0.87849	3.82642	2.76052849453565E-63	2.16109945000791E-61	ENSMUSG00000007041	Clic1
3.61062	-1.26482	7.82858763808567E-63	5.99063273854952E-61	ENSMUSG00000063232	Serpina11
2.21122	1.01265	1.16859707841399E-62	8.86255677147179E-61	ENSMUSG00000027074	Slc43a3
1.96101	-0.06622	1.99552670867012E-62	1.50666701008391E-60	ENSMUSG00000031212	Pgr15l
1.94046	4.53735	2.40081305856252E-62	1.8046465592416E-60	ENSMUSG00000067586	S1pr3
1.43501	2.19172	2.79982521872943E-62	2.08611538665682E-60	ENSMUSG00000021903	Galnt15
-1.04606	4.20354	3.1661895886381E-62	2.33857516225148E-60	ENSMUSG00000024190	Dusp1
1.67404	0.48363	4.64126420089094E-62	3.41323793267252E-60	ENSMUSG00000028076	Cd1d1
1.14859	2.23196	5.09217807996011E-62	3.72870350096389E-60	ENSMUSG00000023249	Parp3
0.90636	2.70481	8.32084006884013E-62	6.06671378066336E-60	ENSMUSG00000025789	St8sia2
-1.58186	7.05750	1.88180922966709E-61	1.35458369464341E-59	ENSMUSG00000038418	Egr1
0.91527	3.82330	2.23562022112459E-61	1.60247748170736E-59	ENSMUSG00000036908	Unc93b1
1.75568	5.13748	2.56422744973987E-61	1.81504566317421E-59	ENSMUSG00000029661	Col1a2
-0.88199	2.49841	2.7677573652701E-61	1.95098183075554E-59	ENSMUSG00000024957	Kcnk4
0.99933	4.16323	3.04781319417977E-61	2.13951448523661E-59	ENSMUSG00000005397	Nid1
1.36331	1.34804	4.25929874787403E-61	2.96544947249524E-59	ENSMUSG00000027907	S100a11
1.42740	-0.20467	5.17839449820634E-61	3.59063533614405E-59	ENSMUSG00000056880	Gadl1
1.41810	0.03113	8.43517353818209E-61	5.82507024661128E-59	ENSMUSG00000039476	Prrx2
2.70041	1.54901	8.59077047211249E-61	5.90850237976709E-59	ENSMUSG00000027832	Ptx3
0.74866	2.68564	9.27584456365619E-61	6.35395352610449E-59	ENSMUSG00000001555	Fkbp10
1.03370	1.06897	1.08587135025899E-60	7.37871299927988E-59	ENSMUSG00000023411	Nfatc4
0.72157	4.89515	1.14452890029308E-60	7.74631751321867E-59	ENSMUSG00000039831	Arhgap29
1.14718	3.03274	1.23616991264556E-60	8.33335495080268E-59	ENSMUSG00000028270	Gbp2
1.58855	2.59964	3.23769283084657E-60	2.17398916246725E-58	ENSMUSG00000031825	Crispld2
1.08484	3.83411	3.41935622288624E-60	2.28511607515089E-58	ENSMUSG00000020467	Efemp1
1.16104	3.47949	3.4300953565074E-60	2.28511607515089E-58	ENSMUSG00000061132	Blnk
2.36113	-1.68874	3.56990529261888E-60	2.36896684027381E-58	ENSMUSG00000001021	S100a3
0.97160	3.83530	5.42642569686354E-60	3.54554306685838E-58	ENSMUSG00000030341	Tnfrsf1a
1.90394	0.06394	5.76164816583475E-60	3.73583507790843E-58	ENSMUSG00000031995	St14
-1.59799	4.55471	1.00735613410916E-59	6.43344586700994E-58	ENSMUSG00000071341	Egr4
1.14301	3.57265	1.02628937097156E-59	6.52981417006172E-58	ENSMUSG00000026676	Ccdc3
1.29284	4.51193	1.03713173129484E-59	6.57417681016295E-58	ENSMUSG00000024517	Grp
1.58980	3.41887	2.36099373378717E-59	1.46381611494805E-57	ENSMUSG00000018774	Cd68
1.61261	1.56425	3.85022049076301E-59	2.34435647659792E-57	ENSMUSG00000026177	Slc11a1
1.53067	0.00822	4.04110596138522E-59	2.45179671685758E-57	ENSMUSG00000026548	Slamf9

1.37704	0.12009	7.48380594801571E-59	4.47658082552433E-57	ENSMUSG00000040428	Plekha4
1.84560	3.31078	8.5419357410435E-59	5.0737903625471E-57	ENSMUSG00000030124	Lag3
-1.09151	6.78191	1.39909993395791E-58	8.25274641599893E-57	ENSMUSG00000028341	Nr4a3
2.09657	1.58561	2.06861087575036E-58	1.21177798473266E-56	ENSMUSG00000015134	Aldh1a3
1.71901	1.11569	2.93503071521271E-58	1.70171678464278E-56	ENSMUSG00000031070	Mrgprf
1.55013	-0.27589	4.32246952427888E-58	2.48074703643411E-56	ENSMUSG00000000889	Dbh
1.33597	2.32349	5.25904270086156E-58	3.00810159603489E-56	ENSMUSG00000026042	Col5a2
1.48691	0.16940	5.39451941991578E-58	3.07523811763521E-56	ENSMUSG00000025887	Casp12
2.18981	-0.49160	6.49274375105845E-58	3.6889207639793E-56	ENSMUSG00000022479	Vdr
1.26965	2.05090	6.81576138570013E-58	3.85953848067579E-56	ENSMUSG00000029163	Emilin1
1.13207	3.03463	7.67326607774907E-58	4.33067920693692E-56	ENSMUSG00000004730	Adgre1
2.54181	1.90082	7.91818910979542E-58	4.45411247010611E-56	ENSMUSG00000050063	Klk6
0.99941	0.18448	8.63599062239089E-57	4.81010520305497E-55	ENSMUSG00000063652	Slc22a21
0.85217	1.91222	1.08078365907624E-56	6.00011529424417E-55	ENSMUSG00000035873	Pawr
1.12420	5.35841	2.12559094617892E-56	1.16482383850605E-54	ENSMUSG00000026365	Cfh
-0.98759	1.21988	3.14937038919734E-56	1.72030560037571E-54	ENSMUSG00000054204	Alkal2
0.90001	1.24770	3.45181759701296E-56	1.8794704275018E-54	ENSMUSG00000031595	Pdgfrl
-1.51362	-1.41144	4.00724920218639E-56	2.17492490245184E-54	ENSMUSG00000029306	Ibsp
0.81789	3.48307	4.14404655290802E-56	2.24200837072616E-54	ENSMUSG00000026389	Steap3
-1.12464	2.37952	4.54278467527496E-56	2.44993098614511E-54	ENSMUSG00000032807	Alox12b
1.22589	2.42825	5.03501207049889E-56	2.7067969953682E-54	ENSMUSG00000026395	Ptprc
0.94735	2.72226	1.01096950966265E-55	5.41777603474732E-54	ENSMUSG00000045930	Clec14a
1.55552	0.72693	1.22124845535454E-55	6.52407822627765E-54	ENSMUSG00000015533	Itga2
1.32853	0.03480	1.61593293154277E-55	8.57858395032766E-54	ENSMUSG00000118219	AC124108.1
1.17530	3.85342	1.83922369060371E-55	9.72645092476848E-54	ENSMUSG00000022665	Ccdc80
0.70445	1.87233	1.84360560264625E-55	9.72645092476848E-54	ENSMUSG00000062312	Erbb2
1.23521	3.00349	2.4991350095917E-55	1.31440574436358E-53	ENSMUSG00000020241	Col6a2
1.08948	0.70647	2.58675919065505E-55	1.35629213366815E-53	ENSMUSG00000057948	Unc13d
-0.82033	7.11760	2.86006438335145E-55	1.49039183264952E-53	ENSMUSG00000026841	Fibcd1
0.79201	4.32563	2.89005199514693E-55	1.50141294475706E-53	ENSMUSG00000020846	Rflnb
0.82921	1.81059	3.93232879589403E-55	2.03046813327197E-53	ENSMUSG00000040219	Ttc12
1.44433	1.11818	4.07821236463305E-55	2.09941429243594E-53	ENSMUSG00000018566	Slc2a4
1.54383	-1.40380	4.6020335993126E-55	2.36191380015476E-53	ENSMUSG00000030562	Nox4
1.00073	1.39406	5.00869032750899E-55	2.55518412263432E-53	ENSMUSG00000038732	Mboat1
3.38306	-2.22072	5.09995891843646E-55	2.59395515288619E-53	ENSMUSG00000027484	Bpifa5
1.39612	2.75535	5.16461497089492E-55	2.61174335523972E-53	ENSMUSG00000036854	Hspb6
1.05232	1.98801	5.16568028820665E-55	2.61174335523972E-53	ENSMUSG00000022610	Mapk12
1.85795	1.05274	5.79303432890507E-55	2.91159962069347E-53	ENSMUSG00000000682	Cd52
0.82593	3.45020	7.35469905025012E-55	3.67475374898968E-53	ENSMUSG00000033488	Cryzl2
1.09008	3.54539	9.41168141259003E-55	4.6750188256456E-53	ENSMUSG00000024087	Cyp1b1
-1.59520	5.83290	1.42660200462948E-54	7.06563115295789E-53	ENSMUSG00000023034	Nr4a1
0.84799	2.60181	1.43203798955314E-54	7.07193644375837E-53	ENSMUSG00000000628	Hk2
0.74284	2.91242	1.6426934633901E-54	8.08871784233943E-53	ENSMUSG00000022574	Naprt
1.92466	-1.42355	1.96517095255859E-54	9.64864859597266E-53	ENSMUSG00000002228	Ppm1j
1.35840	2.68500	2.10383456947972E-54	1.02996950047036E-52	ENSMUSG00000039899	Fgl2
1.00881	7.94693	3.29548612097532E-54	1.59953480637511E-52	ENSMUSG00000021091	Serpina3n
1.48058	1.78395	4.47415058278248E-54	2.15928608239514E-52	ENSMUSG00000064294	Aox3
1.57929	0.03767	5.24189672456615E-54	2.50843215653323E-52	ENSMUSG00000041361	Myzap
1.51320	3.86639	7.34744808882704E-54	3.49631507375333E-52	ENSMUSG00000046352	Gjb2
0.81017	4.95844	8.22312942421596E-54	3.90208163850784E-52	ENSMUSG00000063632	Sox11
1.81114	1.97552	9.51837564703728E-54	4.50412717247547E-52	ENSMUSG00000038147	Cd84

1.18972	1.55232	1.00333011989357E-53	4.73460335465333E-52	ENSMUSG00000039891	Txlnb
1.38613	1.22365	1.36937330251222E-53	6.39091034699932E-52	ENSMUSG00000050335	Lgals3
1.34124	1.12657	2.71967659883233E-53	1.25548549078706E-51	ENSMUSG00000018920	Cxcl16
1.82042	0.99910	3.41401554273879E-53	1.57174244010966E-51	ENSMUSG00000079014	Serpina3i
1.12140	-0.27583	6.41229460004891E-53	2.93617414193075E-51	ENSMUSG00000046916	Myct1
1.53277	1.63893	6.67544083731148E-53	3.04027852397446E-51	ENSMUSG00000021624	Cd180
1.77717	-0.94938	7.53327466706498E-53	3.41267386784266E-51	ENSMUSG00000039079	Trhr2
0.84724	2.71656	7.55929600982847E-53	3.41535427167463E-51	ENSMUSG00000019846	Lama4
-0.72994	4.74004	8.30784421977985E-53	3.74359834497666E-51	ENSMUSG00000036578	Fxyd7
1.91291	-0.36103	8.98746854839485E-53	4.03913004497703E-51	ENSMUSG00000043157	Arl11
1.26221	1.23402	1.07168869135195E-52	4.77843766107267E-51	ENSMUSG00000029134	Plb1
1.23310	1.31689	3.51683972830875E-52	1.53979570372446E-50	ENSMUSG00000040613	Apobec1
1.02785	3.60301	4.1071950999682E-52	1.7890520604682E-50	ENSMUSG00000025492	Ifitm3
2.51624	-1.54360	4.98566121497317E-52	2.16062277346847E-50	ENSMUSG00000021886	Gpr65
0.72681	3.85568	7.59739707831959E-52	3.25099701678824E-50	ENSMUSG00000033207	Mamdc2
0.75589	0.96353	8.69444083717983E-52	3.70178348225591E-50	ENSMUSG00000032000	Birc3
-1.15516	2.28115	8.88191969013504E-52	3.77215129240035E-50	ENSMUSG00000091971	Hspa1a
0.77641	4.31703	1.25414432670555E-51	5.26059353631454E-50	ENSMUSG00000039911	Spsb1
0.78107	5.22123	1.59484335298739E-51	6.67320169471669E-50	ENSMUSG00000007872	Id3
1.63312	-1.49112	2.19483691556365E-51	9.11635440625801E-50	ENSMUSG00000026574	Dpt
1.25208	1.22670	2.23982438946237E-51	9.28052115321629E-50	ENSMUSG00000026668	Ucma
1.64709	-0.52714	2.34077460480491E-51	9.6517182005888E-50	ENSMUSG00000032561	Acpp
0.82018	0.81981	3.12966239502707E-51	1.28421992190145E-49	ENSMUSG00000037469	Acp7
0.89858	3.26566	3.19698403023619E-51	1.30868348688319E-49	ENSMUSG00000023015	Racgap1
1.62191	0.90670	4.47787865584072E-51	1.82422548214441E-49	ENSMUSG00000034205	Loxl2
1.15425	1.76547	4.67943899531126E-51	1.90177774287913E-49	ENSMUSG00000046207	Pik3r6
0.75775	1.36634	4.80011416446357E-51	1.94153189109303E-49	ENSMUSG00000049580	Tsku
1.76702	0.05433	7.88487906736371E-51	3.13696312872072E-49	ENSMUSG00000059900	Tmem40
-0.71523	3.79117	8.17754541942848E-51	3.23823173858394E-49	ENSMUSG00000060862	Zbtb40
0.83025	2.50448	8.3842637083434E-51	3.31236911342646E-49	ENSMUSG00000015133	Lrrk1
0.82709	6.12772	1.21238972703331E-50	4.76761034325044E-49	ENSMUSG00000015243	Abca1
0.74323	0.97858	1.55685975878837E-50	6.10806780191611E-49	ENSMUSG00000025592	Dach2
0.81065	6.27139	1.792926380111E-50	7.01802611643447E-49	ENSMUSG00000030342	Cd9
0.90516	1.47632	2.07351763332487E-50	8.09768219653401E-49	ENSMUSG00000036718	Micall2
1.37796	0.16716	2.82640968024949E-50	1.09874250910934E-48	ENSMUSG00000074796	Slc4a11
1.16493	-0.11818	3.25182044500845E-50	1.25835821685202E-48	ENSMUSG00000070942	Il1rl2
1.82011	5.50258	3.95003084203908E-50	1.5216127878585E-48	ENSMUSG00000046805	Mpeg1
3.79794	-0.11502	5.06810659177302E-50	1.94789580952579E-48	ENSMUSG00000027656	Ccn5
1.06066	0.94612	5.18389760262148E-50	1.98790186170054E-48	ENSMUSG00000027115	Kif18a
0.93741	4.66599	5.7986463307371E-50	2.21863522221986E-48	ENSMUSG00000037206	Islr
1.11222	1.23589	1.22465986894489E-49	4.64386648518656E-48	ENSMUSG00000035258	Abi3bp
1.44791	-0.76228	1.27863451077067E-49	4.82698734866046E-48	ENSMUSG00000055632	Hmcn2
0.90091	5.39433	1.36681097335096E-49	5.13703203878012E-48	ENSMUSG00000033685	Ucp2
0.81780	3.09669	2.49894932780324E-49	9.35069409266991E-48	ENSMUSG00000004609	Cd33
0.72756	0.60846	3.1177142420163E-49	1.16148529700379E-47	ENSMUSG00000030598	Fbxo17
2.89446	-0.73620	3.15986535478609E-49	1.17461253057125E-47	ENSMUSG00000058260	Serpina9
1.16106	3.26088	3.21363386080787E-49	1.19199152898262E-47	ENSMUSG00000064201	Krt2
1.31362	0.01129	3.48373910648406E-49	1.28655999871633E-47	ENSMUSG00000035004	Igsf6
0.81851	8.42270	7.23938798837654E-49	2.65048972298579E-47	ENSMUSG00000024411	Aqp4
0.89233	1.48754	1.11602342810213E-48	4.06845622244613E-47	ENSMUSG00000031146	Plp2
1.34098	3.98724	1.11989055947858E-48	4.07381173970494E-47	ENSMUSG00000028364	Tnc

1.70546	-0.09320	1.1533649945265E-48	4.18661635192652E-47	ENSMUSG00000030713	Klk7
-0.96388	-1.01770	1.43903347732062E-48	5.20134057717504E-47	ENSMUSG00000095105	Edaradd
0.72698	2.28529	1.67603604642063E-48	6.04511684853367E-47	ENSMUSG00000033788	Dysf
2.13806	-0.77996	2.02336605986689E-48	7.25167565928663E-47	ENSMUSG00000027322	Siglec1
0.85478	5.13087	2.19590959351444E-48	7.8534972999207E-47	ENSMUSG00000004044	Cavin1
1.02463	-0.83429	2.63659957663413E-48	9.39005316726638E-47	ENSMUSG00000099032	Tcf24
1.24919	1.75717	3.75162371184435E-48	1.32500173839525E-46	ENSMUSG00000044811	Cd300c2
-1.73459	7.32268	3.8098490914792E-48	1.34277419846574E-46	ENSMUSG00000022602	Arc
0.90367	2.21824	5.36586268008557E-48	1.87562294669328E-46	ENSMUSG00000044456	Rin3
0.92304	2.44458	7.65442182761567E-48	2.67008866545247E-46	ENSMUSG00000056481	Cd248
1.52389	-1.12939	7.87276950106222E-48	2.7350226642954E-46	ENSMUSG00000090451	Gm6133
0.84216	2.22599	1.00421961993319E-47	3.47447716974035E-46	ENSMUSG00000036995	Asap3
3.02154	4.06618	1.11907143023558E-47	3.86398078391097E-46	ENSMUSG00000029304	Spp1
2.76274	-2.55902	1.22906719438038E-47	4.23517109495617E-46	ENSMUSG00000027483	Bpifa1
1.90598	0.68474	1.2540530465704E-47	4.31252088160686E-46	ENSMUSG00000015340	Cybb
0.99205	2.92184	1.30406283652365E-47	4.47543827613411E-46	ENSMUSG00000064080	Fbln2
0.75578	2.87319	1.35008567046294E-47	4.62404342133558E-46	ENSMUSG00000005413	Hmox1
0.84702	3.87151	1.45418463175814E-47	4.97056107128918E-46	ENSMUSG00000032359	Ctsh
6.62210	-1.60423	1.74066662243097E-47	5.92594079796738E-46	ENSMUSG00000058354	Krt6a
0.86886	4.89890	2.32017557422463E-47	7.86729394309942E-46	ENSMUSG00000018648	Dusp14
1.30566	-0.35634	2.34369320005822E-47	7.93120718776675E-46	ENSMUSG00000037705	Tecta
0.72682	5.55339	2.61211577635971E-47	8.82199260612302E-46	ENSMUSG00000022323	Rida
0.78968	5.14538	4.48467818151361E-47	1.49677235653346E-45	ENSMUSG00000026796	Fam129b
1.25162	2.94406	5.36750749892219E-47	1.78092221468145E-45	ENSMUSG00000020695	Mrc2
0.81779	3.67582	5.78568081009751E-47	1.9159287641703E-45	ENSMUSG00000050315	Synpo2
1.65042	2.01890	6.56474034025508E-47	2.1696849980594E-45	ENSMUSG00000013766	Ly6g6e
1.13793	0.82862	7.33573920511582E-47	2.41510731814937E-45	ENSMUSG00000078945	Naip2
0.85377	1.33268	7.40708271762757E-47	2.43387855332799E-45	ENSMUSG00000090066	1110002E22Rik
1.11411	1.96926	8.12123686010529E-47	2.66338941659206E-45	ENSMUSG00000021822	Plau
2.19691	1.84484	1.11673664200944E-46	3.63281805613229E-45	ENSMUSG00000020592	Sdc1
1.42244	-0.24964	1.11841525980527E-46	3.63281805613229E-45	ENSMUSG00000028031	Dkk2
1.52539	0.64742	1.27470383397646E-46	4.1168571733065E-45	ENSMUSG00000022468	Endou
1.18569	-0.33476	2.04496275708481E-46	6.53004272882644E-45	ENSMUSG00000031803	B3gnt3
2.31316	-1.51455	3.0625034244059E-46	9.7244501259453E-45	ENSMUSG00000001444	Tbx21
1.07260	0.67058	3.08001391655893E-46	9.76180530121326E-45	ENSMUSG00000029851	Tcaf2
0.79445	3.54321	3.20167784804174E-46	1.01285108533581E-44	ENSMUSG00000059602	Syn3
0.77207	2.34972	5.60272837496235E-46	1.74320786875202E-44	ENSMUSG00000031659	Adcy7
2.65436	3.83620	5.79807117481424E-46	1.79740206419242E-44	ENSMUSG00000024164	C3
0.95272	2.65813	9.10359626965138E-46	2.79659843451786E-44	ENSMUSG00000026380	Tfcp2l1
0.98401	4.53877	1.12841280028312E-45	3.44155774707534E-44	ENSMUSG00000048483	Zdhhc22
1.18693	3.18426	1.24303442445041E-45	3.77757939222962E-44	ENSMUSG00000021186	Fbln5
1.63156	-0.77318	1.71669878256965E-45	5.17079413444917E-44	ENSMUSG00000040528	Milr1
1.25465	-0.69786	2.10792154013495E-45	6.32674401480787E-44	ENSMUSG00000057457	Phex
1.42653	-0.16448	2.432565662539E-45	7.2754270202839E-44	ENSMUSG00000024011	Pi16
1.38888	1.89615	3.14793913214491E-45	9.39845166553212E-44	ENSMUSG00000030717	Nupr1
0.81194	7.49540	3.15419272399438E-45	9.40060105179236E-44	ENSMUSG00000009216	Fam163b
0.95517	2.47014	5.14850592827723E-45	1.5237424862295E-43	ENSMUSG00000051043	Gprc5c
1.41884	0.57561	7.87473776389747E-45	2.31847565221993E-43	ENSMUSG00000075217	4833423E24Rik
1.05002	2.51027	8.48056372425295E-45	2.4925227776403E-43	ENSMUSG00000022026	Olfm4
1.29571	3.64128	8.59691520437782E-45	2.52235570797876E-43	ENSMUSG00000040690	Col16a1
0.73055	3.33996	8.61622316186186E-45	2.52366205299499E-43	ENSMUSG00000026312	Cdh7

3.70777	-1.36803	8.92943910298535E-45	2.60641428662397E-43	ENSMUSG00000058952	Cfi
1.02050	2.10279	1.00991102918158E-44	2.93773434310559E-43	ENSMUSG00000004266	Ptpn6
0.89708	7.37206	1.17661278388444E-44	3.39937040350832E-43	ENSMUSG00000022548	Apod
-0.70571	2.03814	1.40146251007137E-44	4.03526188493093E-43	ENSMUSG00000029754	Dlx6
1.18201	-0.80763	1.44707828493516E-44	4.15955429855811E-43	ENSMUSG00000031383	Dusp9
0.90379	5.17813	1.49073257952499E-44	4.27058432731376E-43	ENSMUSG00000046182	Gsg1l
1.10018	2.17119	1.85323140773561E-44	5.28233140178063E-43	ENSMUSG00000024300	Myo1f
1.15286	-1.16329	1.87699587106188E-44	5.3411065088106E-43	ENSMUSG00000020081	Tacr2
1.17217	2.99545	2.1549734881399E-44	6.11163432663115E-43	ENSMUSG00000028859	Csf3r
0.76652	0.68760	2.57749729319669E-44	7.27350897289459E-43	ENSMUSG00000047363	Cstad
0.70833	1.82186	2.74560673025347E-44	7.72224621416323E-43	ENSMUSG00000026896	Ifih1
0.82542	1.54085	4.09797843863918E-44	1.14878643095054E-42	ENSMUSG00000004952	Rasa4
1.28470	1.60600	6.56529217156557E-44	1.8277352382391E-42	ENSMUSG00000096883	Shisa8
-1.30000	-0.89150	6.57373575797088E-44	1.8277352382391E-42	ENSMUSG00000042345	Ubash3a
1.64589	0.26689	7.13986657133137E-44	1.97866318619539E-42	ENSMUSG00000039013	Siglecf
1.19795	0.50641	8.4521895649344E-44	2.3271603943129E-42	ENSMUSG00000064267	Hvcn1
0.83211	2.12518	8.76731891564593E-44	2.41001963978953E-42	ENSMUSG00000059555	Tor4a
1.26875	1.52352	9.78622885267358E-44	2.67710878823219E-42	ENSMUSG00000024737	Slc15a3
0.85662	5.35277	1.10202048327308E-43	3.00499582180466E-42	ENSMUSG00000005142	Man2b1
1.02805	1.33473	1.16352807835064E-43	3.16763060817638E-42	ENSMUSG00000043008	Klhl6
1.42671	-0.23652	1.25490895946216E-43	3.4109429445349E-42	ENSMUSG00000000214	Th
-0.71186	9.01905	1.42345360618673E-43	3.85671927622013E-42	ENSMUSG00000026864	Hspa5
1.42226	4.04449	2.09282015653323E-43	5.66127847439275E-42	ENSMUSG00000030579	Tyrobp
1.37750	-0.53330	2.30310428191878E-43	6.21033897479939E-42	ENSMUSG00000037962	Rflna
0.85078	1.60482	2.63460424318453E-43	7.09297256469394E-42	ENSMUSG00000031004	Mki67
1.12540	-1.14324	3.10886164339533E-43	8.35654139208859E-42	ENSMUSG00000033182	Kbtbd12
2.20391	-1.04519	3.90864976295772E-43	1.04567153028545E-41	ENSMUSG00000040663	Clcf1
0.80271	4.39210	5.65121014932241E-43	1.50256174431195E-41	ENSMUSG00000027848	Olfml3
0.86585	2.29101	7.66977408216251E-43	2.03266961166578E-41	ENSMUSG00000030074	Gxylt2
0.74870	0.23427	1.09723476589674E-42	2.88542170325911E-41	ENSMUSG00000079445	B3gnt7
1.99788	4.91046	1.44130395227157E-42	3.78436963542341E-41	ENSMUSG00000041559	Fmod
1.05124	1.68024	1.45529254321747E-42	3.81520211792876E-41	ENSMUSG00000040258	Nxph4
0.72603	0.84747	1.48204458356282E-42	3.87934874970187E-41	ENSMUSG00000037139	Myom3
1.14495	1.71551	1.63919118174933E-42	4.28408919931655E-41	ENSMUSG00000000555	Itga5
1.25207	-1.43105	1.75442479038734E-42	4.5782132625346E-41	ENSMUSG00000045440	Insm2
-0.72078	4.89172	2.02849507653919E-42	5.26108005499967E-41	ENSMUSG00000055866	Per2
0.78446	7.01794	2.38756569188271E-42	6.18292164233285E-41	ENSMUSG00000053931	Cnn3
1.04792	1.45752	3.09543506808487E-42	7.9554086137104E-41	ENSMUSG00000024659	Anxa1
0.77595	-0.00282	3.70092948862633E-42	9.48286427643801E-41	ENSMUSG00000034035	Ccdc17
0.83936	0.85666	3.94035747386608E-42	1.0081143488861E-40	ENSMUSG00000022575	Gsdmd
0.85454	0.71176	4.27066510770334E-42	1.08607872529438E-40	ENSMUSG00000000901	Mmp11
0.93597	-0.10027	4.59628163999937E-42	1.16539750000462E-40	ENSMUSG00000038763	Alpk3
0.95368	3.50540	5.70446292292019E-42	1.43354165879539E-40	ENSMUSG00000036446	Lum
-1.33707	0.07502	6.02458325389071E-42	1.5117521464859E-40	ENSMUSG00000027356	Fermt1
0.91581	4.70868	6.61047920060497E-42	1.65388542945327E-40	ENSMUSG00000018459	Slc13a3
0.77910	6.07564	7.55313509375203E-42	1.87866264967291E-40	ENSMUSG00000023192	Grm2
1.18056	5.10703	7.99134283128727E-42	1.98185302215924E-40	ENSMUSG00000036896	C1qc
1.51119	1.97237	1.03520466102914E-41	2.55983359265838E-40	ENSMUSG00000028370	Pappa
1.03484	2.39039	1.17764755575502E-41	2.9036105482099E-40	ENSMUSG00000051617	Krt9
0.84538	0.00071	1.38967550292302E-41	3.41153286758039E-40	ENSMUSG00000020395	Itk
0.97586	0.74983	1.71055119464361E-41	4.19319533832693E-40	ENSMUSG00000055172	C1ra

1.06857	6.53285	1.93274961102307E-41	4.71746413679021E-40	ENSMUSG00000038642	Ctss
0.88614	1.94140	2.06246648653707E-41	5.02685518985535E-40	ENSMUSG00000022148	Fyb
0.91085	2.25557	2.15651668551477E-41	5.24104512925963E-40	ENSMUSG00000034164	Emid1
1.10888	5.61543	2.20179273625424E-41	5.34343642906958E-40	ENSMUSG00000029819	Npy
0.79413	-0.12313	2.34328446136998E-41	5.66254856753246E-40	ENSMUSG00000031145	Prickle3
1.24213	2.23299	2.35987897888675E-41	5.69454887689321E-40	ENSMUSG00000057751	Megf6
1.07459	-0.40477	2.41497187072454E-41	5.81922583544234E-40	ENSMUSG00000044626	Liph
0.77367	8.14500	3.14601937024259E-41	7.5274052199551E-40	ENSMUSG00000026185	Igfbp5
0.93834	1.81185	3.30908468034061E-41	7.90643186351987E-40	ENSMUSG00000090958	Lrrc32
-1.57886	-1.66443	4.16467325367168E-41	9.89503066201043E-40	ENSMUSG00000032769	Trpa1
0.96537	2.01700	4.4933318677224E-41	1.06461257697166E-39	ENSMUSG00000020399	Havcr2
2.34253	-1.53019	6.21406137124646E-41	1.46617325798243E-39	ENSMUSG00000063193	Cd300lb
4.05907	-1.68986	6.42821597880762E-41	1.5131664731854E-39	ENSMUSG00000049723	Mmp12
1.15738	-0.78381	6.43104658370532E-41	1.5131664731854E-39	ENSMUSG00000071203	Naip5
1.53179	0.29268	6.82893638877638E-41	1.60234767089134E-39	ENSMUSG00000038521	C1s1
0.80046	3.15879	7.4902106894679E-41	1.74785301088847E-39	ENSMUSG00000014329	Bicc1
2.48759	-3.15961	1.04068622224784E-40	2.4020621662427E-39	ENSMUSG00000109238	Gm35060
0.99573	-0.56798	1.10004801496173E-40	2.53219724636448E-39	ENSMUSG00000034762	Glis1
0.81516	1.82727	1.20969793108382E-40	2.77332637695707E-39	ENSMUSG00000031387	Renbp
2.16999	1.66326	1.22104802193675E-40	2.79557463566866E-39	ENSMUSG00000018927	Ccl6
-0.72313	4.55895	1.59629801927142E-40	3.63998802031984E-39	ENSMUSG00000046480	Scn4b
1.50292	-0.72077	2.72259883021447E-40	6.15865631527076E-39	ENSMUSG00000030674	Qprt
1.29462	1.17522	3.13423546098115E-40	7.07096839457473E-39	ENSMUSG00000078302	Foxd1
0.73025	6.40420	3.28795439158281E-40	7.40792695016033E-39	ENSMUSG00000017344	Vtn
1.79356	0.46912	4.20992882020267E-40	9.46008872984167E-39	ENSMUSG00000053175	Bcl3
1.26047	-0.10951	4.88684961304515E-40	1.09377867228473E-38	ENSMUSG00000026564	Dusp27
-0.77113	0.89501	5.83694970026728E-40	1.30471186194922E-38	ENSMUSG00000050783	Htr1f
2.02203	-1.28923	7.19242012440992E-40	1.59927791980989E-38	ENSMUSG00000022491	Glycam1
0.71658	1.12846	8.28495544213538E-40	1.83739977873363E-38	ENSMUSG00000070644	Etnk2
0.70248	1.28365	1.08007082248948E-39	2.38598740343969E-38	ENSMUSG00000002249	Tead3
1.53047	-0.49728	1.93372826778441E-39	4.24420876138522E-38	ENSMUSG00000041202	Pla2g2d
1.00552	2.33770	1.9663469819248E-39	4.31023258437915E-38	ENSMUSG00000031613	Hpgd
0.79396	2.05938	2.23421542942716E-39	4.89108913854492E-38	ENSMUSG00000017897	Eya2
0.77974	6.63329	2.259541766045E-39	4.93557923156454E-38	ENSMUSG00000028927	Padi2
0.81948	3.42642	3.06251505739935E-39	6.6022849993782E-38	ENSMUSG00000026840	Lamc3
1.92683	-1.09709	3.27727867696017E-39	7.05632574958167E-38	ENSMUSG00000028874	Fgr
0.99988	0.41864	3.96924757742018E-39	8.51383558651693E-38	ENSMUSG00000041482	Piezo2
0.95153	0.88670	5.39762374898337E-39	1.15339411632364E-37	ENSMUSG00000001918	Slc1a5
0.71075	2.62121	5.62442797322355E-39	1.19884294114331E-37	ENSMUSG00000029919	Hpgds
0.73425	0.85366	5.68266062630981E-39	1.20973732731518E-37	ENSMUSG00000034041	Lyl1
0.85899	1.11429	6.09061357467825E-39	1.29496049319943E-37	ENSMUSG00000091556	Gm14569
0.87097	3.69297	6.56632595968881E-39	1.39261854435947E-37	ENSMUSG00000027875	Hmgcs2
1.67111	-1.02732	6.94690617317987E-39	1.46966428480672E-37	ENSMUSG00000040264	Gbp2b
4.58602	-0.67422	7.0313188930443E-39	1.48567220590842E-37	ENSMUSG00000022986	Krt75
1.21279	-0.02671	7.4189043411352E-39	1.56173911954405E-37	ENSMUSG00000040751	Lat2
1.09649	1.38380	8.59455657285614E-39	1.80252255629235E-37	ENSMUSG00000024338	Psmb8
4.97884	-2.02977	1.06790470144743E-38	2.2177952406099E-37	ENSMUSG00000043613	Mmp3
1.10578	2.56798	1.13897516061436E-38	2.35962317420935E-37	ENSMUSG00000062980	Cped1
1.02601	2.67726	1.1578589893694E-38	2.39582320479993E-37	ENSMUSG00000041515	Irf8
1.11976	-0.27475	1.47170219638997E-38	3.02997757644633E-37	ENSMUSG00000037921	Ddx60
1.16937	5.24946	1.63579742514916E-38	3.35210213008853E-37	ENSMUSG00000036905	C1qb

1.94210	-1.67880	1.70906823237766E-38	3.49803025682309E-37	ENSMUSG00000006143	Upk3bl
1.09997	-0.46191	1.74752477569292E-38	3.57243693014095E-37	ENSMUSG00000009356	Lpo
1.24652	2.35506	1.77877387666346E-38	3.63194839143737E-37	ENSMUSG00000026479	Lamc2
1.36217	3.85528	2.04798254373599E-38	4.17660593673313E-37	ENSMUSG00000021702	Thbs4
0.81965	0.18034	2.16470702438498E-38	4.40935766549784E-37	ENSMUSG00000043953	Ccrl2
0.92867	-0.02216	2.4996004330509E-38	5.07326310115517E-37	ENSMUSG00000009545	Kcnq1
2.48639	-1.88132	2.55152893711475E-38	5.16631389555487E-37	ENSMUSG00000018930	Ccl4
1.18187	1.38252	3.06820303440655E-38	6.19769716391183E-37	ENSMUSG00000073530	Pappa2
0.77394	0.37872	3.45968002103957E-38	6.97189136386954E-37	ENSMUSG00000041479	Syt15
1.73600	-1.68297	3.65991385801914E-38	7.34983015430794E-37	ENSMUSG00000061577	Adgrg5
1.37773	-0.60780	3.69739502908072E-38	7.41574341842069E-37	ENSMUSG00000045394	Epcam
0.70581	5.40338	4.08141965816086E-38	8.17631570198546E-37	ENSMUSG00000010066	Cacna2d2
0.88795	0.79448	4.22982530388466E-38	8.46363630888016E-37	ENSMUSG00000045322	Tlr9
1.30360	0.23196	4.50029451097552E-38	8.99423566499437E-37	ENSMUSG00000015355	Cd48
0.86788	-0.36704	4.79183219086042E-38	9.54321749804653E-37	ENSMUSG00000033114	Slc35d2
0.90598	2.32939	5.10568334750781E-38	1.0144485228943E-36	ENSMUSG00000029659	Medag
0.98143	-0.27681	6.03924067382852E-38	1.19853528699765E-36	ENSMUSG00000010830	Kdelr3
1.12643	4.56433	6.60788279349081E-38	1.30985662655568E-36	ENSMUSG00000001930	Vwf
-1.02477	5.78613	7.07038698980874E-38	1.39664807189385E-36	ENSMUSG00000052837	Junb
0.77219	0.42970	1.22884233018956E-37	2.41336109887401E-36	ENSMUSG00000022754	Tmem45a
0.73884	1.21381	1.31550822137944E-37	2.57760711243298E-36	ENSMUSG00000073002	Vamp5
0.78280	2.04621	1.95242568267417E-37	3.79929066406286E-36	ENSMUSG00000060591	Ifitm2
0.77244	3.88475	2.00056671946617E-37	3.88851572428963E-36	ENSMUSG00000052942	Glis3
1.24648	-0.83829	2.0470691352514E-37	3.96981854676379E-36	ENSMUSG00000028640	Tfap2c
0.81807	5.05157	2.24867965922611E-37	4.34097386942422E-36	ENSMUSG00000024899	Papss2
0.78815	-0.21915	2.47923937207298E-37	4.76440253990677E-36	ENSMUSG00000024727	Trpm6
2.21590	-1.50665	2.53462743629737E-37	4.85196175328099E-36	ENSMUSG00000059089	Fcgr4
-0.90879	2.94595	2.79796230851355E-37	5.34665733374894E-36	ENSMUSG00000074934	Grem1
0.96424	-0.30250	3.07967694265322E-37	5.8520751568001E-36	ENSMUSG00000043832	Clec4a3
0.71414	3.56725	3.3098778623658E-37	6.27546932208374E-36	ENSMUSG00000008540	Mgst1
1.01503	3.98589	4.48140497328881E-37	8.42147208918478E-36	ENSMUSG00000036103	Colec12
0.81061	0.17891	7.09459728218324E-37	1.31863258894671E-35	ENSMUSG00000020227	Irak3
0.96949	2.91425	7.29697289718035E-37	1.35476476040765E-35	ENSMUSG00000000290	Itgb2
0.92084	4.74403	8.19291332816258E-37	1.51944554169024E-35	ENSMUSG00000003617	Cp
1.09199	2.53839	8.88596083804595E-37	1.6443867398336E-35	ENSMUSG00000026712	Mrc1
1.24393	-2.05842	1.14879884097447E-36	2.11438729257576E-35	ENSMUSG00000063767	S100a7a
0.85044	1.59820	1.42981730946291E-36	2.61461102832678E-35	ENSMUSG00000020914	Top2a
0.70234	4.68007	1.68699736519757E-36	3.07827188399316E-35	ENSMUSG00000024998	Plce1
0.85816	4.72576	1.96100326569267E-36	3.56958870383224E-35	ENSMUSG00000031980	Agt
-0.79234	2.83904	2.36366929217967E-36	4.29454694497841E-35	ENSMUSG00000055148	Klf2
1.47787	4.11211	2.46567310897026E-36	4.47509132213535E-35	ENSMUSG00000023992	Trem2
0.94028	4.26245	3.11236373704538E-36	5.60687541515661E-35	ENSMUSG00000039405	Prss23
0.91087	2.08295	3.17457313793378E-36	5.70684110764224E-35	ENSMUSG00000041736	Tspo
-0.90236	-1.29016	3.17852690726548E-36	5.70790857300486E-35	ENSMUSG00000056043	Rgs9bp
1.06041	-0.26476	4.44987590302407E-36	7.95731493058661E-35	ENSMUSG00000028755	Cda
0.92934	0.96128	4.69128288927811E-36	8.35502558381448E-35	ENSMUSG00000056529	Ptafr
1.74813	-0.82702	4.6985125147006E-36	8.35794037693548E-35	ENSMUSG00000030730	Atp2a1
0.82766	2.21947	7.25900114756127E-36	1.28187018185832E-34	ENSMUSG00000028268	Gbp3
2.24417	-2.58975	7.7499366552082E-36	1.36572535164603E-34	ENSMUSG00000048521	Cxcr6
1.75778	-0.63341	8.27394538949774E-36	1.45655735001852E-34	ENSMUSG00000032717	Mdfi
0.91742	2.59254	8.35657912576419E-36	1.46812812977947E-34	ENSMUSG00000030748	Il4ra

0.78196	2.93622	8.35695727276166E-36	1.46812812977947E-34	ENSMUSG00000070366	Plpp4
1.17938	5.99430	8.41769432805891E-36	1.47727057071348E-34	ENSMUSG00000036887	C1qa
0.96549	5.24305	8.89220601322298E-36	1.55732779126425E-34	ENSMUSG00000036570	Fxyd1
-1.43307	-0.60797	9.45624356625301E-36	1.65208420029202E-34	ENSMUSG00000016942	Tmprss6
-0.95517	3.62038	1.12135214351545E-35	1.94184813598781E-34	ENSMUSG00000090877	Hspa1b
1.60273	-1.92178	1.19499208412944E-35	2.06726329177096E-34	ENSMUSG00000012705	Retn
1.02081	1.48289	1.35844386727309E-35	2.34763422352343E-34	ENSMUSG00000032419	Tbx18
1.12068	-0.57751	1.3820305982685E-35	2.38596908570989E-34	ENSMUSG00000013611	Snx31
0.76217	2.68928	2.22650833610928E-35	3.81674304882184E-34	ENSMUSG00000027230	Creb3l1
1.21927	1.24464	2.37079694196685E-35	4.05182076963108E-34	ENSMUSG00000071713	Csf2rb
1.94990	-1.51720	2.87622506800634E-35	4.89592299151219E-34	ENSMUSG00000038204	Asb10
-0.73079	4.86896	3.19578238754576E-35	5.41815880235803E-34	ENSMUSG00000030222	Rerg
2.06095	-0.75628	3.32324212705943E-35	5.62863781201252E-34	ENSMUSG00000025044	Msr1
2.54679	-0.57555	3.68733968467537E-35	6.22050909267777E-34	ENSMUSG00000000982	Ccl3
0.91112	4.31722	4.15179171565341E-35	6.99708706999208E-34	ENSMUSG00000020120	Plek
0.99198	2.82851	4.22384899960063E-35	7.1114714375833E-34	ENSMUSG00000006519	Cyba
0.87622	0.38558	4.25289230826716E-35	7.15328064681609E-34	ENSMUSG00000074342	I830077J02Rik
0.96976	5.50050	5.87330076660379E-35	9.79152437910355E-34	ENSMUSG00000051111	Sv2c
1.10629	-1.07433	7.60825223845186E-35	1.25362743963938E-33	ENSMUSG00000027376	Prom2
0.77862	4.38186	7.87895703381498E-35	1.29571851007211E-33	ENSMUSG00000036181	Hist1h1c
1.95407	1.96464	9.58978215776651E-35	1.56914361170766E-33	ENSMUSG00000073421	H2-Ab1
0.95957	5.44372	9.59701090513455E-35	1.56914361170766E-33	ENSMUSG00000016256	Ctsz
0.86683	1.09716	1.00421203536285E-34	1.63876599968724E-33	ENSMUSG00000047878	A4galt
1.03792	0.30463	1.13683280934088E-34	1.84455737966407E-33	ENSMUSG00000034947	Tmem106a
1.37017	-2.26458	1.35682848951226E-34	2.19521927426993E-33	ENSMUSG00000034990	Otoa
0.80482	1.97853	1.43800440339769E-34	2.32213106510647E-33	ENSMUSG00000034422	Parp14
0.82010	0.45128	1.52106843095852E-34	2.45160441225079E-33	ENSMUSG00000026922	Agpat2
1.82233	1.01468	1.70743559700858E-34	2.74417369176743E-33	ENSMUSG00000048373	Fgfbp1
0.82102	-0.05088	2.1719145190548E-34	3.48408723793229E-33	ENSMUSG00000017453	Pipox
0.72724	1.84893	2.36978262812587E-34	3.79433244925564E-33	ENSMUSG00000017446	C1qtnf1
0.93902	0.00038	2.41937015548732E-34	3.87008099825035E-33	ENSMUSG00000047180	Neurl3
1.49024	-2.20327	2.43305094836655E-34	3.88830381099256E-33	ENSMUSG00000045326	Fndc7
-0.76487	3.99236	2.55420737731349E-34	4.07808974866556E-33	ENSMUSG00000034765	Dusp5
1.04104	-0.93878	2.692862545906E-34	4.28738040579673E-33	ENSMUSG00000009246	Trpm5
1.26108	-0.86514	3.33707229465064E-34	5.29814805060981E-33	ENSMUSG00000024672	Ms4a7
1.99814	-1.38983	4.22678175266428E-34	6.67328702734766E-33	ENSMUSG00000034774	Dsg1c
0.86609	0.37814	4.29262868582816E-34	6.75840371778024E-33	ENSMUSG00000022759	Lrrc74b
-0.76940	2.26007	4.57291048951474E-34	7.18636479147792E-33	ENSMUSG00000049107	Ntf3
0.73813	2.46283	5.00514083788672E-34	7.8294044709042E-33	ENSMUSG00000019359	Gdpd2
1.49540	-1.44038	5.23931623223991E-34	8.18817885494862E-33	ENSMUSG00000096035	Odaph
1.00026	-0.44361	5.40045290703964E-34	8.43225128536668E-33	ENSMUSG00000026829	Gbgt1
0.84709	0.69680	5.65904030138229E-34	8.81979602200756E-33	ENSMUSG00000031103	Elf4
1.28166	-1.24943	6.13743589510854E-34	9.55662337177854E-33	ENSMUSG00000032494	Tdgf1
0.80592	1.44736	6.34068692036664E-34	9.84603193813423E-33	ENSMUSG00000090523	Gypc
1.08347	3.42519	6.43953779083641E-34	9.99039890326292E-33	ENSMUSG00000001435	Col18a1
1.55143	-1.80451	6.82824471379638E-34	1.05741313762965E-32	ENSMUSG00000015314	Slamf6
0.78564	-0.05885	8.03413536902325E-34	1.24189164375766E-32	ENSMUSG00000046610	Oacyl
1.30911	-0.96438	9.12982520832365E-34	1.40486839346922E-32	ENSMUSG00000039981	Zc3h12d
4.81593	-2.60748	1.16552078909393E-33	1.77418164562076E-32	ENSMUSG00000063130	Calml3
0.81354	-0.57054	1.18167578468136E-33	1.79555529786823E-32	ENSMUSG00000005131	4930550C14Rik
1.41028	-1.74167	1.24321432984738E-33	1.88737489146089E-32	ENSMUSG00000018862	Otop3

-0.73087	3.06252	1.61534005795728E-33	2.43923528040695E-32	ENSMUSG00000036915	Kirrel2
2.52898	-2.61192	1.70271962912727E-33	2.56661943741031E-32	ENSMUSG00000026981	Il1rn
-1.12473	-0.87342	1.77043629731919E-33	2.66632728890589E-32	ENSMUSG00000007877	Tcap
0.97031	0.01327	1.77494012474351E-33	2.67074250125268E-32	ENSMUSG00000051079	Rgs13
1.26000	0.71923	2.19596679061105E-33	3.28968993288365E-32	ENSMUSG00000004098	Col5a3
1.45157	-0.94713	3.00425322013999E-33	4.46511405981962E-32	ENSMUSG00000009670	Tex11
1.20957	-1.09556	3.11758174579563E-33	4.62142047971868E-32	ENSMUSG00000056498	Tmem154
0.76446	1.12866	3.18895057488367E-33	4.72309436496284E-32	ENSMUSG00000032596	Uba7
1.92910	1.22640	3.65049600602996E-33	5.37854519951752E-32	ENSMUSG00000037411	Serpine1
0.89392	1.50023	3.71820646491986E-33	5.47227601616946E-32	ENSMUSG00000030921	Trim30a
0.85574	0.85812	3.72055497920634E-33	5.47227601616946E-32	ENSMUSG00000034652	Cd300a
1.40094	-1.50746	3.99828114735163E-33	5.86046593021652E-32	ENSMUSG00000002020	Ltbp2
1.23489	-1.83388	4.34295613630187E-33	6.34377806048978E-32	ENSMUSG00000090084	Srpx
0.72260	0.74870	4.36341447405359E-33	6.36818600388509E-32	ENSMUSG00000038352	Arl5c
1.24198	-1.40218	4.58513580992417E-33	6.67457473341833E-32	ENSMUSG00000039883	Lrrc17
0.83675	0.18348	4.65674461053811E-33	6.76721791649456E-32	ENSMUSG00000027186	Elf5
0.84646	3.99775	5.58759307397729E-33	8.08535188592216E-32	ENSMUSG00000006369	Fbln1
0.86116	1.75269	5.86303974073736E-33	8.46230408799034E-32	ENSMUSG00000052688	Rab7b
0.83578	2.16506	6.46546333278479E-33	9.3080755167244E-32	ENSMUSG00000039062	Anpep
2.42743	-3.27819	6.64802198894545E-33	9.56279403456438E-32	ENSMUSG00000031936	Hephl1
1.29634	-1.48114	6.65511822898002E-33	9.56490257816519E-32	ENSMUSG00000052631	Sh2d6
1.34086	-0.92463	7.74349505053075E-33	1.10729371985199E-31	ENSMUSG00000062585	Cnr2
3.64136	-3.52635	8.16025137619553E-33	1.16297273807726E-31	ENSMUSG00000027762	Sucnr1
0.72884	1.53314	8.53092717213777E-33	1.21478114669134E-31	ENSMUSG00000051506	Wdfy4
2.06915	-0.12815	9.09616793200836E-33	1.2931020989871E-31	ENSMUSG00000071178	Serpina1b
1.23071	-1.08069	9.37871952372201E-33	1.33215457582767E-31	ENSMUSG00000026069	Il1rl1
0.96354	2.14066	1.08522628913652E-32	1.53888348913616E-31	ENSMUSG00000020773	Trim47
0.98684	2.91443	1.14350958900179E-32	1.61882840816354E-31	ENSMUSG00000058715	Fcer1g
1.55013	-2.00003	1.4856544203152E-32	2.09099397616525E-31	ENSMUSG00000026012	Cd28
1.86010	-0.04545	1.73423481145956E-32	2.43079050965965E-31	ENSMUSG00000034855	Cxcl10
1.06738	-1.30901	1.82493464520136E-32	2.55370591043499E-31	ENSMUSG00000029660	Tex26
0.80131	-0.62577	2.07674903871069E-32	2.89653634397514E-31	ENSMUSG00000028068	Iqgap3
0.72527	2.39840	2.10048164053907E-32	2.92723397124509E-31	ENSMUSG00000028073	Pear1
1.43305	3.64427	2.35164301750483E-32	3.2638653252755E-31	ENSMUSG00000030108	Slc6a13
1.64337	-2.30511	3.20264808356939E-32	4.43411455938687E-31	ENSMUSG00000006546	Cryba2
0.79085	0.03869	3.35730118685072E-32	4.63689695627806E-31	ENSMUSG00000031880	Rrad
0.85948	0.87006	4.50865288145878E-32	6.20688777554471E-31	ENSMUSG00000042485	Mustn1
1.03862	2.66269	4.77708728300001E-32	6.57110597276147E-31	ENSMUSG00000030208	Emp1
0.70080	-0.10892	5.66362441917778E-32	7.76542789612527E-31	ENSMUSG00000006398	Cdc20
1.36415	1.68812	6.09639195841603E-32	8.33860761590753E-31	ENSMUSG00000027995	Tlr2
-0.75705	3.40411	6.87680539822332E-32	9.39093007275062E-31	ENSMUSG00000062563	Cys1
0.70642	0.62671	7.08436474945891E-32	9.65884336788186E-31	ENSMUSG00000019088	Dnase1l1
1.39188	-2.40267	7.11578902054617E-32	9.69390728797421E-31	ENSMUSG00000058831	Opn1sw
0.83054	0.42181	8.56273502922023E-32	1.16092372447241E-30	ENSMUSG00000032322	Pstpip1
0.99008	1.77741	1.27761049489167E-31	1.71940684291718E-30	ENSMUSG00000062488	Ifit3b
1.61950	-1.17253	1.27832048658135E-31	1.71940684291718E-30	ENSMUSG00000000216	Scnn1g
1.11164	0.53086	1.29618640533155E-31	1.74205812134275E-30	ENSMUSG00000032179	Bmp5
0.74886	2.71539	1.37998842216043E-31	1.85175697596061E-30	ENSMUSG00000009687	Fxyd5
1.60587	-2.29693	1.39733745288034E-31	1.87355711519583E-30	ENSMUSG00000033213	AA467197
1.51141	0.44009	1.47516384218674E-31	1.97634726743441E-30	ENSMUSG00000021070	Bdkrb2
-0.87296	-1.28854	1.54241274030067E-31	2.05994556857137E-30	ENSMUSG00000046080	Clec9a

1.04735	0.57671	1.73563489044853E-31	2.30892447290052E-30	ENSMUSG00000059479	B3gnt8
0.86917	0.10765	1.99956674477754E-31	2.64759468903203E-30	ENSMUSG00000039063	Echdc3
0.73883	2.54328	2.15457428369093E-31	2.84397109023633E-30	ENSMUSG00000031283	Chrdl1
0.93896	-0.81658	2.38867112589825E-31	3.14320256287834E-30	ENSMUSG00000033581	Igf2bp2
1.13120	0.24946	2.87855548627053E-31	3.77613132052229E-30	ENSMUSG00000021281	Tnfaip2
1.00888	0.73694	3.12975909106738E-31	4.08912053442771E-30	ENSMUSG00000022952	Runx1
0.95882	-0.26049	3.13159042576551E-31	4.08912053442771E-30	ENSMUSG00000038217	Tlcd2
0.72571	6.38488	3.54065691085906E-31	4.61262880380933E-30	ENSMUSG00000027737	Slc7a11
0.86557	0.84337	4.22243984367981E-31	5.47145751826336E-30	ENSMUSG00000022686	B3gnt5
0.92095	-0.10650	4.54462430168406E-31	5.88445713696713E-30	ENSMUSG00000040345	Arhgap9
1.10336	0.64919	4.84292801257723E-31	6.25640008195148E-30	ENSMUSG00000058806	Col13a1
-1.91184	-2.87537	4.85051257107708E-31	6.26143674448765E-30	ENSMUSG00000009900	Wnt3a
0.92964	1.97164	4.91976071487547E-31	6.34600569660626E-30	ENSMUSG00000029561	Oasl2
1.08630	4.34009	5.81780160156112E-31	7.46471401867978E-30	ENSMUSG00000039004	Bmp6
1.37353	-1.11020	5.89871916540903E-31	7.56282574958254E-30	ENSMUSG00000025401	Myo1a
0.73791	-0.03029	6.40550173022615E-31	8.19402585791279E-30	ENSMUSG00000035298	Klhl35
1.25388	3.60520	7.72184196224304E-31	9.87047789725995E-30	ENSMUSG00000029718	Pcolce
0.80717	4.12823	7.93978339933125E-31	1.01338122004387E-29	ENSMUSG00000027800	Tm4sf1
2.60638	-2.81729	9.8312607428475E-31	1.24823211883029E-29	ENSMUSG00000025329	Padi1
0.81410	1.59513	9.9703099052647E-31	1.26494118499355E-29	ENSMUSG00000020865	Abcc3
1.38651	-0.12811	1.02213838469919E-30	1.29293275348249E-29	ENSMUSG00000041538	H2-Ob
4.69105	-3.24905	1.08675854911686E-30	1.37160878398196E-29	ENSMUSG00000053719	Klk1b26
0.99599	2.12711	1.15896338913744E-30	1.46056899515333E-29	ENSMUSG00000032372	Plscr2
0.72848	-0.21466	1.16060946427343E-30	1.46155919785597E-29	ENSMUSG00000038151	Prdm1
1.29665	2.63174	1.17843072479675E-30	1.48070866515141E-29	ENSMUSG00000037254	Itih2
-0.83810	4.04180	1.26222541623785E-30	1.58482523067617E-29	ENSMUSG00000022769	Sdf2l1
0.79690	-0.71699	1.29297881035352E-30	1.62223958864738E-29	ENSMUSG00000067714	Lpar5
-0.70326	-0.06143	1.34234480030871E-30	1.68045346113813E-29	ENSMUSG00000072423	Psmb11
0.86189	2.08081	1.38283460159157E-30	1.72986702590851E-29	ENSMUSG00000032796	Lama1
4.75961	-1.87488	1.46479620714926E-30	1.82567556618133E-29	ENSMUSG00000045545	Krt14
0.94128	0.86678	1.4805738545312E-30	1.84398743700703E-29	ENSMUSG00000024778	Fas
0.73596	0.12039	1.51122139943527E-30	1.88077869110669E-29	ENSMUSG00000044505	Lingo4
0.70169	2.08329	1.72021925083481E-30	2.13462999511919E-29	ENSMUSG00000042190	Cmklr1
-0.70112	1.75935	1.85333456361292E-30	2.29813485888002E-29	ENSMUSG00000019935	Slc17a8
0.98805	-0.44747	2.09442995244735E-30	2.59141850197929E-29	ENSMUSG00000059645	Gm7361
0.79207	2.09717	2.29750235341162E-30	2.8364803764358E-29	ENSMUSG00000020080	Hkdc1
1.60581	1.56167	2.73288981625227E-30	3.36179089054986E-29	ENSMUSG00000053113	Socs3
0.86206	0.76851	2.79026035127198E-30	3.4298800902611E-29	ENSMUSG00000011171	Vipr2
3.05082	-3.64471	3.45416282258483E-30	4.22153367122814E-29	ENSMUSG00000075219	Olfr994
1.39178	-2.10926	3.4887203188849E-30	4.25458584186767E-29	ENSMUSG00000030838	Ush1c
1.10757	1.36176	3.56154661127395E-30	4.34028363216083E-29	ENSMUSG00000079022	Col22a1
2.36530	-1.64617	3.63974226340122E-30	4.42922217554871E-29	ENSMUSG00000034185	6430628N08Rik
1.53247	-2.18228	3.67026298992892E-30	4.45997336716112E-29	ENSMUSG00000030523	Trpm1
1.39736	-1.69562	3.76032482676731E-30	4.56288558265165E-29	ENSMUSG00000007030	Vwa7
0.77218	-0.47822	4.5809602595353E-30	5.52316202192944E-29	ENSMUSG00000054580	Pla2r1
1.46635	3.71124	5.02401803603202E-30	6.03166207746375E-29	ENSMUSG00000013584	Aldh1a2
0.81650	-0.45523	5.27853467711042E-30	6.32828137577642E-29	ENSMUSG00000037661	Gpr160
0.75157	-0.05548	5.53959343210021E-30	6.63657357013529E-29	ENSMUSG00000037725	Ckap2
0.81154	0.19940	6.17891608042968E-30	7.37130803190586E-29	ENSMUSG00000026875	Traf1
0.79001	2.94132	6.35849802264939E-30	7.56959806648688E-29	ENSMUSG00000050558	Prokr2
1.12160	3.41230	8.51688107256254E-30	1.00405812394651E-28	ENSMUSG00000029675	Eln

2.81448	-2.78161	8.72643199933205E-30	1.0266248393674E-28	ENSMUSG00000055368	Slc6a2
1.14544	2.56887	8.80601903944853E-30	1.03527094423634E-28	ENSMUSG00000074896	Ifit3
1.01991	3.57552	9.40784197023859E-30	1.1029704581809E-28	ENSMUSG00000021423	Ly86
3.50669	-3.51475	1.10087740160282E-29	1.28710979342248E-28	ENSMUSG00000075218	Olfr995
2.77083	-2.25480	1.12560153000974E-29	1.31511133368676E-28	ENSMUSG00000114755	Galr3
3.63003	-0.24855	1.26878085357495E-29	1.47529426013219E-28	ENSMUSG00000023078	Cxcl13
1.26349	-0.58017	1.44973172472569E-29	1.67994833148977E-28	ENSMUSG00000031340	Gabre
1.73202	0.70818	1.48921044067289E-29	1.72451990225978E-28	ENSMUSG00000020884	Asgr1
1.01507	2.57514	1.49268789168409E-29	1.72736933950472E-28	ENSMUSG00000035095	Fam167a
2.94875	-3.43734	1.57705790136373E-29	1.82128209574216E-28	ENSMUSG00000031725	Ces1f
4.46992	-3.17067	1.66098236828256E-29	1.91559867429628E-28	ENSMUSG00000063089	Klk1b8
1.34682	-1.81279	1.7073865959924E-29	1.96778042691445E-28	ENSMUSG00000093942	Olfr46
0.71785	2.07297	1.71752715568775E-29	1.97812551327616E-28	ENSMUSG00000002111	Spi1
-0.77679	2.32820	1.75655783271906E-29	2.02170761939238E-28	ENSMUSG00000014813	Stc1
1.69118	-1.03592	1.95544513333617E-29	2.23999338672386E-28	ENSMUSG00000044254	Pcsk9
1.30385	0.41995	2.02445217046208E-29	2.31591875231043E-28	ENSMUSG00000044055	Otos
0.70357	3.97534	2.17617872970161E-29	2.47947178136626E-28	ENSMUSG00000022150	Dab2
-0.75957	7.68533	2.18212725046951E-29	2.48458295784022E-28	ENSMUSG00000007617	Homer1
1.36091	1.60495	2.18733464793522E-29	2.48884400529963E-28	ENSMUSG00000061878	Sphk1
1.43519	-0.87536	2.3493422525418E-29	2.67139398836547E-28	ENSMUSG00000079419	Ms4a6c
0.79557	0.92757	2.6994970410917E-29	3.05319944967149E-28	ENSMUSG00000045259	Klhdc9
0.74633	3.60788	2.79329221674835E-29	3.15298658990838E-28	ENSMUSG00000028763	Hspg2
1.32320	-0.05794	2.86133988359146E-29	3.22765218741379E-28	ENSMUSG00000071489	Ptgdr
4.76656	-1.94381	3.96753355894762E-29	4.45181374500675E-28	ENSMUSG00000028238	Atp6v0d2
-1.26060	-0.39637	5.17332356081817E-29	5.77806841888094E-28	ENSMUSG00000002565	Scin
0.94706	-1.57758	5.24283190547524E-29	5.85185469186685E-28	ENSMUSG00000057228	Aadat
0.78723	1.10568	5.54068789621986E-29	6.17214465449069E-28	ENSMUSG00000028600	Podn
1.98327	-2.97025	6.43284015170904E-29	7.12393014975444E-28	ENSMUSG00000029188	Slc34a2
0.71498	0.10378	7.41216956451739E-29	8.18180224574538E-28	ENSMUSG00000048327	Ckap2l
0.88151	-0.04699	1.06764936508247E-28	1.16638118418141E-27	ENSMUSG00000022033	Pbk
1.11254	4.42734	1.20553627762884E-28	1.3127980951512E-27	ENSMUSG00000061232	H2-K1
0.89306	-1.42908	1.41172057524436E-28	1.53143736476699E-27	ENSMUSG00000071724	Smpd5
1.24089	-2.27784	1.46694746072726E-28	1.58931782288486E-27	ENSMUSG00000022871	Fetub
0.80268	3.57368	1.49783079826296E-28	1.62174312306508E-27	ENSMUSG00000059498	Fcgr3
0.70841	-0.03521	1.68484930733344E-28	1.81728381161781E-27	ENSMUSG00000031861	Lpar2
0.88524	-0.65468	1.7772543003173E-28	1.91573579021511E-27	ENSMUSG00000026726	Cubn
1.10240	-1.37601	2.09167089185678E-28	2.24184890289357E-27	ENSMUSG00000029530	Ccr9
0.95891	1.35360	2.18319877812851E-28	2.33847294091092E-27	ENSMUSG00000029925	Tbxas1
0.87013	8.23133	2.46825273945943E-28	2.63714953068785E-27	ENSMUSG00000007891	Ctsd
1.81246	-1.25257	2.52717123948669E-28	2.69670760153266E-27	ENSMUSG00000089929	Bcl2a1b
0.77388	1.35981	2.62333449161398E-28	2.79580968278158E-27	ENSMUSG00000052142	Rasal3
-0.85459	1.13997	2.66361545840879E-28	2.83695921049834E-27	ENSMUSG00000048562	Sp8
0.70840	1.70478	2.77118940372624E-28	2.9459928404569E-27	ENSMUSG00000033220	Rac2
0.82760	-0.58301	3.00051887080893E-28	3.18381102918814E-27	ENSMUSG00000034457	Eda2r
1.44958	-1.78362	3.05025655752061E-28	3.23254887081473E-27	ENSMUSG00000022705	Drd3
3.07923	-3.70849	3.34844106195881E-28	3.54413188539292E-27	ENSMUSG00000070821	Olfr1340
0.89626	0.97035	3.48149580365033E-28	3.68037652224094E-27	ENSMUSG00000048096	Lmod1
2.22813	-2.16514	3.72807380124759E-28	3.92881623669938E-27	ENSMUSG00000112148	Lilrb4a
0.75811	-0.07220	3.8761209610965E-28	4.07472418855862E-27	ENSMUSG00000049988	Lrrc25
0.70199	3.97019	3.9376904108261E-28	4.13688835492355E-27	ENSMUSG00000064246	Chil1
0.71189	-1.01492	4.10254853388705E-28	4.29945061651284E-27	ENSMUSG00000091648	C2cd4d

0.73585	1.43964	4.11072811498718E-28	4.30536678282381E-27	ENSMUSG00000030157	Clec2d
1.93128	3.18854	4.25909583889967E-28	4.4552660167012E-27	ENSMUSG00000024610	Cd74
1.54665	-2.44684	4.70629716389672E-28	4.90494332639739E-27	ENSMUSG00000027068	Dhrs9
2.29333	-3.15033	4.77269720340763E-28	4.96805025070397E-27	ENSMUSG00000030162	Olr1
1.22843	-1.29959	5.01109669355804E-28	5.20663673578984E-27	ENSMUSG00000033538	Casp4
0.97353	-0.00549	5.12929482275459E-28	5.32156435626005E-27	ENSMUSG00000069793	Slfn9
1.58591	-2.55187	5.13110573084174E-28	5.32156435626005E-27	ENSMUSG00000046846	Spesp1
0.70755	3.11773	5.5968521135956E-28	5.7904582037614E-27	ENSMUSG00000055541	Lair1
1.26891	-0.31625	5.91063910088526E-28	6.11137778733042E-27	ENSMUSG00000036523	Greb1
0.88863	2.40110	5.95087076952249E-28	6.14923312850658E-27	ENSMUSG00000074001	Klhl40
0.98227	2.07331	8.05464088772217E-28	8.26281638892658E-27	ENSMUSG00000045672	Col27a1
0.73896	0.66405	9.01041466308826E-28	9.21546804915974E-27	ENSMUSG00000031264	Btk
0.87723	-1.21737	9.36750994230265E-28	9.56916770293671E-27	ENSMUSG00000022945	Chaf1b
1.80661	-0.84418	9.50602345030723E-28	9.69899858101017E-27	ENSMUSG00000079507	H2-Q1
1.12870	-1.28452	1.06669094140933E-27	1.08638763265358E-26	ENSMUSG00000027485	Bpifb1
1.13860	-0.84989	1.06784401659945E-27	1.08691037471488E-26	ENSMUSG00000039238	Zfp750
1.02927	-0.63803	1.13788118081116E-27	1.15542890015661E-26	ENSMUSG00000037482	Erv3
0.74985	-1.18394	1.31401790520955E-27	1.32872239129166E-26	ENSMUSG00000031712	Il15
0.71239	1.57471	1.45188491521931E-27	1.46551520735268E-26	ENSMUSG00000049625	Tifab
1.64573	-2.79926	1.46190504628761E-27	1.47387791847679E-26	ENSMUSG00000096069	Olfr61
0.91573	4.11486	1.53078819392407E-27	1.54240983620297E-26	ENSMUSG00000033880	Lgals3bp
1.37254	-1.64425	1.53514197020383E-27	1.5458797741448E-26	ENSMUSG00000091649	Phf11b
2.41294	-1.60969	1.61702744391697E-27	1.6264098411641E-26	ENSMUSG00000073555	Gm4951
1.21749	-1.32871	1.71649068670768E-27	1.72338911263534E-26	ENSMUSG00000057191	AB124611
0.79362	-0.31795	1.9141209338746E-27	1.91954465316775E-26	ENSMUSG00000042349	Ikbke
1.05392	0.16645	2.07892649524343E-27	2.07990596591257E-26	ENSMUSG00000054966	Lmntd1
0.73729	1.50622	2.12445079808927E-27	2.12093939842875E-26	ENSMUSG00000024014	Pim1
0.74424	2.71009	2.21513160192751E-27	2.20837181065403E-26	ENSMUSG00000027932	Slc27a3
0.91534	5.66999	2.36483446991269E-27	2.35072018577394E-26	ENSMUSG00000038400	Pmepa1
0.90415	0.90470	2.54371117357761E-27	2.52262495135648E-26	ENSMUSG00000008845	Cd163
2.00335	1.16186	2.60354224216525E-27	2.57744613111324E-26	ENSMUSG00000018486	Wnt9b
1.65009	-1.20074	2.66747588098999E-27	2.63920094736505E-26	ENSMUSG00000071068	Treml2
1.12829	3.84297	3.18968193984602E-27	3.13034759064727E-26	ENSMUSG00000036814	Slc6a20a
1.52876	-1.99203	3.73308284222152E-27	3.6530882098882E-26	ENSMUSG00000033080	Vsx1
1.03419	-0.43727	3.77213139513267E-27	3.68705225204337E-26	ENSMUSG00000042821	Snai1
-0.80799	-0.93285	3.98286567791484E-27	3.88855874347226E-26	ENSMUSG00000071537	Klrg2
-0.72570	4.14195	5.27046586478497E-27	5.11919234482373E-26	ENSMUSG00000078137	Ankrd63
1.02797	-0.23177	6.5876448597066E-27	6.38032559160181E-26	ENSMUSG00000029032	Arhgef16
1.23901	-0.03735	6.66851979998629E-27	6.45497517733146E-26	ENSMUSG00000070473	Cldn3
0.71769	5.55372	6.89592919922688E-27	6.66750399752227E-26	ENSMUSG00000028581	Laptm5
-0.77752	-0.31939	7.40977505455825E-27	7.13994660390445E-26	ENSMUSG00000072919	Noxred1
0.80594	1.88545	7.89721592495418E-27	7.58811674961095E-26	ENSMUSG00000048126	Col6a3
-0.70119	0.15567	9.17800766773026E-27	8.79390830566281E-26	ENSMUSG00000030769	Slc5a11
2.40235	-1.91909	9.18381511753252E-27	8.79451246993475E-26	ENSMUSG00000070504	Fcrl6
0.86368	-0.02803	9.28209271666116E-27	8.8836163983459E-26	ENSMUSG00000068758	Il3ra
0.72373	-0.78259	1.08278802588213E-26	1.03339342604975E-25	ENSMUSG00000039187	Fanci
1.89930	-1.86069	1.37151582721846E-26	1.30018475852607E-25	ENSMUSG00000026358	Rgs1
1.10153	-1.77843	1.4310992488729E-26	1.35591266256848E-25	ENSMUSG00000040829	Zmynd15
-0.73573	-0.03002	1.66453585935722E-26	1.56833805761289E-25	ENSMUSG00000030669	Calca
1.82754	-0.43095	1.8108505682989E-26	1.70242000300286E-25	ENSMUSG00000050377	Il31ra
1.04933	1.23257	2.59856777876407E-26	2.42818863727415E-25	ENSMUSG00000025491	Ifitm1

0.88259	0.41445	2.74403371234251E-26	2.55568227550848E-25	ENSMUSG00000071715	Ncf4
1.49626	-2.31782	2.82064238925272E-26	2.62559303608905E-25	ENSMUSG00000053063	Clec12a
2.01418	-3.00263	2.93309399140011E-26	2.72429746997841E-25	ENSMUSG00000068587	Mgam
1.11173	-1.33126	3.14885021954864E-26	2.91513174548732E-25	ENSMUSG00000005320	Fgfr4
0.88141	-1.37662	3.2440927267964E-26	3.0000352336863E-25	ENSMUSG00000022357	Klhl38
0.73041	1.14833	3.67363667017237E-26	3.38988265903793E-25	ENSMUSG00000029915	Clec5a
0.86821	2.01600	4.61976420140757E-26	4.24219212181145E-25	ENSMUSG00000037405	Icam1
1.63384	1.35989	5.10834447011545E-26	4.67819708131112E-25	ENSMUSG00000004885	Crabp2
1.18154	-1.67218	5.87471124304584E-26	5.35980636932668E-25	ENSMUSG00000021213	Akr1c13
0.78862	-0.39558	5.97012509570194E-26	5.44393371582311E-25	ENSMUSG00000012123	Crybg2
2.92798	0.80372	6.33917490715176E-26	5.76807195086739E-25	ENSMUSG00000040380	Cbln3
1.47703	2.89288	6.38444226158166E-26	5.8061512387446E-25	ENSMUSG00000024650	Slc22a6
0.81012	-0.80393	6.51456309010726E-26	5.9213160928166E-25	ENSMUSG00000019989	Enpp3
0.72816	0.53631	7.60305161712165E-26	6.88856751315534E-25	ENSMUSG00000037731	Themis2
1.10281	2.14230	7.86623786803403E-26	7.11942721908162E-25	ENSMUSG00000051439	Cd14
3.46744	-2.70957	8.12666628463226E-26	7.34730212045412E-25	ENSMUSG00000050359	Sprr1a
1.08817	1.27747	8.82784083947169E-26	7.95468114296695E-25	ENSMUSG00000040310	Alx4
2.38396	-1.21153	9.61189275957012E-26	8.63494628236791E-25	ENSMUSG00000031074	Fgf3
1.70491	0.99083	1.01463658925544E-25	9.0910582163879E-25	ENSMUSG00000030109	Slc6a12
0.77485	-1.34115	1.05499950439377E-25	9.44772355331647E-25	ENSMUSG00000032015	Pou2f3
-1.95309	2.46347	1.06938081216375E-25	9.57146535144243E-25	ENSMUSG00000037868	Egr2
1.13260	-1.09091	1.10245773402404E-25	9.8519473885326E-25	ENSMUSG00000022014	Epsti1
0.72765	0.35946	1.17117207005559E-25	1.04440268378501E-24	ENSMUSG00000034112	Atp2c2
0.73438	-0.47818	1.38411076747128E-25	1.23041725367881E-24	ENSMUSG00000021469	Msx2
1.07636	0.63198	1.75613880786432E-25	1.5513929312532E-24	ENSMUSG00000029219	Slc10a4
1.58567	-1.31500	1.76604680850261E-25	1.55852484066714E-24	ENSMUSG00000021675	F2rl2
0.70236	1.08422	1.84037233197282E-25	1.62243098990941E-24	ENSMUSG00000029217	Tec
1.08124	-1.21960	1.94787947946435E-25	1.71099155104139E-24	ENSMUSG00000039232	Stx11
0.75826	1.16540	2.23410900027184E-25	1.95634245858856E-24	ENSMUSG00000044338	Aplnr
0.86273	-0.91643	2.38066168997906E-25	2.0793152076794E-24	ENSMUSG00000020437	Myo1g
3.48430	-2.59939	2.40387485021626E-25	2.09851109740359E-24	ENSMUSG00000030827	Fgf21
1.08406	-1.67759	3.05978601205132E-25	2.66425652346119E-24	ENSMUSG00000068105	Tnfrsf13c
1.32005	-1.11066	3.13548452496454E-25	2.72597805067029E-24	ENSMUSG00000063234	Gpr84
1.26300	-1.78471	3.48972001799157E-25	3.02311900385726E-24	ENSMUSG00000061540	Orm2
1.54724	0.69702	3.68453067926167E-25	3.18376435296527E-24	ENSMUSG00000034394	Lif
0.86176	-0.82946	3.9073302076852E-25	3.37113893185151E-24	ENSMUSG00000009214	Mymk
1.47650	-1.65383	4.09972747497439E-25	3.53175306008443E-24	ENSMUSG00000049641	Vgll2
1.53089	-1.29343	4.44532162732411E-25	3.82558884523719E-24	ENSMUSG00000039865	Slc44a3
0.88718	4.33836	5.57280390953065E-25	4.7837692175395E-24	ENSMUSG00000035783	Acta2
1.01947	-1.47475	6.8451014505617E-25	5.83465044867748E-24	ENSMUSG00000035683	Melk
3.08158	-1.72573	7.59528112191599E-25	6.47084431790917E-24	ENSMUSG00000046623	Gjb4
0.90734	-1.50281	7.79583863155849E-25	6.63505544453485E-24	ENSMUSG00000075304	Sp5
0.95031	-1.19379	8.13382113389643E-25	6.91924654094305E-24	ENSMUSG00000046056	Sbsn
1.10582	2.28367	8.97282324099006E-25	7.60630345398898E-24	ENSMUSG00000046618	Olfml2a
0.70247	0.34331	1.02548015456243E-24	8.68437530693248E-24	ENSMUSG00000031952	Chst5
-0.78959	2.03493	1.04739206342027E-24	8.86551887064456E-24	ENSMUSG00000028011	Tdo2
0.93637	2.73510	1.04854239061808E-24	8.87083572301789E-24	ENSMUSG00000028626	Col9a2
0.73645	2.70744	1.06534246552085E-24	9.00848073880948E-24	ENSMUSG00000015947	Fcgr1
0.78300	-0.00492	1.07575743809252E-24	9.09202356135109E-24	ENSMUSG00000054626	Xlr
0.81470	0.94392	1.28933752836639E-24	1.08809070699892E-23	ENSMUSG00000051212	Gpr183
1.56895	0.20721	1.30923366831645E-24	1.1043327486276E-23	ENSMUSG00000046714	Foxc2

0.74580	-0.93075	1.54680736687071E-24	1.29827883144267E-23	ENSMUSG00000078157	4931440F15Rik
1.33904	-1.28005	1.54979838445311E-24	1.29950518040916E-23	ENSMUSG00000048015	Neurod4
0.85402	-1.24990	1.64407832590058E-24	1.37584249263049E-23	ENSMUSG00000002104	Rapsn
3.92203	-2.12298	1.72363762932725E-24	1.43887744702758E-23	ENSMUSG00000033831	Fgb
1.12039	-2.51323	1.77676137479373E-24	1.48249618050078E-23	ENSMUSG00000046460	Sh2d7
0.74788	-0.43634	1.79049032901766E-24	1.49248526542453E-23	ENSMUSG00000041673	Lrrc18
0.90851	-0.29814	1.81601113707078E-24	1.51301604691312E-23	ENSMUSG00000038583	Pln
1.87792	2.11115	1.84300500446438E-24	1.53475338312945E-23	ENSMUSG00000036594	H2-Aa
0.73874	0.48969	1.91599031340604E-24	1.59474980128084E-23	ENSMUSG00000027611	Procr
0.82912	-0.40397	2.02127570456821E-24	1.6799134867517E-23	ENSMUSG00000019942	Cdk1
0.74015	6.23029	2.34985460115214E-24	1.94728438850597E-23	ENSMUSG00000060802	B2m
2.30151	0.13839	2.50884130531941E-24	2.07498520422426E-23	ENSMUSG00000055333	Fat2
0.75688	0.49547	2.56741788963605E-24	2.1223987887658E-23	ENSMUSG00000031377	Bmx
2.41988	-2.40905	2.57273772139512E-24	2.12576208225001E-23	ENSMUSG00000079298	Klrb1b
0.74790	0.61745	2.67965301012188E-24	2.20980317164808E-23	ENSMUSG00000028150	Rorc
1.88289	-0.29929	2.71922231259271E-24	2.24025939118938E-23	ENSMUSG00000071230	Npw
2.39106	-1.47161	2.75810948194051E-24	2.27119553461975E-23	ENSMUSG00000028362	Tnfsf8
1.45365	-1.50438	2.78535081149808E-24	2.29140627533799E-23	ENSMUSG00000014198	Zfp385c
2.40288	-3.38239	3.15652896865945E-24	2.58798813318469E-23	ENSMUSG00000051207	Mrgprd
2.23220	-0.96499	3.48554125388061E-24	2.84674878946749E-23	ENSMUSG00000037379	Spon2
3.90625	-2.76860	3.93523526864711E-24	3.20324756798166E-23	ENSMUSG00000074336	Apoc4
0.85521	-1.09499	3.96616615264813E-24	3.22687886021008E-23	ENSMUSG00000028678	Kif2c
1.66608	-2.68861	4.57615142384248E-24	3.70895326279752E-23	ENSMUSG00000026866	Kynu
0.97750	2.67450	4.77760934771502E-24	3.86669974268617E-23	ENSMUSG00000022766	Serpind1
0.70414	1.43620	5.29403185745773E-24	4.27651037539191E-23	ENSMUSG00000021835	Bmp4
0.86817	0.77545	5.31061963130921E-24	4.28787102170536E-23	ENSMUSG00000085925	Rtl1
0.75010	1.88477	6.21116685319913E-24	4.99598970180619E-23	ENSMUSG00000030798	Cd37
-0.73374	1.54090	6.79699204360096E-24	5.45944684807059E-23	ENSMUSG00000040740	Slc25a34
0.73028	0.51741	7.16862339207624E-24	5.74979103798825E-23	ENSMUSG00000030745	Il21r
0.80185	1.21681	7.76138656137996E-24	6.2164278597229E-23	ENSMUSG00000022876	Samsn1
0.88341	0.88067	8.75924719857073E-24	6.99586701501267E-23	ENSMUSG00000024679	Ms4a6d
1.31741	0.02099	1.11880836743247E-23	8.90225599341584E-23	ENSMUSG00000001349	Cnn1
2.60514	-0.90534	1.11977407546578E-23	8.90576872378867E-23	ENSMUSG00000023964	Calcr
0.76535	0.87660	1.24659865346648E-23	9.89589622667692E-23	ENSMUSG00000040666	Sh3bgr
1.78868	-3.05608	1.43682530955553E-23	1.13634955115127E-22	ENSMUSG00000057135	Scimp
0.82108	-1.14423	1.52951263646333E-23	1.20796655826309E-22	ENSMUSG00000062175	Tgif2
1.07102	0.33086	1.60868841601302E-23	1.269317176555E-22	ENSMUSG00000024371	C2
0.75393	-0.55704	1.70963189256525E-23	1.34646391241995E-22	ENSMUSG00000040084	Bub1b
1.35228	-2.66733	1.93395080811932E-23	1.51820500593027E-22	ENSMUSG00000054672	5830411N06Rik
0.78785	1.49811	2.36660905300941E-23	1.84845768241489E-22	ENSMUSG00000022901	Cd86
0.96241	-0.41096	2.68378503055528E-23	2.08755220233851E-22	ENSMUSG00000032085	Tagln
1.91197	-0.88371	2.8733737160258E-23	2.23093568043173E-22	ENSMUSG00000035352	Ccl12
0.82151	0.78877	4.65409240181076E-23	3.56303387660933E-22	ENSMUSG00000096727	Psmb9
1.56627	-1.04976	5.71297835657415E-23	4.35993155083026E-22	ENSMUSG00000073409	H2-Q6
1.10584	-0.89042	5.96468696682891E-23	4.54589960486718E-22	ENSMUSG00000050824	Sstr5
1.70663	-2.53441	6.46925671267384E-23	4.92161813859844E-22	ENSMUSG00000043664	Tmem221
0.99118	-0.17176	6.5188950160469E-23	4.95716152786951E-22	ENSMUSG00000070337	Gpr179
-1.02262	-2.24757	6.81943014962543E-23	5.18337715354974E-22	ENSMUSG00000030666	Calcb
1.58639	-2.48168	9.40854955527357E-23	7.08791307516574E-22	ENSMUSG00000024669	Cd5
0.98169	-1.64766	1.00995692409538E-22	7.60511889473949E-22	ENSMUSG00000022667	Cd200r1
1.01136	1.69331	1.45739215982563E-22	1.08588500048762E-21	ENSMUSG00000035929	H2-Q4

0.79914	-1.20415	1.56655857960724E-22	1.16466945953469E-21	ENSMUSG00000020897	Aurkb
0.99871	-1.38576	2.04301351017186E-22	1.51095835919894E-21	ENSMUSG00000020317	Theg
0.73423	-0.04849	2.24852504626145E-22	1.6588706317826E-21	ENSMUSG00000042638	Gucy2c
1.87712	-1.07808	2.27249046552902E-22	1.67556718873294E-21	ENSMUSG00000041534	Rbp3
1.09131	-1.99367	2.2988260743223E-22	1.69351506290491E-21	ENSMUSG00000013974	Mcemp1
0.75579	0.41598	2.72070518141826E-22	1.99997142457522E-21	ENSMUSG00000025877	Hk3
1.32749	-1.97109	2.97642141571964E-22	2.18228083773177E-21	ENSMUSG00000040522	Tlr8
0.73276	-0.82614	3.05314943833328E-22	2.23757129673881E-21	ENSMUSG00000035365	Parpbp
0.72547	4.15023	3.16862102484138E-22	2.32019542974161E-21	ENSMUSG00000041548	Hspb8
2.30336	-2.78431	3.21566426622779E-22	2.35362794289865E-21	ENSMUSG00000028327	Stra6l
0.93083	-0.92305	3.22377527040848E-22	2.35854841919463E-21	ENSMUSG00000020963	Tshr
1.97366	-3.29960	3.54816162067295E-22	2.58696006918421E-21	ENSMUSG00000051051	Olfr523
1.56173	0.76825	3.60452546236009E-22	2.62692743691863E-21	ENSMUSG00000048368	Omd
0.91144	1.89780	3.85388386675753E-22	2.80624856958753E-21	ENSMUSG00000021763	BC067074
0.73634	-0.47761	5.5862870026689E-22	4.04345307206388E-21	ENSMUSG00000062168	Ppef1
3.08593	-3.55852	5.80936029729473E-22	4.20312660691835E-21	ENSMUSG00000026272	Agxt
0.92166	-1.81751	6.30098789287869E-22	4.54913652036647E-21	ENSMUSG00000079343	C1s2
0.72861	-0.80281	6.49461589164207E-22	4.68295987976297E-21	ENSMUSG00000003206	Ebi3
3.19540	-3.46131	6.55782549989513E-22	4.72653116640723E-21	ENSMUSG00000068008	Bpifb3
0.91442	-1.67531	6.94999966306251E-22	5.00070284947505E-21	ENSMUSG00000021898	Asb14
1.02397	-0.30987	7.68718728753163E-22	5.51943946071798E-21	ENSMUSG00000022367	Has2
1.11376	-1.14381	7.97091087439251E-22	5.72073654136798E-21	ENSMUSG00000090124	Ugt1a7c
4.62836	-2.41618	8.46094641494858E-22	6.06730931604671E-21	ENSMUSG00000015854	Cd5l
0.74127	-0.25041	8.57489661362478E-22	6.14642800304885E-21	ENSMUSG00000033952	Aspm
1.94449	-3.08093	1.09506129102419E-21	7.78689879109207E-21	ENSMUSG00000063935	Zar1
-0.74950	1.97005	1.25729342513281E-21	8.91068031128752E-21	ENSMUSG00000022715	Tmem114
1.79160	-3.12051	1.6459125890993E-21	1.15971642735873E-20	ENSMUSG00000020703	5530401A14Rik
1.51695	-2.60665	2.00011454687199E-21	1.40346740695008E-20	ENSMUSG00000036480	Prss56
1.78721	-1.34245	2.66627459102242E-21	1.86321154883953E-20	ENSMUSG00000059430	Actg2
2.53697	-3.11416	3.04697946063542E-21	2.12313728782914E-20	ENSMUSG00000020010	Vnn3
1.00648	-1.86041	3.20605931939096E-21	2.22758837291672E-20	ENSMUSG00000049409	Prokr1
1.56624	-1.98864	3.5382066571927E-21	2.44935023196372E-20	ENSMUSG00000054003	Tdrd9
1.12144	1.56281	3.59994751555333E-21	2.48904796069271E-20	ENSMUSG00000067212	H2-T23
0.91293	-0.96945	3.73003890733971E-21	2.57689715160175E-20	ENSMUSG00000029061	Mmp23
0.75937	-1.50332	4.85783474687462E-21	3.33973681424144E-20	ENSMUSG00000045102	Poln
0.84772	-1.10876	5.21109784824495E-21	3.57392532280926E-20	ENSMUSG00000038523	1700003F12Rik
0.76642	-0.72648	6.37953739365527E-21	4.36470323171227E-20	ENSMUSG00000000320	Alox12
0.76455	-0.83569	6.51791070919266E-21	4.457579191939E-20	ENSMUSG00000046814	Gchfr
1.15860	-1.97422	6.56171922177542E-21	4.48573384867287E-20	ENSMUSG00000028459	Cd72
1.12028	-0.82135	6.6961510636139E-21	4.57579301161195E-20	ENSMUSG00000074151	Nlrc5
0.96430	0.28193	7.61101915595507E-21	5.19052562911942E-20	ENSMUSG00000070407	Hs3st3b1
1.14309	-1.72589	7.7129066495144E-21	5.25579053998999E-20	ENSMUSG00000074577	Ripor3
1.07917	-2.30393	8.35407827068309E-21	5.68130831314509E-20	ENSMUSG00000043017	Ptgir
0.95773	-0.60991	9.07773893636847E-21	6.16850516204111E-20	ENSMUSG00000079547	H2-DMb1
2.05472	-3.12838	9.44649326632494E-21	6.40882698116326E-20	ENSMUSG00000026070	Il18r1
1.22344	1.78128	1.00748933105415E-20	6.81610065947747E-20	ENSMUSG00000048721	Fndc9
1.80038	-3.16488	1.07985791840145E-20	7.28539567823823E-20	ENSMUSG00000074155	Klk5
0.84891	-0.09274	1.20731682280348E-20	8.11951630474489E-20	ENSMUSG00000074071	Fam169b
-0.75139	0.08564	1.22654875036198E-20	8.24232997276479E-20	ENSMUSG00000045053	Kcng3
1.02806	0.82608	1.26017654466339E-20	8.46161230859354E-20	ENSMUSG00000028036	Ptgfr
1.73781	-2.30804	1.4238879393596E-20	9.51573891181781E-20	ENSMUSG00000035385	Ccl2

1.24542	-1.81029	1.79987641025241E-20	1.1962558864385E-19	ENSMUSG00000042254	Cilp
0.87804	-1.77406	1.85448300342617E-20	1.23206715925709E-19	ENSMUSG00000024791	Cdca5
1.14550	0.27533	1.85648752012431E-20	1.23256128544181E-19	ENSMUSG00000031698	Mylk3
0.94932	-0.81913	1.85667784874358E-20	1.23256128544181E-19	ENSMUSG00000000154	Slc22a18
0.87664	1.95530	1.91103479519908E-20	1.26666637147257E-19	ENSMUSG00000022025	Cnmd
1.09582	-2.04587	2.14887975751464E-20	1.41933006689964E-19	ENSMUSG00000042377	Fam83g
0.78465	-1.30753	2.17664552070825E-20	1.437110536564E-19	ENSMUSG00000019178	Styxl1
0.70241	-0.64315	2.3575427772708E-20	1.55473356755731E-19	ENSMUSG00000074676	Foxs1
0.75239	-1.48735	2.98879390121058E-20	1.95885921272243E-19	ENSMUSG00000024979	Tectb
0.86681	-1.95305	3.16484203625552E-20	2.07184341086354E-19	ENSMUSG00000046591	Ticrr
-1.57027	-3.22856	3.26829867684101E-20	2.13709999700443E-19	ENSMUSG00000040127	Sdr9c7
0.76659	3.71920	3.27261564135919E-20	2.13909944268603E-19	ENSMUSG00000046402	Rbp1
2.11298	-2.34449	3.31245086525022E-20	2.16347232982971E-19	ENSMUSG00000046213	Cym
0.91134	-1.70511	3.43337396413191E-20	2.23946441313266E-19	ENSMUSG00000035745	Grin3b
0.88983	0.72951	3.57152990441556E-20	2.32731683990071E-19	ENSMUSG00000028369	Svep1
0.80602	-0.93618	3.63107730300414E-20	2.36521247022371E-19	ENSMUSG00000025058	5430427O19Rik
0.72253	-1.36423	3.93772552159132E-20	2.56201000232836E-19	ENSMUSG00000097271	Gm9903
1.11399	0.28393	3.96794476449308E-20	2.58068321819328E-19	ENSMUSG00000054901	Arhgef33
2.49853	-2.39182	3.99707505486277E-20	2.59764005478228E-19	ENSMUSG00000005952	Trpv1
1.96077	0.85130	4.61126656827245E-20	2.98082939352406E-19	ENSMUSG00000018822	Sfrp5
1.06881	-0.19977	4.83946859939208E-20	3.12240381946345E-19	ENSMUSG00000050195	Scd4
1.46250	-0.50499	4.85791055683999E-20	3.13311254895967E-19	ENSMUSG00000040966	Slc22a2
1.95941	-2.15799	5.92085252025139E-20	3.79416984587064E-19	ENSMUSG00000047798	Cd300lf
1.28005	-2.55081	6.65481466688799E-20	4.24688172656248E-19	ENSMUSG00000039264	Gimap3
1.09501	-2.06094	6.72341025996888E-20	4.28743594205523E-19	ENSMUSG00000039699	Batf2
2.36170	-3.48532	8.10064728823599E-20	5.14251854008045E-19	ENSMUSG00000051076	Vtcn1
-0.88453	1.61011	8.15377244696572E-20	5.1723781302858E-19	ENSMUSG00000004328	Hif3a
0.77149	-1.38895	8.8144289534549E-20	5.56859498182566E-19	ENSMUSG00000042333	Tnfrsf14
0.78614	0.60928	9.7178930277077E-20	6.12569820982183E-19	ENSMUSG00000038943	Prc1
0.82947	-1.22962	9.8191087173977E-20	6.18490985877465E-19	ENSMUSG00000020808	Pimreg
1.68783	-3.08430	1.0083282609838E-19	6.34895496574976E-19	ENSMUSG00000032093	Cd3e
1.18156	0.33844	1.06645280219739E-19	6.70747878701568E-19	ENSMUSG00000062329	Cytl1
0.99821	1.08692	1.12939511165603E-19	7.09547491006385E-19	ENSMUSG00000049241	Hcar1
1.20784	9.04659	1.39570003933801E-19	8.72338199715753E-19	ENSMUSG00000015090	Ptgds
0.74151	0.14971	1.52455229232062E-19	9.49380291126934E-19	ENSMUSG00000027868	Tbx15
0.80025	0.04035	1.67551055452412E-19	1.0399551808643E-18	ENSMUSG00000049608	Gpr55
0.77100	-0.48424	1.82383809772252E-19	1.12995483603611E-18	ENSMUSG00000023931	Efhb
0.77917	0.10689	1.9307514953069E-19	1.19401552247083E-18	ENSMUSG00000041845	Rhod
0.85733	1.81990	2.29847902836651E-19	1.4137060729142E-18	ENSMUSG00000039109	F13a1
1.53066	-3.00347	2.3958024943946E-19	1.4709032444805E-18	ENSMUSG00000032726	Bmp8a
1.91444	-1.90333	2.48814051388279E-19	1.52538906711804E-18	ENSMUSG00000052854	Nrk
0.93471	1.15120	2.73222777402579E-19	1.67141107040512E-18	ENSMUSG00000026208	Des
1.07771	1.47286	2.95307767713736E-19	1.80521351490499E-18	ENSMUSG00000044177	Wfikkn2
0.84946	1.92006	2.9965732854284E-19	1.83114341628984E-18	ENSMUSG00000032243	Itga11
0.73492	-1.24867	3.01295171296566E-19	1.84048988492847E-18	ENSMUSG00000016756	Cmah
2.23223	-2.39544	3.02508371569284E-19	1.84723659820956E-18	ENSMUSG00000034115	Scn11a
0.74162	-0.39174	3.421720211744E-19	2.08344741781746E-18	ENSMUSG00000027399	Il1a
1.18107	-1.39782	3.58976755718628E-19	2.18107908660517E-18	ENSMUSG00000078129	Actl10
-0.72704	0.37856	3.91211973060316E-19	2.36846364873437E-18	ENSMUSG00000000766	Oprm1
0.77254	1.62711	4.18185231833312E-19	2.52996108204569E-18	ENSMUSG00000058396	Gpr182
0.71880	-1.37245	4.47726849630974E-19	2.70483062643349E-18	ENSMUSG00000037544	Dlgap5

0.87838	2.76339	4.56739776834417E-19	2.75731888019299E-18	ENSMUSG00000044468	Tent5c
1.23762	-2.78168	4.57173608604995E-19	2.75895746464712E-18	ENSMUSG00000067878	Map7d3
1.59562	-2.25563	4.66955780155162E-19	2.81599034195097E-18	ENSMUSG00000038216	Pnmt
1.36576	-0.95417	5.12154189117936E-19	3.08308836454128E-18	ENSMUSG00000032092	Mpzl2
4.07427	-3.20579	5.35189382072037E-19	3.21947493719538E-18	ENSMUSG00000027919	Lce1g
1.29695	-2.32304	5.50826554202802E-19	3.31119656857651E-18	ENSMUSG00000059108	Ifitm6
1.21032	-2.51861	6.39209343500007E-19	3.83164725736701E-18	ENSMUSG00000034107	Ano7
0.95616	-1.04851	6.97078291545018E-19	4.17117506754729E-18	ENSMUSG00000035187	Nkx6-1
0.93668	-2.04753	7.0756652827562E-19	4.23095395365936E-18	ENSMUSG00000000244	Tspan32
1.85424	-3.22679	7.54889246431195E-19	4.50916262952642E-18	ENSMUSG00000036912	Piwil4
0.72788	0.22196	8.99134007983703E-19	5.35195813862198E-18	ENSMUSG00000022780	Meltf
2.34948	-3.45774	9.76668708379184E-19	5.80533520571924E-18	ENSMUSG00000108398	Gm30191
1.20211	0.81049	1.01490086421713E-18	6.01784847515551E-18	ENSMUSG00000035273	Hpse
1.01630	-0.61423	1.02172176654465E-18	6.05617912423603E-18	ENSMUSG00000025927	Tfap2b
1.37986	-1.73688	1.0940761611131E-18	6.47376030128506E-18	ENSMUSG00000028487	Bnc2
-1.29356	-1.04507	1.22443644777834E-18	7.21996750255411E-18	ENSMUSG00000033383	Rtp1
1.69802	-2.49221	1.2440910419915E-18	7.33331666250923E-18	ENSMUSG00000045518	Onecut3
1.98631	-2.83344	1.26740341867069E-18	7.46814057453264E-18	ENSMUSG00000112023	Lilr4b
0.90074	-1.43746	1.40689095197887E-18	8.2728499453849E-18	ENSMUSG00000006221	Hspb7
1.15335	-2.41688	1.49602788827078E-18	8.78179743121768E-18	ENSMUSG00000032725	Folr2
2.19808	-1.95968	1.5445883510961E-18	9.0605894020789E-18	ENSMUSG00000032021	Crtam
-0.78781	-1.60368	1.63696034950092E-18	9.58460842690006E-18	ENSMUSG00000074817	Papolb
1.32770	1.36486	1.65120437307365E-18	9.66264550112819E-18	ENSMUSG00000006014	Prg4
0.80664	-1.39425	1.68331425223787E-18	9.84376678726916E-18	ENSMUSG00000051378	Kif18b
0.91317	3.79100	1.75125965249827E-18	1.02270192425716E-17	ENSMUSG00000030218	Mgp
0.74490	-1.46589	1.83078752470184E-18	1.0669440298331E-17	ENSMUSG00000041498	Kif14
0.77135	-0.54616	1.85165967484457E-18	1.07836799987177E-17	ENSMUSG00000026870	Cutal
0.86601	-0.89715	1.96383965505763E-18	1.14213310715916E-17	ENSMUSG00000024670	Cd6
0.74633	-0.87796	2.02159644551012E-18	1.17411556978892E-17	ENSMUSG00000027843	Ptpn22
0.86824	-1.05992	2.08296218712245E-18	1.20851644927719E-17	ENSMUSG00000063011	Msln
0.80737	0.86543	2.16606998231944E-18	1.25587702592637E-17	ENSMUSG00000025498	Irf7
0.91985	2.02430	2.2234915385987E-18	1.28785115096197E-17	ENSMUSG00000026837	Col5a1
0.74926	-1.47844	2.43023311972555E-18	1.40424490605094E-17	ENSMUSG00000024056	Ndc80
1.23440	-1.98712	3.76032488078487E-18	2.14922597330442E-17	ENSMUSG00000030116	Mfap5
4.02053	-2.90346	3.92039085455228E-18	2.23638683133425E-17	ENSMUSG00000075402	Krt76
1.67778	-3.22826	3.95644580801358E-18	2.2561967568491E-17	ENSMUSG00000067702	Tuba3a
1.32159	-2.47135	4.06263761855646E-18	2.31364692806025E-17	ENSMUSG00000043740	B430306N03Rik
1.40325	-2.68293	4.08357898843395E-18	2.32479356084169E-17	ENSMUSG00000109713	Pvrig
0.86541	-1.27565	4.28261068639338E-18	2.43402443427403E-17	ENSMUSG00000031849	Comp
0.88662	-0.20629	4.31910230234971E-18	2.45394347532833E-17	ENSMUSG00000042655	Shisal2b
0.86832	-1.14830	4.41806299273352E-18	2.50932979339877E-17	ENSMUSG00000068245	Phf11d
0.75635	0.88162	4.58207732759951E-18	2.59987740952774E-17	ENSMUSG00000030708	Dnajb13
1.57388	-3.16086	5.07057930586241E-18	2.86921040905904E-17	ENSMUSG00000046589	Lrrc8e
2.50058	-2.70090	5.26628397094901E-18	2.97616873248443E-17	ENSMUSG00000024173	Tpsab1
1.69676	-2.79198	5.28578973974767E-18	2.98520598732824E-17	ENSMUSG00000071551	Akr1c19
1.16942	-2.23240	5.78309846623771E-18	3.26064642364574E-17	ENSMUSG00000032358	Fam83b
1.09693	0.98147	6.36023087491133E-18	3.57536737600906E-17	ENSMUSG00000047420	Fam180a
0.70955	-1.79213	6.49583464630506E-18	3.64918118291767E-17	ENSMUSG00000050957	Insl6
1.14191	-2.25920	6.89045951163845E-18	3.86448089084563E-17	ENSMUSG00000053469	Tg
1.19849	-2.73273	6.99481627566632E-18	3.9178351101556E-17	ENSMUSG00000055865	Tafa3
0.81029	-1.71318	7.34784116974177E-18	4.11420981514743E-17	ENSMUSG00000020325	Fstl3

1.14155	-0.92846	8.06269790532787E-18	4.49816459821707E-17	ENSMUSG00000039518	Cdsn
0.70450	1.02533	1.00722718190038E-17	5.59358462442746E-17	ENSMUSG00000051498	Tlr6
0.86053	0.03162	1.01416793032094E-17	5.62844978774654E-17	ENSMUSG00000030107	Usp18
1.52580	-3.11124	1.09396955128068E-17	6.05550822325062E-17	ENSMUSG00000006204	5430419D17Rik
0.76295	-1.07294	1.16323445697979E-17	6.41800160934481E-17	ENSMUSG00000042784	Muc1
0.83916	-2.08588	1.16890744433191E-17	6.44720768321767E-17	ENSMUSG00000072244	Trim6
0.93822	-1.11073	1.23140942231468E-17	6.77434691265602E-17	ENSMUSG00000026589	Sec16b
0.74768	-1.16404	1.32438222672721E-17	7.27874644698863E-17	ENSMUSG00000070390	Nlrp1b
0.95277	-1.66483	1.3511140034351E-17	7.41846305441353E-17	ENSMUSG00000047182	Irs3
0.95829	-2.29171	1.37076037552669E-17	7.52390218398945E-17	ENSMUSG00000044313	Mab21l3
0.84827	0.02965	1.52150414279248E-17	8.32173611647092E-17	ENSMUSG00000025150	Cbr2
1.52937	-3.26923	1.53509575467818E-17	8.39337196024232E-17	ENSMUSG00000095901	Olfr538
0.93652	4.96649	1.58876904414621E-17	8.67566972740463E-17	ENSMUSG00000041324	Inhba
0.73057	-1.19967	1.68248633001472E-17	9.163859498009E-17	ENSMUSG00000037628	Cdkn3
1.48424	-2.62190	1.76064780480351E-17	9.57116317056066E-17	ENSMUSG00000048217	Nags
2.46795	-3.34085	2.10606051717983E-17	1.14050864092607E-16	ENSMUSG00000079620	Muc4
0.99913	-2.45110	2.40509089106672E-17	1.29789339445494E-16	ENSMUSG00000031448	Adprhl1
0.79801	-0.76145	2.41717606530236E-17	1.30358688880497E-16	ENSMUSG00000020264	Slc36a2
0.99117	-2.31930	2.73687651735158E-17	1.47086549436155E-16	ENSMUSG00000078795	Ceacam15
0.82196	-1.06139	2.89689920594969E-17	1.55538949780889E-16	ENSMUSG00000051456	Hspb3
1.05450	-1.94003	2.97561929931959E-17	1.59563827830938E-16	ENSMUSG00000046031	Calhm6
1.22888	-2.74480	3.09959310949645E-17	1.66106901401027E-16	ENSMUSG00000017204	Gsdma
1.67860	0.76198	3.11099213388706E-17	1.66665198267024E-16	ENSMUSG00000000303	Cdh1
1.01533	-2.66549	3.22244989379951E-17	1.72527509599326E-16	ENSMUSG00000054362	Lexm
1.01897	-1.63669	3.29078029320584E-17	1.7600821477127E-16	ENSMUSG00000043230	Fam124b
2.44652	-2.04943	3.74432772613381E-17	1.99525217727607E-16	ENSMUSG00000036136	Fam110c
1.67043	-2.46852	3.93218865259336E-17	2.09142206732173E-16	ENSMUSG00000037446	Tulp1
0.77832	0.17347	4.06151070430763E-17	2.15750293448337E-16	ENSMUSG00000042631	Xkr7
1.68571	-0.38655	4.35000700708427E-17	2.30786755266545E-16	ENSMUSG00000031289	Il13ra2
0.95337	-1.23716	4.49861089877111E-17	2.38298727621839E-16	ENSMUSG00000037548	H2-DMb2
0.82945	-1.90291	5.42583130973594E-17	2.85899572859163E-16	ENSMUSG00000025461	Cd163l1
1.46533	-0.59095	5.64454083402109E-17	2.96687684679301E-16	ENSMUSG00000069378	Prdm6
0.89289	-1.54961	5.7388182020729E-17	3.01456535611671E-16	ENSMUSG00000052760	A630001G21Rik
0.83443	0.93401	6.37015348991822E-17	3.33485878233377E-16	ENSMUSG00000071714	Csf2rb2
5.56451	-0.27778	6.62426683491416E-17	3.46517866011794E-16	ENSMUSG00000020428	Gabra6
0.90177	4.51872	7.09243738379732E-17	3.70385263682597E-16	ENSMUSG00000015852	Fcrls
-0.91450	-2.03181	7.25573490554419E-17	3.78796633605976E-16	ENSMUSG00000092622	Khdc3
1.09375	-1.87391	7.53085880071488E-17	3.92436286216394E-16	ENSMUSG00000031097	Tnni2
1.14768	-2.46014	7.64928600274597E-17	3.98485343804503E-16	ENSMUSG00000052396	Mogat2
1.85074	-2.36537	7.89316683978872E-17	4.10812249614984E-16	ENSMUSG00000051596	Otop1
0.74524	-1.20394	8.0207085654397E-17	4.17194724769411E-16	ENSMUSG00000000730	Dnmt3l
0.80756	-1.75760	8.16913198840388E-17	4.24654878271129E-16	ENSMUSG00000032028	Nxpe2
0.90160	-1.37203	9.09957775534726E-17	4.71866993003173E-16	ENSMUSG00000049037	Clec4a1
1.51281	-1.22933	9.42486791412793E-17	4.88288063815814E-16	ENSMUSG00000060550	H2-Q7
1.67940	-1.98951	9.80316646184855E-17	5.07268327303939E-16	ENSMUSG00000027514	Zbp1
0.71020	-0.90064	1.05812751741558E-16	5.46865538967323E-16	ENSMUSG00000078942	Naip6
1.71928	-2.03766	1.21002188635008E-16	6.22527310881743E-16	ENSMUSG00000048699	Krt90
1.14561	-1.24626	1.39853339821744E-16	7.16690358036737E-16	ENSMUSG00000043583	4930470P17Rik
-0.80742	-1.93353	1.59362636374495E-16	8.14456217427776E-16	ENSMUSG00000079495	Nat8f6
1.92798	-3.43331	1.60387946881476E-16	8.19449756878954E-16	ENSMUSG00000016524	Il19
0.80775	-0.56858	1.60624482078603E-16	8.20411515800155E-16	ENSMUSG00000053318	Slamf8

1.76220	-3.33889	1.64547507007566E-16	8.40196287659914E-16	ENSMUSG00000097084	Foxl1
1.02631	-2.38223	1.71335963154078E-16	8.73808268406328E-16	ENSMUSG00000036602	Alx1
1.01099	-2.20009	1.73600989708211E-16	8.85094121597566E-16	ENSMUSG00000007107	Atp1a4
1.47680	0.58623	1.86440597565809E-16	9.49416328371691E-16	ENSMUSG00000033227	Wnt6
1.51588	-3.24720	1.89355019784188E-16	9.6367977114853E-16	ENSMUSG00000045027	Prss22
1.31653	-2.63891	2.00758801422316E-16	1.01957863036242E-15	ENSMUSG00000076433	Cep295nl
2.69989	-2.13924	2.02566326533616E-16	1.02828811614157E-15	ENSMUSG00000044681	Cnpy1
1.39587	-2.75193	2.10466022090413E-16	1.06632770154248E-15	ENSMUSG00000054932	Afp
1.54649	-3.12784	2.65263863197746E-16	1.3356369725713E-15	ENSMUSG00000031410	Nxf7
1.03841	-2.48811	2.65795384117463E-16	1.33747985349155E-15	ENSMUSG00000068860	Gm128
1.31315	0.43304	2.68754087546475E-16	1.35141890368502E-15	ENSMUSG00000010122	Slc47a1
2.30925	-3.72114	2.68804125003042E-16	1.35141890368502E-15	ENSMUSG00000046790	Olfr1341
0.71477	-1.22175	2.95184645431819E-16	1.48185483350938E-15	ENSMUSG00000070687	Htr1d
0.84155	-1.65636	2.95867242507118E-16	1.4848427520564E-15	ENSMUSG00000047531	Rtp2
2.31436	-3.63938	3.31848846671333E-16	1.65856081413728E-15	ENSMUSG00000070661	Rnf186
3.31570	-3.18619	3.52027874142902E-16	1.7563141045344E-15	ENSMUSG00000007946	Phox2a
2.16680	-3.62241	3.62980317815766E-16	1.81042561334534E-15	ENSMUSG00000030954	Gp2
0.87419	-2.13249	3.64873522874868E-16	1.81933413753985E-15	ENSMUSG00000065968	Ifitm7
0.76154	-1.26988	3.78277235220039E-16	1.88561434035153E-15	ENSMUSG00000054342	Kcnn4
0.78794	1.49215	3.82570677782104E-16	1.90589755840539E-15	ENSMUSG00000078853	Igtp
2.39095	-3.42785	5.64945304695317E-16	2.79072138300786E-15	ENSMUSG00000053773	Rdh8
2.35442	-3.66088	6.73064792345249E-16	3.30845621885448E-15	ENSMUSG00000030968	Pdilt
1.54290	-2.98465	7.2870827218762E-16	3.57060747848955E-15	ENSMUSG00000048067	Olfr1349
1.58450	-1.25699	8.20464490434234E-16	4.0063382476277E-15	ENSMUSG00000005640	Insrr
1.04107	-2.48564	9.17666321548308E-16	4.4630161667514E-15	ENSMUSG00000020838	Slc6a4
-0.90912	-0.28897	9.46304428157533E-16	4.59834657481126E-15	ENSMUSG00000079494	Nat8f5
0.96192	2.52947	1.10344253098147E-15	5.33597543874557E-15	ENSMUSG00000057722	Lepr
0.95259	-1.63650	1.25393476976189E-15	6.04479110917E-15	ENSMUSG00000050010	Shisa3
0.74829	0.00853	1.33249292613159E-15	6.41257502241455E-15	ENSMUSG00000026638	Irf6
1.22365	-2.81530	1.48491945018542E-15	7.127949030729E-15	ENSMUSG00000031506	Ptpn7
2.15167	-3.64584	1.61656598491224E-15	7.7401981261807E-15	ENSMUSG00000053852	Adgrg4
1.04704	-1.18995	1.62794473892154E-15	7.79248386159457E-15	ENSMUSG00000031722	Hp
1.10943	-2.91809	1.6349538317372E-15	7.82382977283143E-15	ENSMUSG00000102418	Sh2d1b1
2.68053	-2.52229	1.69261017893273E-15	8.09061950470155E-15	ENSMUSG00000029417	Cxcl9
-1.28153	4.57761	2.06653506153143E-15	9.81719732251004E-15	ENSMUSG00000021250	Fos
1.40733	-2.85772	2.11000347402995E-15	1.00180936324262E-14	ENSMUSG00000043727	F830045P16Rik
0.91346	-2.64681	2.49797620886364E-15	1.18073510952074E-14	ENSMUSG00000046908	Ltb4r1
0.76672	-0.69499	2.63494140009319E-15	1.24270917559087E-14	ENSMUSG00000029373	Pf4
0.75824	3.67977	2.73177984277032E-15	1.28730862604666E-14	ENSMUSG00000008999	Bmp7
-0.70992	-1.24976	2.89266731997193E-15	1.36010607339284E-14	ENSMUSG00000021509	Slc25a48
0.80838	-0.23674	2.93302706373996E-15	1.37796961880078E-14	ENSMUSG00000070034	Sp110
0.88074	-1.89316	2.96587059992313E-15	1.39298340479663E-14	ENSMUSG00000056671	Prelid2
0.75984	1.48066	2.99944358192115E-15	1.40797312986119E-14	ENSMUSG00000034459	Ifit1
0.82386	-1.95730	3.10597095874694E-15	1.45717300875982E-14	ENSMUSG00000109764	Klkb1
0.74230	2.53800	3.26374947198668E-15	1.52908372945697E-14	ENSMUSG00000035493	Tgfbi
0.94544	1.10491	3.28887137569092E-15	1.5404286443407E-14	ENSMUSG00000040564	Apoc1
1.32422	-2.25936	3.32023668789912E-15	1.55426235475421E-14	ENSMUSG00000090145	Ugt1a6b
0.92687	0.74557	3.38968475490414E-15	1.58633511339701E-14	ENSMUSG00000032323	Cyp11a1
0.90604	-0.18676	3.50237390349248E-15	1.63591773089168E-14	ENSMUSG00000078920	Ifi47
0.83828	-0.78731	3.53462931721894E-15	1.65007647268248E-14	ENSMUSG00000052776	Oas1a
1.36136	-2.50758	3.57963330601779E-15	1.67062666490743E-14	ENSMUSG00000013643	Lypd8

1.42500	-2.30886	3.83355431906159E-15	1.78569840340604E-14	ENSMUSG00000029664	Tfpi2
1.75952	-3.22095	4.09490804611276E-15	1.90482743393657E-14	ENSMUSG00000071177	Serpina1d
0.84012	5.82715	4.51486846993667E-15	2.09616249159016E-14	ENSMUSG00000073411	H2-D1
-1.68551	3.25730	5.34033015536351E-15	2.47062986599442E-14	ENSMUSG00000045903	Npas4
1.24853	-1.76216	5.94450283097785E-15	2.7419281589099E-14	ENSMUSG00000069792	Wfdc17
1.03472	-1.53418	6.64964473710411E-15	3.05804452609433E-14	ENSMUSG00000028716	Pdzk1ip1
1.41172	-1.64332	6.93417344865232E-15	3.18372266339745E-14	ENSMUSG00000105096	Gbp10
0.70944	-0.58366	6.9868491802414E-15	3.2070411746539E-14	ENSMUSG00000036961	Wnt8b
0.85614	-1.89120	7.41968676885622E-15	3.39928907306714E-14	ENSMUSG00000026068	Il18rap
1.94748	-3.61508	8.52703486857492E-15	3.88669890923935E-14	ENSMUSG00000044703	Phf11a
1.09410	-1.43021	9.86321412329284E-15	4.47652368491848E-14	ENSMUSG00000055210	Foxd2
0.80780	-1.22698	9.90383642370048E-15	4.49375996703589E-14	ENSMUSG00000014603	Alx3
0.73516	0.02441	9.98425988649541E-15	4.52541640746489E-14	ENSMUSG00000028037	Ifi44
0.80710	-1.03457	1.11190786921949E-14	5.02770585102495E-14	ENSMUSG00000003309	Ap1m2
1.24609	-2.15057	1.13469076653948E-14	5.12390397181624E-14	ENSMUSG00000020017	Hal
0.82034	-1.53634	1.25777923799884E-14	5.67068834796291E-14	ENSMUSG00000026874	Hc
1.74579	-3.10985	1.2997724033356E-14	5.8537999967829E-14	ENSMUSG00000005202	Shbg
1.26243	-2.41662	1.35851951042722E-14	6.11028049858027E-14	ENSMUSG00000030873	Scnn1b
1.25930	-2.98898	1.51134326002161E-14	6.78506852569955E-14	ENSMUSG00000097918	Ascl5
0.73465	-1.13852	1.58441683390344E-14	7.10561593831883E-14	ENSMUSG00000032715	Trib3
0.89255	-0.31675	1.68405778951164E-14	7.54450783972145E-14	ENSMUSG00000040950	Mgl2
0.75853	-1.88325	1.72116352273274E-14	7.70058623233707E-14	ENSMUSG00000030433	Sbk2
0.87880	-2.26812	1.74060260816843E-14	7.78345804358127E-14	ENSMUSG00000075031	Hist1h2bb
2.29564	-3.70678	1.9252767389099E-14	8.58666349188798E-14	ENSMUSG00000028751	Pla2g2e
-0.99531	-2.29131	2.61574840272679E-14	1.15419049001358E-13	ENSMUSG00000032948	Lipi
0.85052	1.90213	2.65244354748426E-14	1.1700781870855E-13	ENSMUSG00000046718	Bst2
0.74456	0.43850	2.94577893575044E-14	1.2961122134299E-13	ENSMUSG00000030214	Plbd1
1.04348	-2.61942	2.98796098368746E-14	1.31263204527754E-13	ENSMUSG00000046974	BC053393
1.03002	-2.61800	3.10488060651415E-14	1.36188256502614E-13	ENSMUSG00000029923	Rab19
0.72391	-1.09489	3.10589383112694E-14	1.36197533307136E-13	ENSMUSG00000021565	Slc6a19
1.47293	-3.10251	3.43663246407138E-14	1.5014800588435E-13	ENSMUSG00000002033	Cd3g
0.84252	-1.47897	3.43728275773627E-14	1.5014800588435E-13	ENSMUSG00000038167	Plekhg6
0.71487	2.20244	3.53360809811886E-14	1.54197108581667E-13	ENSMUSG00000032122	Slc37a2
0.90227	-2.28824	3.66486864590022E-14	1.59801818676984E-13	ENSMUSG00000048904	Neurog1
1.07522	-2.30858	4.49000687352875E-14	1.94433435553164E-13	ENSMUSG00000058755	Osm
0.85117	-1.81688	4.7967136194849E-14	2.07292218183184E-13	ENSMUSG00000058163	Gm5431
1.35293	-3.22394	5.21719580504531E-14	2.2466342797493E-13	ENSMUSG00000039639	Kcne1
1.31395	-3.15952	5.32073974891263E-14	2.28774302339984E-13	ENSMUSG00000050232	Cxcr3
1.13283	-2.03753	5.69206384785056E-14	2.44183789513347E-13	ENSMUSG00000024334	H2-Oa
1.65405	-3.32095	5.74701305734157E-14	2.46416602266832E-13	ENSMUSG00000023045	Soat2
0.84892	1.27070	5.8013581347947E-14	2.48558567449918E-13	ENSMUSG00000020216	Jsrp1
1.22542	-1.63410	7.36137688483513E-14	3.13499800751013E-13	ENSMUSG00000034127	Tspan8
1.42138	-2.88164	7.83961190428202E-14	3.33031575468725E-13	ENSMUSG00000030361	Klrb1a
1.66010	-3.24777	8.12575510532467E-14	3.44928355145566E-13	ENSMUSG00000029865	Sval1
0.72166	-1.32919	8.19249056297504E-14	3.47674318470697E-13	ENSMUSG00000027379	Bub1
0.95835	-2.59512	8.84770760969142E-14	3.7426508185617E-13	ENSMUSG00000024936	Kcnk7
2.54933	-1.99109	8.85946937844149E-14	3.74669319892865E-13	ENSMUSG00000025330	Padi4
1.02651	-2.98056	9.67482716421256E-14	4.0813499842474E-13	ENSMUSG00000047129	1700113H08Rik
1.03463	-1.99206	9.758726161824E-14	4.11572095424693E-13	ENSMUSG00000058818	Pirb
0.75620	-1.85026	9.80031312171978E-14	4.13120891592495E-13	ENSMUSG00000040432	Ltb4r2
1.19079	-2.88738	1.1420522224354E-13	4.79486506106285E-13	ENSMUSG00000034435	Tmem30b

1.00394	-2.04039	1.19274590255101E-13	4.9968846837328E-13	ENSMUSG00000019987	Arg1
0.74584	-0.64775	1.25814889370087E-13	5.25533154811666E-13	ENSMUSG00000010311	Optc
0.70609	-1.27578	1.50908907619743E-13	6.27267071848345E-13	ENSMUSG00000003227	Edar
0.85999	-1.87634	1.52161774906888E-13	6.32320017641444E-13	ENSMUSG00000036526	Card11
1.43626	-2.55073	1.58213854109142E-13	6.56666736771588E-13	ENSMUSG00000043144	Aqp6
0.81290	0.00560	1.69201127627173E-13	7.01412580802931E-13	ENSMUSG00000044165	Bcl2l15
1.04953	-2.68191	1.863012617331E-13	7.68735932553291E-13	ENSMUSG00000024034	Tmprss3
1.12140	-3.17061	2.19453116986195E-13	9.01370781276953E-13	ENSMUSG00000074604	Mgst2
0.98698	0.01387	2.26445283583406E-13	9.2919141968959E-13	ENSMUSG00000051985	Igfn1
1.53723	-2.42140	2.46344771114898E-13	1.00889705200094E-12	ENSMUSG00000069855	Slc47a2
0.97542	-0.83966	2.52702395346727E-13	1.03393744993984E-12	ENSMUSG00000026630	Batf3
1.09830	-2.90793	2.96546870138916E-13	1.2083804821108E-12	ENSMUSG00000041644	Slc5a12
0.72633	-0.16524	3.15462734749861E-13	1.28361220070195E-12	ENSMUSG00000056380	Gpr50
0.91557	-2.58666	3.72601569411203E-13	1.51140292768804E-12	ENSMUSG00000043003	Rasef
0.93519	-1.65588	3.74545264094449E-13	1.51856203972232E-12	ENSMUSG00000073490	Ifi207
0.99825	-0.77969	3.82191768993194E-13	1.54919440984404E-12	ENSMUSG00000046223	Plaur
0.73283	2.24818	4.42900153908965E-13	1.78844492859651E-12	ENSMUSG00000031351	Zfp185
0.72365	-2.10977	4.67282082543901E-13	1.88510757973303E-12	ENSMUSG00000054582	Pabpc1l
1.05834	-0.44836	4.7407170289312E-13	1.91159033675488E-12	ENSMUSG00000079017	Ifi27l2a
0.70721	0.25730	5.27986761751664E-13	2.12244181463258E-12	ENSMUSG00000024043	Arhgap28
1.21231	-2.76451	5.79992782072731E-13	2.32489791926653E-12	ENSMUSG00000047631	Apof
1.24760	-2.76324	5.86316466490425E-13	2.3491377671555E-12	ENSMUSG00000056290	Ms4a4b
0.87898	-2.59686	5.9129485816206E-13	2.36573646972611E-12	ENSMUSG00000082079	Dnmt3c
1.49899	-2.68814	5.96566165425979E-13	2.38626466170392E-12	ENSMUSG00000022303	Dcstamp
-1.66325	-3.10791	6.42434509347091E-13	2.56189611380009E-12	ENSMUSG00000058523	Mup5
1.51858	-3.07266	6.63627040077648E-13	2.64268545636172E-12	ENSMUSG00000044303	Cdkn2a
0.92655	-2.58747	6.6392216738566E-13	2.64324110137042E-12	ENSMUSG00000052180	Serpinb6c
1.35665	-3.25535	7.17256576228662E-13	2.84956845579339E-12	ENSMUSG00000030217	Art4
0.76284	-1.55981	7.56595209791744E-13	3.00304659437901E-12	ENSMUSG00000062310	Glrp1
1.40911	-3.38809	7.73201736368948E-13	3.06681090297355E-12	ENSMUSG00000040963	Asgr2
1.28167	-2.48264	9.09917132758395E-13	3.59480750030224E-12	ENSMUSG00000102037	Bcl2a1a
0.80571	-0.91079	1.06328380444962E-12	4.17933023368584E-12	ENSMUSG00000051980	Casr
0.83336	0.90690	1.11357846965045E-12	4.36288538801242E-12	ENSMUSG00000026628	Atf3
0.71825	-2.14381	1.15360448267957E-12	4.51241836328818E-12	ENSMUSG00000020234	4930404N11Rik
1.10887	-0.92614	1.18594386666795E-12	4.63464789670007E-12	ENSMUSG00000026205	Slc23a3
0.81452	-2.44989	1.29263531274562E-12	5.03537920039502E-12	ENSMUSG00000000903	Vpreb3
0.97614	-1.79802	1.36505649303468E-12	5.30955637003011E-12	ENSMUSG00000025279	Dnase1l3
0.91836	-2.47833	1.36520733601904E-12	5.30955637003011E-12	ENSMUSG00000023393	Slc17a9
0.87181	-1.78865	1.44043055514592E-12	5.59315069047289E-12	ENSMUSG00000031877	Ces2g
0.94581	-0.18766	1.51421547806319E-12	5.8702630171925E-12	ENSMUSG00000022483	Col2a1
1.31734	-3.28227	1.67942921088416E-12	6.49889372084284E-12	ENSMUSG00000050366	Olfr524
0.98312	-3.02727	1.68644810447802E-12	6.5245685262748E-12	ENSMUSG00000016758	Bik
1.71858	-3.48026	1.74495619116888E-12	6.73405628704612E-12	ENSMUSG00000061126	Cyp4f39
0.83330	-2.66649	2.06726401657422E-12	7.95079943707558E-12	ENSMUSG00000047841	Fndc11
0.74047	-1.66526	2.09237017966091E-12	8.04189697105873E-12	ENSMUSG00000074651	Mcidas
0.95097	-2.56338	2.14694783918534E-12	8.24420205517191E-12	ENSMUSG00000026228	Htr2b
1.52461	-3.03631	2.66156919429759E-12	1.01560506452667E-11	ENSMUSG00000038580	Sct
1.19893	-3.09869	2.69235504670676E-12	1.02643015110984E-11	ENSMUSG00000012396	Nanog
0.93016	-2.07259	2.72527978754659E-12	1.03874922662871E-11	ENSMUSG00000086564	Cd101
1.66026	-2.54237	2.76462580638049E-12	1.05327345141941E-11	ENSMUSG00000079015	Serpina1c
1.71496	-3.63020	3.02671223434328E-12	1.14874413398176E-11	ENSMUSG00000014453	Blk

1.36108	-2.37089	3.07618429459798E-12	1.16699907987116E-11	ENSMUSG00000020609	Apob
1.27659	-1.69581	3.11520807974311E-12	1.18048527456337E-11	ENSMUSG00000026602	Nphs2
-0.80017	4.15346	3.16884373405733E-12	1.20000707432381E-11	ENSMUSG00000003545	Fosb
1.46562	-2.82770	3.23267777960396E-12	1.22363480659341E-11	ENSMUSG00000043263	Ifi209
1.02490	-3.01674	3.24996849957006E-12	1.22990565539533E-11	ENSMUSG00000005763	Cd247
1.23676	-2.47619	3.3434074761721E-12	1.26245401656394E-11	ENSMUSG00000029491	Pde6b
0.74094	-1.84899	3.43963356260346E-12	1.29792303335201E-11	ENSMUSG00000023349	Clec4n
0.85935	-2.14816	3.72108522867064E-12	1.40225811589745E-11	ENSMUSG00000039628	Hs3st6
1.52906	-2.96180	3.76564688441419E-12	1.41810705547391E-11	ENSMUSG00000067006	Serpinb5
0.95283	-2.63237	3.98776431418023E-12	1.50009167779658E-11	ENSMUSG00000073774	Kncn
0.93905	4.31308	4.03514985836567E-12	1.51690917888728E-11	ENSMUSG00000029843	Slc13a4
1.22183	-3.13268	4.67356387901103E-12	1.74916288117734E-11	ENSMUSG00000027249	F2
1.58238	-3.22440	4.94585809048416E-12	1.8457872856139E-11	ENSMUSG00000075610	Tmem92
0.89419	-1.49120	5.1976306845211E-12	1.93719504319097E-11	ENSMUSG00000073402	Gm8909
0.71867	0.00026	5.33916956883049E-12	1.98820281971268E-11	ENSMUSG00000027831	Veph1
0.85894	-1.26619	5.47367136899693E-12	2.03694916136955E-11	ENSMUSG00000026938	Fcna
0.72571	-1.85304	7.43387560901776E-12	2.73821940255841E-11	ENSMUSG00000079243	Xirp1
1.51079	-3.52206	7.53236860926465E-12	2.7720944093195E-11	ENSMUSG00000068009	Bpifb6
0.98297	-2.52226	7.86999917438475E-12	2.89015156299013E-11	ENSMUSG00000035458	Tnni3
0.83530	-1.65718	7.94769124237865E-12	2.91735909303216E-11	ENSMUSG00000028965	Tnfrsf9
1.76008	-1.89130	7.97362855352986E-12	2.92624761001005E-11	ENSMUSG00000004630	Pcp2
0.73337	-2.56632	8.14046501684938E-12	2.98682979927078E-11	ENSMUSG00000091685	Gm17359
0.83358	-1.66954	8.74034350304847E-12	3.19726432880679E-11	ENSMUSG00000027398	Il1b
1.03439	-1.69297	8.97003960678843E-12	3.27987586827641E-11	ENSMUSG00000026725	Tnn
1.30025	-2.27452	1.13922358502736E-11	4.12910822753251E-11	ENSMUSG00000035042	Ccl5
1.73477	-3.70357	1.18789615971632E-11	4.30093349557989E-11	ENSMUSG00000072849	Serpina1e
0.94324	-2.34306	1.29810956520426E-11	4.68698943542826E-11	ENSMUSG00000064109	Hcst
0.94680	-2.49966	1.39695169306879E-11	5.02784223768065E-11	ENSMUSG00000028841	Cnksr1
0.90669	-2.81866	1.45425741860371E-11	5.22523794992384E-11	ENSMUSG00000030219	Erp27
1.60945	-3.72432	1.51591802626193E-11	5.43758771751218E-11	ENSMUSG00000037129	Tmprss13
1.35256	-3.59434	1.6099727331673E-11	5.76400775364514E-11	ENSMUSG00000045569	Mc2r
0.86097	2.60781	1.78511829174578E-11	6.37226088257561E-11	ENSMUSG00000004105	Angptl2
1.52824	-1.66512	1.80027070188946E-11	6.42269516679368E-11	ENSMUSG00000034438	Gbp8
0.80619	0.55065	1.81909643162488E-11	6.48673597406453E-11	ENSMUSG00000043719	Col6a6
0.95823	-0.92676	1.84527197007235E-11	6.57869469624115E-11	ENSMUSG00000074207	Adh1
0.74491	-1.42863	1.95760283127936E-11	6.96601526974734E-11	ENSMUSG00000028463	Car9
1.56587	-3.38182	1.9688604954068E-11	7.00460776878969E-11	ENSMUSG00000053977	Cd8a
1.09302	-3.07295	2.00550928980628E-11	7.13349912379168E-11	ENSMUSG00000068246	Apol9b
1.05216	-0.87038	2.24480912190166E-11	7.96300216388922E-11	ENSMUSG00000041567	Serpina12
1.01945	-3.06797	2.33567433959775E-11	8.27841345317892E-11	ENSMUSG00000028175	Depdc1a
1.08455	0.55574	2.6362172116547E-11	9.29322639377256E-11	ENSMUSG00000029371	Cxcl5
1.39810	-3.22951	2.85841735431158E-11	1.00514994856231E-10	ENSMUSG00000052477	C130026I21Rik
1.15764	-2.92494	3.01051664407589E-11	1.05557599070302E-10	ENSMUSG00000033501	Crygs
0.99945	0.63128	3.0305114667745E-11	1.06214831437106E-10	ENSMUSG00000040569	Slc26a7
0.72684	-1.96449	3.09861117449192E-11	1.08556829515918E-10	ENSMUSG00000027938	Creb3l4
1.13243	-0.80669	3.26439590649016E-11	1.14176565080203E-10	ENSMUSG00000049103	Ccr2
1.35186	-3.52532	4.033183970598E-11	1.40343566760587E-10	ENSMUSG00000099398	Ms4a14
0.86204	-2.18368	4.15606065352195E-11	1.44471369719728E-10	ENSMUSG00000047109	Cldn14
1.31485	-2.88455	4.21331472843155E-11	1.46371759931687E-10	ENSMUSG00000098470	C1rb
0.90456	-2.65002	4.52997433650158E-11	1.57051436792834E-10	ENSMUSG00000096094	A630095N17Rik
0.98623	-2.10317	4.80201102754654E-11	1.65974696512636E-10	ENSMUSG00000028834	Trim63

1.49812	-1.03906	5.68131443827688E-11	1.95333271964071E-10	ENSMUSG00000002930	Ppp1r17
1.20949	-2.81469	5.86826176492691E-11	2.01434695620486E-10	ENSMUSG00000035373	Ccl7
1.03326	0.34507	6.13273473297414E-11	2.10173285543201E-10	ENSMUSG00000060586	H2-Eb1
0.97068	-3.07441	6.28104721355354E-11	2.15082503656213E-10	ENSMUSG00000108763	Gm36028
1.24199	-3.32812	7.12083349872396E-11	2.43153204977533E-10	ENSMUSG00000055301	Adh7
0.74515	-2.34862	7.22195547436886E-11	2.46408073104194E-10	ENSMUSG00000037106	Fer1l6
1.13583	-3.13836	7.70167957675605E-11	2.61986649278998E-10	ENSMUSG00000070691	Runx3
0.74241	-2.28248	8.15106366174249E-11	2.76774474286791E-10	ENSMUSG00000110622	Iqcn
1.20507	-3.22644	8.34617716488236E-11	2.82947231444865E-10	ENSMUSG00000024401	Tnf
0.98844	-2.84918	8.34940326782438E-11	2.83000125127295E-10	ENSMUSG00000011008	Mcoln2
1.42740	-3.10688	8.75239921430776E-11	2.96068812928435E-10	ENSMUSG00000026304	Rab17
0.78532	-2.78392	8.84637386207506E-11	2.9895006797082E-10	ENSMUSG00000052271	Bhlha15
0.89483	0.59210	9.02234993035127E-11	3.04654503313074E-10	ENSMUSG00000054072	Iigp1
0.77079	-2.47101	9.59152692488041E-11	3.2329535595212E-10	ENSMUSG00000026357	Rgs18
1.00869	-2.21505	1.01399751298785E-10	3.41239892049081E-10	ENSMUSG00000024675	Ms4a4c
0.77600	-1.58261	1.02751531218688E-10	3.45515243931726E-10	ENSMUSG00000037577	Ephx3
1.09071	-3.35117	1.03254481108263E-10	3.47000420389155E-10	ENSMUSG00000031553	Adam3
0.73848	-1.81613	1.05723142240114E-10	3.5487546737306E-10	ENSMUSG00000071540	3425401B19Rik
1.02655	-2.83328	1.13361760941159E-10	3.79167079123529E-10	ENSMUSG00000040592	Cd79b
0.72264	-0.58019	1.31241882448189E-10	4.3724987233376E-10	ENSMUSG00000000318	Clec10a
0.89742	-2.08183	1.34893748038024E-10	4.48624704712206E-10	ENSMUSG00000026308	Klhl30
1.54984	-3.55296	1.67038358574748E-10	5.52071524410081E-10	ENSMUSG00000067604	Nms
1.59358	-3.57781	1.7307178044357E-10	5.71234390164245E-10	ENSMUSG00000090894	Olfr110
0.82205	-1.50845	1.83818520827784E-10	6.04590325619052E-10	ENSMUSG00000017607	Tns4
1.55317	-3.68242	1.90560315358618E-10	6.26279481004487E-10	ENSMUSG00000022877	Hrg
0.89225	-1.64819	1.91823809389415E-10	6.30188140380464E-10	ENSMUSG00000026832	Cytip
1.05071	-3.10252	1.95755039041045E-10	6.42606106904207E-10	ENSMUSG00000018168	Ikzf3
1.40198	-3.61614	2.45230161412982E-10	8.00224737242362E-10	ENSMUSG00000032921	Odf4
1.03502	-3.21044	2.61482607819827E-10	8.50156275912577E-10	ENSMUSG00000049565	Aknad1
1.45888	-3.79258	3.0578394193332E-10	9.89458591535854E-10	ENSMUSG00000035592	Krt33a
0.81839	-2.20024	3.09900597310535E-10	1.00201586355374E-09	ENSMUSG00000000673	Haao
1.09936	-1.22906	3.10403870211082E-10	1.00345213076039E-09	ENSMUSG00000104713	Gbp6
0.90947	-2.82603	3.13989850849667E-10	1.01446549757211E-09	ENSMUSG00000094796	BC147527
0.95774	-1.63248	3.57847619119265E-10	1.15134760484812E-09	ENSMUSG00000024134	Six2
0.79353	-2.73844	3.68870130717587E-10	1.18300279037764E-09	ENSMUSG00000034689	4921530L21Rik
0.80330	-2.04320	3.777104411568E-10	1.20975583981367E-09	ENSMUSG00000041827	Oasl1
0.99199	-1.55205	3.81374653504941E-10	1.22057132888884E-09	ENSMUSG00000029082	Bst1
1.73835	-2.99522	4.16959048164195E-10	1.32894940154096E-09	ENSMUSG00000052974	Cyp2f2
0.70955	-1.48308	4.20695209792265E-10	1.34010317343915E-09	ENSMUSG00000073010	Gm5127
1.25212	-3.61393	4.52188649896502E-10	1.43745897912458E-09	ENSMUSG00000020583	Matn3
0.78138	-2.19275	4.52914197215519E-10	1.43895761778516E-09	ENSMUSG00000070873	Lilra5
0.77388	-2.32568	4.66581857640213E-10	1.48127314475648E-09	ENSMUSG00000034266	Batf
1.01521	-2.42016	6.27304884784818E-10	1.97382022276801E-09	ENSMUSG00000054763	Defb42
0.85596	-2.73148	6.2808835615148E-10	1.97591944339958E-09	ENSMUSG00000021359	Tfap2a
0.74338	-2.58009	6.63878029484081E-10	2.08503604453236E-09	ENSMUSG00000040471	Ggt6
0.74197	-2.40006	6.83084342475654E-10	2.14100310147166E-09	ENSMUSG00000048292	Olfr1417
1.05261	-1.96199	7.41625954405265E-10	2.3189290840119E-09	ENSMUSG00000091705	H2-Q2
1.29103	-1.89931	7.42178208680345E-10	2.32022882021746E-09	ENSMUSG00000002100	Mybpc3
-1.03713	-3.04235	7.69197440596571E-10	2.40133823191535E-09	ENSMUSG00000029378	Areg
1.40381	-3.63599	7.98800658348862E-10	2.49101253065586E-09	ENSMUSG00000066677	Ifi208
0.89448	-2.69989	8.72244038609342E-10	2.70889976743976E-09	ENSMUSG00000024124	Prss30

0.88991	-2.37053	8.75237110085047E-10	2.71720175916023E-09	ENSMUSG00000021880	Rnase6
0.83262	-3.05326	9.29029789320545E-10	2.87684251931779E-09	ENSMUSG00000021396	Nxnl2
0.89828	-1.60318	1.00022999505073E-09	3.09168616374124E-09	ENSMUSG00000051367	Six1
1.14680	-3.21277	1.10532439473797E-09	3.40229223007948E-09	ENSMUSG00000071005	Ccl19
1.19311	-3.02627	1.27521985141398E-09	3.90613682578807E-09	ENSMUSG00000026413	Pkp1
0.86926	-2.88489	1.27801067505621E-09	3.91397969133854E-09	ENSMUSG00000021322	Aoah
0.80124	-2.97469	1.32597332866757E-09	4.05648026425439E-09	ENSMUSG00000022408	Fam83f
1.52516	-3.65133	1.33295624562122E-09	4.07637456356676E-09	ENSMUSG00000022824	Muc13
1.19702	-2.18002	1.37798596923885E-09	4.20953527161115E-09	ENSMUSG00000026989	Dapl1
0.89857	-1.36168	1.45409592829353E-09	4.4340659899211E-09	ENSMUSG00000067144	Slc22a7
0.80452	-1.58431	1.52092498948941E-09	4.62788352524557E-09	ENSMUSG00000027318	Adam33
0.87704	-2.03004	1.62782603304915E-09	4.93989079125385E-09	ENSMUSG00000003379	Cd79a
1.23135	-3.29589	1.7362801555296E-09	5.25586729902652E-09	ENSMUSG00000024678	Ms4a4d
1.71165	-3.45107	1.74964444249866E-09	5.29349239344028E-09	ENSMUSG00000035861	Tmprss11b
0.71985	-2.49473	1.77651574405839E-09	5.37383359331621E-09	ENSMUSG00000006469	Slc34a3
1.15617	-3.39172	1.78701497705078E-09	5.40270696389726E-09	ENSMUSG00000097050	Gm9918
1.47205	-2.88892	1.87556984266711E-09	5.65533909961464E-09	ENSMUSG00000066366	Serpina1a
0.76414	-2.49487	2.08051420709449E-09	6.25664283061095E-09	ENSMUSG00000038765	Lmx1b
-0.96104	-3.09028	2.10246975654286E-09	6.31931284928346E-09	ENSMUSG00000087512	Cccd201
1.09888	-0.76124	2.37297925802761E-09	7.09596402664549E-09	ENSMUSG00000063975	Slco1a5
0.89503	-3.06071	2.4260808885866E-09	7.24837533157038E-09	ENSMUSG00000037542	Aldh8a1
1.13511	-3.03859	2.564530092603E-09	7.64682781690786E-09	ENSMUSG00000005883	Spo11
0.75840	-2.41441	2.79943196408184E-09	8.32576159065516E-09	ENSMUSG00000032053	Pou2af1
0.80706	-2.89415	2.81711488690426E-09	8.3739541030148E-09	ENSMUSG00000079580	Tmem217
0.82293	-2.77963	3.026474629161E-09	8.97116576516962E-09	ENSMUSG00000025127	Gcgr
1.53323	-3.72807	3.17563337766422E-09	9.4018229034088E-09	ENSMUSG00000027470	Mylk2
1.35718	-3.64959	3.53368667056511E-09	1.04219217290903E-08	ENSMUSG00000053675	Tgm5
1.27804	-3.09385	3.6910582936121E-09	1.08747308865561E-08	ENSMUSG00000042477	Tfap2e
1.16987	-3.53033	3.84610694722329E-09	1.13040942594168E-08	ENSMUSG00000003555	Cyp17a1
0.70290	-2.44627	3.92477607216204E-09	1.15233487580174E-08	ENSMUSG00000044217	Aqp5
1.18901	-2.79092	4.08458061482247E-09	1.1975984722921E-08	ENSMUSG00000068606	Gm4841
0.79671	-1.70066	4.22150815834086E-09	1.23582596232801E-08	ENSMUSG00000070369	Itgad
0.94537	-2.84216	4.25349524235566E-09	1.24454662723283E-08	ENSMUSG00000037953	A4gnt
1.22381	-3.28487	4.4624079729236E-09	1.30320417129149E-08	ENSMUSG00000087166	L1td1
1.09806	-3.31131	5.14592264674787E-09	1.49510747260053E-08	ENSMUSG00000030365	Clec2i
1.40286	-3.68387	5.65546663365773E-09	1.63671323973727E-08	ENSMUSG00000053654	Krt42
1.10415	-3.32983	5.88698813707E-09	1.70110825774018E-08	ENSMUSG00000025726	Slc28a1
0.76096	-2.30976	7.00690172576304E-09	2.0158043440688E-08	ENSMUSG00000025163	Cd7
1.23238	-3.11185	7.18600581610227E-09	2.06558150260483E-08	ENSMUSG00000030402	Ppm1n
0.73761	3.81337	7.23668384858623E-09	2.07979673861923E-08	ENSMUSG00000021390	Ogn
1.99833	-3.58484	7.46481724781368E-09	2.14427317223299E-08	ENSMUSG00000041044	Lrit1
0.72976	0.64820	7.77632082978621E-09	2.22885335340658E-08	ENSMUSG00000020660	Pomc
0.73543	-1.91107	8.07972348354574E-09	2.31308295481084E-08	ENSMUSG00000032818	Loxhd1
1.41443	-2.24346	8.80341675991597E-09	2.51179784879833E-08	ENSMUSG00000022225	Cma1
1.12628	-3.60971	8.83032145360062E-09	2.5190512318013E-08	ENSMUSG00000066755	Tnfsf18
0.70023	-0.85362	9.95600648129838E-09	2.82641440682314E-08	ENSMUSG00000013483	Card14
1.07004	-2.84494	1.00522614142766E-08	2.8527867842588E-08	ENSMUSG00000010601	Apol7a
1.01359	-3.18697	1.11172317927125E-08	3.14451438052946E-08	ENSMUSG00000030325	Klrb1c
-0.70493	-2.70566	1.14073664047133E-08	3.22228700504272E-08	ENSMUSG00000102422	Iqschfp
0.83621	-2.94271	1.14216438906247E-08	3.22524744704011E-08	ENSMUSG00000051998	Lax1
1.13975	-3.44170	1.15943111947824E-08	3.27237346032504E-08	ENSMUSG00000062524	Ncr1

0.71747	-2.43818	1.20468409401128E-08	3.39333002637435E-08	ENSMUSG00000031844	Hsd17b2
1.03076	-3.37634	1.25428965737361E-08	3.52779349328857E-08	ENSMUSG00000049109	Themis
0.94746	-3.24557	1.29146222159495E-08	3.6257412362345E-08	ENSMUSG00000094018	S100a2
-0.94165	-1.08194	1.29779444068521E-08	3.64231487830174E-08	ENSMUSG00000032899	Styk1
1.00050	-2.59299	1.30383069724552E-08	3.65865145107481E-08	ENSMUSG00000021922	Itih4
0.74244	4.69830	1.33287839582088E-08	3.73892638510653E-08	ENSMUSG00000016918	Sulf1
1.35912	-3.74868	1.38442219941337E-08	3.87839121431963E-08	ENSMUSG00000063230	Olfr535
1.63384	-2.24519	1.48039205893039E-08	4.14178199886519E-08	ENSMUSG00000069515	Lyz1
0.78277	-2.95260	1.49011114094493E-08	4.16760093223122E-08	ENSMUSG00000074277	Phldb3
0.80268	-2.82166	1.76263449471651E-08	4.91281949077015E-08	ENSMUSG00000070291	Ddx43
0.95558	-2.74612	1.80729780722832E-08	5.03235127834695E-08	ENSMUSG00000087236	Kif28
0.71937	-2.64149	1.83346467998601E-08	5.10103144179534E-08	ENSMUSG00000022422	Dscc1
0.85282	-2.69158	1.93249534379026E-08	5.36600701214594E-08	ENSMUSG00000020676	Ccl11
0.81743	-2.70500	2.1663242971257E-08	5.99080533282945E-08	ENSMUSG00000062609	Kcnj15
0.90227	0.70662	2.21151112590608E-08	6.10880504177114E-08	ENSMUSG00000079012	Serpina3m
0.72799	-2.81839	2.37892664795225E-08	6.55632801677691E-08	ENSMUSG00000046070	Igfals
0.82406	-3.06198	2.61073932967784E-08	7.16729795290354E-08	ENSMUSG00000054892	Txk
1.15822	-2.76020	2.67293191873999E-08	7.32619674662067E-08	ENSMUSG00000114865	Gm29776
0.77183	-2.65681	2.874182906187E-08	7.854990220448E-08	ENSMUSG00000045004	Spata21
1.12125	-3.57063	2.90641519626634E-08	7.93924768518615E-08	ENSMUSG00000032094	Cd3d
1.14594	-3.23348	3.11543356879909E-08	8.48564782215151E-08	ENSMUSG00000004612	Nkg7
0.98456	-3.08937	3.40765461209634E-08	9.24010160419675E-08	ENSMUSG00000026398	Nr5a2
1.29456	-3.24275	3.59548605202415E-08	9.72613328850101E-08	ENSMUSG00000066861	Oas1g
1.33761	-3.67619	3.77042734097224E-08	1.0181532295094E-07	ENSMUSG00000047171	Helt
0.78088	-3.19713	3.78432663804395E-08	1.02158177224044E-07	ENSMUSG00000094819	Olfr53
1.44732	-3.37416	3.78756430782117E-08	1.02229333430674E-07	ENSMUSG00000090942	F830016B08Rik
0.90887	-3.07288	3.87367394435118E-08	1.04404209053844E-07	ENSMUSG00000024786	Majin
1.43963	-3.05692	4.68185198752424E-08	1.24976903777596E-07	ENSMUSG00000028946	Hes3
0.90071	-3.21540	5.0139476856376E-08	1.33527109709377E-07	ENSMUSG00000054641	Mmrn1
0.70406	-2.75716	5.56992570880316E-08	1.47768064553167E-07	ENSMUSG00000074203	G430095P16Rik
0.85235	4.61814	5.65412341631592E-08	1.49870882503316E-07	ENSMUSG00000005268	Prlr
0.90820	-3.33382	5.90434830126272E-08	1.56332713438047E-07	ENSMUSG00000078127	Fam170b
0.78249	-2.26425	6.24697771292844E-08	1.65044568253854E-07	ENSMUSG00000039783	Kmo
1.25318	-2.19115	6.40723284317061E-08	1.69094409724689E-07	ENSMUSG00000033825	Tpsb2
1.15557	-3.20399	6.53728423263009E-08	1.72365954592457E-07	ENSMUSG00000035557	Krt17
1.30247	-1.63634	6.71363209739646E-08	1.76823538093909E-07	ENSMUSG00000061068	Mcpt4
0.87994	-1.95657	6.72351086665815E-08	1.77056274380389E-07	ENSMUSG00000042096	Dao
1.43733	-3.56304	6.89727046737113E-08	1.8149137345059E-07	ENSMUSG00000022596	Slurp1
-1.34937	-3.73176	7.65638987283163E-08	2.00751275134533E-07	ENSMUSG00000061259	Tmprss11d
1.48049	-3.09163	8.3614698347794E-08	2.1846301069399E-07	ENSMUSG00000006587	Snai3
0.93931	-3.04790	8.58158823859581E-08	2.23938588321453E-07	ENSMUSG00000021804	Rgr
0.99219	-3.48793	8.65794879655659E-08	2.25757842142599E-07	ENSMUSG00000063522	Ly6m
0.75421	0.63230	9.03426482150752E-08	2.35065233248231E-07	ENSMUSG00000049928	Glp2r
0.72111	-2.74111	9.43497550302363E-08	2.44928734482527E-07	ENSMUSG00000034224	Slc38a8
1.53454	-3.63076	1.00152932072385E-07	2.59359452750866E-07	ENSMUSG00000037974	Muc5ac
0.95686	-3.33595	1.00763894430497E-07	2.60862090610375E-07	ENSMUSG00000001027	Scn4a
1.25003	0.71096	1.0952718311026E-07	2.82644354652453E-07	ENSMUSG00000028749	Pla2g2f
0.70870	-2.69841	1.21941111539865E-07	3.13109976245348E-07	ENSMUSG00000091530	Cldn20
1.58469	-3.80747	1.27646367346829E-07	3.27215404932537E-07	ENSMUSG00000026573	Xcl1
0.92929	-3.34696	1.30242786343386E-07	3.33518910823251E-07	ENSMUSG00000022229	Atp12a
0.76309	-1.17366	1.54831388873242E-07	3.93930752460482E-07	ENSMUSG00000066363	Serpina3f

0.95415	-2.75777	1.6187442388479E-07	4.11295649559498E-07	ENSMUSG00000090675	Olfr111
1.21401	-3.65139	1.65502395029538E-07	4.20011157269465E-07	ENSMUSG00000051860	Samd7
1.21189	-3.17757	1.68220647084947E-07	4.26590887099429E-07	ENSMUSG00000030364	Clec2h
1.18696	-3.10264	1.8231707446633E-07	4.6055055182662E-07	ENSMUSG00000016458	Wt1
-1.01757	-3.48643	1.83578236491504E-07	4.63529589999652E-07	ENSMUSG00000060317	Acnat2
1.18182	-3.51241	1.8616787289587E-07	4.69579780958431E-07	ENSMUSG00000009185	Ccl8
0.99854	-3.54191	1.89013606053841E-07	4.76404026653212E-07	ENSMUSG00000043681	Fam25c
0.78548	-3.25283	1.92564489759911E-07	4.85066066435553E-07	ENSMUSG00000022657	Cd96
1.13343	-3.65600	2.01019475734263E-07	5.05464602393969E-07	ENSMUSG00000079001	4930404H24Rik
1.02520	-3.56310	2.02828699077231E-07	5.09712121290534E-07	ENSMUSG00000023987	Pgc
1.03167	0.57638	2.3161581871438E-07	5.78460677494838E-07	ENSMUSG00000044827	Tlr1
0.73594	-2.88452	2.533472041315E-07	6.30048646433306E-07	ENSMUSG00000019489	Cd70
0.74044	-2.69701	2.54334347583623E-07	6.32410991913143E-07	ENSMUSG00000058925	Ccdc192
0.93036	-3.25353	2.70889632862505E-07	6.72690115928699E-07	ENSMUSG00000074665	Bpifb4
-0.75827	-2.39642	2.71354163951508E-07	6.73548295910027E-07	ENSMUSG00000006574	Slc4a1
0.78740	-3.12489	2.73022931649491E-07	6.77195731181421E-07	ENSMUSG00000050108	Bpifc
0.82861	-2.73906	2.89532264622037E-07	7.16471101441975E-07	ENSMUSG00000032661	Oas3
-0.86761	2.31690	3.17262845809251E-07	7.82471141783908E-07	ENSMUSG00000038793	Lefty1
0.83127	-2.89991	3.20618412543183E-07	7.90632253198373E-07	ENSMUSG00000090667	Gm765
1.29029	-3.61329	3.33207868394983E-07	8.20724266825283E-07	ENSMUSG00000044359	P2ry4
0.87271	-3.35012	4.62267295408042E-07	1.12507117684696E-06	ENSMUSG00000078938	Syce3
0.80642	-3.22100	4.85412924742103E-07	1.17988192381154E-06	ENSMUSG00000045362	Tnfrsf26
1.21376	-3.21751	5.90959775749423E-07	1.42683693439898E-06	ENSMUSG00000048528	Nkx1-2
1.03024	-3.43779	6.15408336708517E-07	1.48460051462713E-06	ENSMUSG00000079190	AC133103.1
1.24575	-2.81088	6.16819144549638E-07	1.48779264909971E-06	ENSMUSG00000037161	Mgarp
0.85726	-3.37708	6.58602895970172E-07	1.58497605847022E-06	ENSMUSG00000024155	Meiob
0.72958	-2.93557	6.79170793111725E-07	1.63308611937466E-06	ENSMUSG00000050097	Ces2b
1.11723	-3.71625	6.86339537098666E-07	1.64985652415907E-06	ENSMUSG00000035528	Npffr2
0.70975	-1.83596	7.01357351461439E-07	1.68405069775646E-06	ENSMUSG00000006269	Atp6v1b1
1.02126	-2.68185	7.5613232989804E-07	1.80663516460027E-06	ENSMUSG00000074625	Arhgap40
1.00585	-3.06309	8.43561471786262E-07	2.00874996954093E-06	ENSMUSG00000044083	Efcab8
0.75533	-2.74527	9.19247347653871E-07	2.18224901368697E-06	ENSMUSG00000075510	Fam187a
1.13124	-3.69870	9.65460954242496E-07	2.28875951586262E-06	ENSMUSG00000028071	Sh2d2a
1.05532	-3.69486	9.66316199014089E-07	2.29046736275308E-06	ENSMUSG00000069607	Cd300ld3
0.97330	-3.53312	9.80494742891924E-07	2.32245464197546E-06	ENSMUSG00000070368	Prok1
1.00169	-3.46235	9.85046972244429E-07	2.33258683642157E-06	ENSMUSG00000013415	Igf2bp1
0.76863	-3.15854	9.93690134829108E-07	2.35239799477103E-06	ENSMUSG00000048329	Mfsd6l
0.91292	-3.03312	1.03006037728531E-06	2.43578308593025E-06	ENSMUSG00000026295	Spp2
0.77476	-3.13115	1.05006932932303E-06	2.48033617443544E-06	ENSMUSG00000039032	Tsga13
0.70218	-3.11894	1.1604353379607E-06	2.73077649553627E-06	ENSMUSG00000070354	Evi2
0.93531	-3.34851	1.32556723415353E-06	3.09919297740161E-06	ENSMUSG00000053522	Lgals7
0.85183	-2.95069	1.42733344950254E-06	3.33024868014684E-06	ENSMUSG00000026536	Ifi211
1.20750	-3.67463	1.43806479811903E-06	3.35344472072012E-06	ENSMUSG00000030156	Cd69
0.91276	-3.15660	1.47250325920765E-06	3.43045602954191E-06	ENSMUSG00000038179	Slamf7
0.98985	-3.41254	1.53108716239735E-06	3.56449345139184E-06	ENSMUSG00000043429	Ccdc185
0.73671	-2.86583	1.62643418377533E-06	3.77560315850987E-06	ENSMUSG00000048498	Cd300e
1.05785	-2.20047	1.68129353917525E-06	3.897627544147E-06	ENSMUSG00000023439	Gnb3
1.20634	-3.76926	1.75385759363559E-06	4.05698976044137E-06	ENSMUSG00000018211	Wfdc15b
0.70614	-2.73651	1.76017756338029E-06	4.06994643347003E-06	ENSMUSG00000030336	Cd27
0.73011	-2.97838	1.95609133904952E-06	4.50211078008038E-06	ENSMUSG00000112129	Pbld1
1.38136	-3.68498	2.06767964374945E-06	4.75185900811899E-06	ENSMUSG00000072778	Vmn2r27

0.98151	-3.43069	2.14469056651687E-06	4.92085404429884E-06	ENSMUSG00000030149	Klrk1
1.20859	-3.65710	2.1674121689228E-06	4.97097312348597E-06	ENSMUSG00000053044	Cd8b1
0.83387	-3.49273	2.20274672092558E-06	5.04792409214674E-06	ENSMUSG00000038567	Cyp24a1
1.02786	-3.72848	2.30947687177834E-06	5.27969224838789E-06	ENSMUSG00000028435	Aqp3
0.70241	-2.74495	2.34781399655405E-06	5.36300446059703E-06	ENSMUSG00000027460	Angpt4
1.20658	-3.65096	2.38449505972062E-06	5.44313384500589E-06	ENSMUSG00000023914	Mep1a
0.72862	0.75141	2.51186079963022E-06	5.71697364203084E-06	ENSMUSG00000040987	Mill2
1.10567	-1.77581	2.60051360736586E-06	5.90924627634179E-06	ENSMUSG00000064057	Scgb3a1
0.70537	-3.44661	2.63398481928695E-06	5.98210349064796E-06	ENSMUSG00000001076	C1ql4
0.91734	-3.39979	2.84860813600188E-06	6.44198016698615E-06	ENSMUSG00000057762	Gm6169
1.08139	-3.42287	3.17985598099142E-06	7.15393900212982E-06	ENSMUSG00000055546	Timd4
1.05019	-3.36170	3.40128005673768E-06	7.62482787065976E-06	ENSMUSG00000059434	Gckr
0.98953	-3.61110	3.43881782120242E-06	7.70796109600035E-06	ENSMUSG00000071552	Tigit
1.09886	-3.66603	3.54844307655103E-06	7.94416083576416E-06	ENSMUSG00000070280	Slc22a14
1.09184	-1.76258	3.64971761385806E-06	8.15164381070481E-06	ENSMUSG00000001865	Cpa3
0.97157	-3.47473	3.74917251140062E-06	8.36717585702493E-06	ENSMUSG00000079554	Aox2
0.75996	-3.11291	3.79565567660875E-06	8.46535363453189E-06	ENSMUSG00000060187	Lrrc10
1.02133	-3.59900	3.9969155738383E-06	8.89320258917682E-06	ENSMUSG00000032318	Isl2
0.80495	-3.15499	4.12429560750623E-06	9.1658207457242E-06	ENSMUSG00000090891	D6Ertd527e
0.96779	-0.16354	4.48291535043949E-06	9.93552067492055E-06	ENSMUSG00000057596	Trim30d
0.71404	-3.00714	4.83145158481554E-06	1.06717851414441E-05	ENSMUSG00000113880	A030005L19Rik
1.15381	-3.60728	5.4822810844868E-06	1.20498112386158E-05	ENSMUSG00000063851	Rnf183
1.14408	-3.61499	6.71585691227988E-06	1.46418091922242E-05	ENSMUSG00000031621	Isx
0.89824	-3.61459	6.82494513652291E-06	1.48605701075687E-05	ENSMUSG00000024837	Dmrt1
-0.70745	-2.99671	7.05989685155688E-06	1.53485446268554E-05	ENSMUSG00000004939	Nmrk2
0.70695	-1.89826	7.80240892055139E-06	1.6895770904057E-05	ENSMUSG00000053368	Rxfp2
0.77863	-2.96622	7.81912580071806E-06	1.69256822366549E-05	ENSMUSG00000024903	Lao1
0.93151	-3.40790	9.04513638179053E-06	1.94603314151289E-05	ENSMUSG00000057346	Apol9a
1.06570	-3.66387	1.00161070588393E-05	2.14488373522706E-05	ENSMUSG00000031966	Glb1l3
0.75738	2.38028	1.00291385187932E-05	2.14740364453314E-05	ENSMUSG00000030703	Gdpd3
0.70174	-2.86358	1.00848829642052E-05	2.15852326818593E-05	ENSMUSG00000028707	Dmbx1
0.87343	-3.34687	1.51742824797787E-05	3.19510053007536E-05	ENSMUSG00000066682	Pilrb2
1.00156	-3.01691	1.63250413009794E-05	3.42636275785815E-05	ENSMUSG00000031125	3830403N18Rik
1.01451	-3.63164	1.67100334545241E-05	3.50413588847618E-05	ENSMUSG00000034677	Gpr142
1.60783	-3.61079	1.76267089240987E-05	3.68862442969436E-05	ENSMUSG00000049719	Prss46
0.88718	-3.50229	1.81585426097876E-05	3.79617673954063E-05	ENSMUSG00000050578	Mmp13
-0.71927	-3.29492	2.0908611564634E-05	4.35022037060628E-05	ENSMUSG00000051839	Gypa
0.70116	-2.89979	2.39495175974563E-05	4.95982451475787E-05	ENSMUSG00000034641	Cd300ld
0.97908	-3.63390	2.4226026707957E-05	5.01342114404645E-05	ENSMUSG00000056632	Dsg3
1.18226	-3.43814	2.5021320487092E-05	5.16981503812598E-05	ENSMUSG00000026468	Lhx4
0.75058	-1.89574	2.72977602893205E-05	5.62102244599972E-05	ENSMUSG00000099974	Bcl2a1d
0.84802	-3.09980	3.08077053077791E-05	6.31544947228854E-05	ENSMUSG00000027606	Dnajc5b
1.05656	-3.69569	3.46109290555156E-05	7.06440541625734E-05	ENSMUSG00000047822	Angptl8
0.74018	-2.48329	3.61912095218369E-05	7.3745504061049E-05	ENSMUSG00000116376	Tmem249
1.02795	-3.68151	4.59504581815826E-05	9.26096077338624E-05	ENSMUSG00000073492	Gm10521
0.79591	-3.60686	4.65652414519919E-05	9.378189944119E-05	ENSMUSG00000028575	Eqtn
0.89950	-3.51549	5.15953192041763E-05	0.000103495251226892	ENSMUSG00000057534	Elobl
0.73499	-3.35211	5.19222820624417E-05	0.000104138810823703	ENSMUSG00000027713	1810062G17Rik
0.70826	-3.33910	5.29211878311109E-05	0.000106004614889154	ENSMUSG00000038071	Npy6r
0.77625	0.12463	5.74647652268415E-05	0.000114592256329802	ENSMUSG00000012428	Steap4
0.92411	-3.59380	6.00782127956122E-05	0.000119607251725285	ENSMUSG00000074417	Gm14548

0.88275	-3.12528	6.17244913584921E-05	0.000122729884072656	ENSMUSG00000023978	Prph2
1.42988	-3.24654	6.73698364061344E-05	0.000133389135299232	ENSMUSG00000041578	Crx
0.72444	-3.26252	7.0731771134539E-05	0.000139866293566936	ENSMUSG00000030996	Art1
0.83476	-3.68787	7.5082255021823E-05	0.000148124183986846	ENSMUSG00000066108	Muc5b
0.95967	-3.56801	8.72495874358356E-05	0.000170917434428041	ENSMUSG00000054545	Ugt1a6a
1.29219	-2.53216	9.57399809934054E-05	0.000186731434800915	ENSMUSG00000079492	Gm11127
0.88279	-3.56512	0.000105734527900189	0.000205375961578826	ENSMUSG00000004359	Spic
0.78629	-2.83452	0.000106464346430122	0.000206722633118633	ENSMUSG00000034159	Mab21l4
1.00368	-3.62756	0.000108143207035201	0.000209766704854304	ENSMUSG00000034634	Ly6d
0.95809	-3.66606	0.000109949856282795	0.000213032715222229	ENSMUSG00000095609	Gm21188
0.75069	-3.52959	0.000114729004937928	0.000221756324483504	ENSMUSG00000021211	Akr1c12
0.87083	-2.80504	0.000123331345112334	0.000237707384929467	ENSMUSG00000050014	Apol10b
0.81355	-3.60270	0.00012682629796611	0.000244138827178275	ENSMUSG00000079103	Tgm7
1.48507	-3.37923	0.000128708921090378	0.000247650600462491	ENSMUSG00000005649	Cabp5
1.02793	-3.76168	0.0001346276808078	0.000258541153240211	ENSMUSG00000043931	Gimap7
0.87501	-3.72463	0.000138344874564015	0.000265259901703555	ENSMUSG00000060459	Kng2
0.71290	-2.83296	0.000141818185438293	0.000271613002731197	ENSMUSG00000031766	Slc12a3
1.05512	-3.74105	0.000164378408927308	0.000312600516159981	ENSMUSG00000070547	Mrgprb1
1.20876	-3.39903	0.000173334163195071	0.000328785257297662	ENSMUSG00000019913	Sim1
0.70073	-3.48192	0.000187990066511595	0.000355434084574176	ENSMUSG00000022878	Adipoq
0.78690	-2.65617	0.000194786232123474	0.0003675064983689	ENSMUSG00000022595	Lypd2
0.82643	-3.53955	0.000200811455035529	0.000378160403297148	ENSMUSG00000086277	4930558K02Rik
0.95098	-3.70842	0.000203873078294749	0.000383713256599955	ENSMUSG00000073998	Olfr520
0.88595	-3.65616	0.000232335424371166	0.000434730057188827	ENSMUSG00000031450	Grk1
0.72626	-3.51519	0.000242722315790281	0.000453216827945185	ENSMUSG00000118454	AC150683.1
0.94406	-3.79062	0.000257727018272459	0.00047986262455201	ENSMUSG00000054938	Olfr1346
0.75288	-3.56920	0.000302436282826959	0.000558698083151847	ENSMUSG00000062154	Tex33
1.08192	-3.73418	0.000305635355614184	0.000564300991324177	ENSMUSG00000019893	Ros1
0.74085	-3.63746	0.000327368546643259	0.000601746036612821	ENSMUSG00000108622	Gm36864
0.70974	-3.53155	0.000354971726214944	0.000649322675238448	ENSMUSG00000026592	Tex35
0.85729	-3.67454	0.000401439826468826	0.000729999975599702	ENSMUSG00000040165	Cd209c
-0.78870	-3.61158	0.000406580861131401	0.000739111361038014	ENSMUSG00000118193	Arhgap26
1.01909	-3.73381	0.000408055233658637	0.000741712209436435	ENSMUSG00000037034	Pax1
0.73460	-3.42609	0.000431460806705372	0.000782080258675935	ENSMUSG00000052955	Cpvl
1.07875	-3.78449	0.000462052437299401	0.000835036894132152	ENSMUSG00000109156	Gm45194
0.79980	-3.64737	0.000543503213392384	0.000972000483115046	ENSMUSG00000044701	Il27
1.06796	-3.77652	0.000554321545376816	0.000990513770154764	ENSMUSG00000079460	4933403O08Rik
0.77280	-3.52619	0.000627421954240195	0.00111375592044226	ENSMUSG00000074677	Sirpb1c
0.80027	-3.60010	0.000677132427148834	0.00119587542077185	ENSMUSG00000018924	Alox15
0.84253	-3.56598	0.000683927298430174	0.00120724822794387	ENSMUSG00000043972	Opn5
0.88412	-3.55040	0.000774774034032701	0.00136124328163693	ENSMUSG00000027416	Otor
0.74360	-3.49662	0.000786043165843608	0.00137950657880286	ENSMUSG00000023013	Aqp2
0.70252	-3.50026	0.000814246509236289	0.00142612451147797	ENSMUSG00000067614	Krt86
0.89765	-3.60147	0.000842256583049574	0.00147355868515408	ENSMUSG00000027443	Cst12
-0.87149	-3.19634	0.0008501604404446	0.00148631527861201	ENSMUSG00000026180	Cxcr2
0.78756	2.37830	0.000858127266182741	0.00149978045246012	ENSMUSG00000075296	Aldh3b2
0.99919	-3.63842	0.000877956858715157	0.00153270281737263	ENSMUSG00000049699	Ucn2
0.70082	-3.59363	0.000938277427102181	0.00163231988681088	ENSMUSG00000094338	Hist1h2bl
-0.74858	-3.64415	0.00100404149365931	0.00174045731001493	ENSMUSG00000032098	Treh
-0.73435	-3.75483	0.00105308683154557	0.00181973747271856	ENSMUSG00000066154	Mup3
-1.05159	-3.40678	0.00106270481582787	0.00183542389297314	ENSMUSG00000022651	Retnlg

0.75053	-2.98568	0.00118569872046802	0.00203708028552899	ENSMUSG00000047592	Nxpe5
0.82636	-3.46484	0.00142678754782243	0.00242965786511702	ENSMUSG00000057000	Nxf3
0.85507	-2.95441	0.0014344437084622	0.00244171640474508	ENSMUSG00000062345	Serpinb2
0.73943	-3.71313	0.00148434698122112	0.00252160865169843	ENSMUSG00000070719	Pla2g4d
0.81904	-3.75029	0.00149461876231935	0.00253728225584902	ENSMUSG00000055891	Ubl4b
0.76174	-3.63925	0.00157205301460817	0.00266155437633681	ENSMUSG00000022181	C6
0.88108	-3.62082	0.00162264463488058	0.00274283453306977	ENSMUSG00000091618	H60c
0.82958	-3.51689	0.00230502896616012	0.00381766911154608	ENSMUSG00000043383	Olfr1342
0.75727	-1.41752	0.00255907242846757	0.00421377555634459	ENSMUSG00000002324	Rec8
-0.88778	-3.69111	0.00256617516810789	0.00422465197749945	ENSMUSG00000090643	Gm3453
0.72311	-3.54625	0.00292749298388911	0.00478977663587674	ENSMUSG00000022598	Psca
0.74550	-0.84220	0.00337135131676522	0.00547433723659028	ENSMUSG00000060441	Trim5
0.72244	-3.64377	0.00381157200979523	0.00614335723931701	ENSMUSG00000047034	Ankrd33
0.71593	-3.60873	0.00418750709482804	0.00671360612749517	ENSMUSG00000068614	Actc1
-0.78986	1.12272	0.00461417480160698	0.0073556822592281	ENSMUSG00000026822	Lcn2
-0.73555	-3.76560	0.00552335959869047	0.00871466823279964	ENSMUSG00000027022	Xirp2
0.93453	-3.29187	0.00789929286402804	0.0122049283468948	ENSMUSG00000022057	Adamdec1
0.75145	-3.07897	0.0100050523892476	0.0152517794318502	ENSMUSG00000078597	Cyp4a12b
0.73449	-3.39440	0.0100444635873523	0.0153091106605007	ENSMUSG00000041476	Smpx
-1.15489	-3.40045	0.010340226438656	0.0157387121888619	ENSMUSG00000096278	Dcpp2
0.70821	-3.74521	0.0167579688932783	0.0246864703051519	ENSMUSG00000070777	Ceacam20
0.81394	-3.75759	0.0231811387506532	0.0335149944762636	ENSMUSG00000058207	Serpina3k

Supplementary Table S3 (CD4 T- Microglia PICs DEG)

genes	log2f_real_vs_exp_PIC	Mic_t_ratio	qvals
KLRB1	0.814553734090823	-2.97251213321409	0
PTPRCAP	0.61099194136095	-2.17443648589007	0
ITM2A	0.649708358619824	-1.6955826156926	0
CST7	1.04152216293486	-1.50232391724726	0
CD96	0.825313953639464	-1.3931983554641	1.93686820967729E-11
GZMA	1.04396743585858	-1.2883700165372	0
CD247	0.883454994333637	-1.18945974033493	0
IQGAP2	0.598377076277634	-1.21106289483415	0
RAC2	0.695382409585882	-1.09929602242735	0
IFNG	1.87978299373988	-1.01526534430367	0
LSP1	0.940138041814957	-0.92009749207543	0
RARRES3	0.781302313627352	-0.926085093385659	0
PLP2	0.760626971557072	-0.856129013319949	2.53711668391911E-10
ODF2L	0.627100176293431	-0.713944802736491	1.27443096125515E-12
CUTA	0.795177797498008	-0.639034322500581	0
GNLY	1.66050483249666	-0.571471069226213	0
C9orf78	0.619972452516299	-0.552388543300176	0
PDCL3	0.931122361222554	-0.511712248466743	0
XCL2	2.6691734941943	-0.473263141519841	0
CDC42EP3	0.645533539528745	-0.454447242711806	1.62524314993738E-15
HIST1H1D	1.19779832378597	-0.472307986977753	0
XCL1	2.44350811227025	-0.383803924495217	0
EPB41L4A-AS1	0.915765014010922	-0.292232224298389	0
GNL1	0.664392150394668	-0.332376081193413	4.12262075437046E-09
NDUFA1	0.71565501918537	-0.330582873171333	0
ALDOA	0.734947194327303	-0.351629606128032	8.98694452189375E-14
PET100	0.735670600298502	-0.329330035559153	3.18414984477528E-15
SNRPE	0.707894734518137	-0.286558832064376	0
COX7A2L	0.862465180676181	-0.248429224383049	0
EAPP	0.737356575018301	-0.228335582870034	0
CALM3	0.790107484569866	-0.239730620131794	0
POLR3GL	1.46006323480818	-0.196583490930427	0

**MRPL47**	0.675049708982723	-0.206465357851031	0
**FRG1**	0.613563190622683	-0.178655369061515	2.41475433098211E-13
**OGT**	0.673104378416998	-0.216086847548842	1.30351525251559E-13
**FAM214A**	0.781405775090994	-0.190764845205855	0
**TXNIP**	0.734655916714163	-0.118185396645108	0
**CCL20**	1.50026918249255	-0.112489798337789	0
**H1FX**	0.769026144719443	-0.13793907890787	4.59221822509339E-13
**DDX50**	0.763111477879005	-0.114683523648469	3.10972086933078E-11
**ANXA11**	0.660373067180917	-0.116412913848554	1.33222348971716E-09
**GTF2A2**	0.83418355766972	-0.105450416458828	2.42105186473431E-15
**MRPS21**	0.86427217742503	-0.0795461630323665	0
**SRP9**	0.928942276146566	-0.0632017742432335	0
**GRPEL1**	0.717566742339502	-0.0702742439393717	5.72877428555272E-10
**LPXN**	1.14452822070712	-0.0655370749325449	0
**ARCN1**	0.989119339502035	-0.0661811422240258	0
**PEBP1**	0.678511310637413	-0.0642456678072975	0
**IFI27L2**	1.1402445800802	-0.0530666424386771	0
**RCOR1**	1.07647014363083	-0.0809445740304879	0
**PTPRA**	0.629961512232439	-0.0823486220037807	1.33503398636845E-12
**FKBP8**	0.918530303745502	-0.0602784491614391	0
**LGALS1**	0.802750101409788	-0.0419905075898078	0
**S100A9**	3.64512516265583	0.00226991834386125	0
**SMARCC1**	1.12787236660398	0.0171032172519765	0
**TMEM167A**	0.757723801713443	-0.0181449725722588	3.58506612554549E-11
**UBE3A**	0.70473496860599	0.0160153313357291	3.38907813315167E-09
**NARF**	0.798741273762905	-0.0191312685090735	0
**ADRM1**	0.709837183935533	-0.00020704865072207	8.28557437367555E-11
**BAZ1B**	0.591806300938485	0.0947294890647806	3.09871967350317E-08
**RSBN1L**	0.597389038908567	0.0888935855544417	8.31811192883614E-14
**RIC8A**	1.22516984609163	0.0845611264399196	0
**TBC1D4**	0.643725474262412	0.0723055827071376	1.06370470562385E-09
**STXBP3**	0.610815236123061	0.145633857358259	7.48801807161561E-07
**AHCTF1**	0.808085195696259	0.120477552595074	6.04243220601906E-08
**ZNF721**	0.674075901438571	0.146392698923302	4.03982869149061E-07

**SRI**	0.823683261954932	0.129980142148358	1.04511860954122E-09
**CFDP1**	0.596813620149649	0.128606565385968	1.3247616442156E-10
**EVI2A**	0.681024743520296	0.122111467397797	0
**ZNF791**	0.72070657688729	0.106546330018481	0
**RBX1**	0.605647622835412	0.134295857834846	2.03528504037295E-13
**YIPF4**	0.647716039385326	0.193786511247105	7.10455422263091E-09
**RAP2B**	0.616098376600999	0.181184700433913	4.67718561172156E-12
**MLEC**	0.590076332343555	0.166884650769718	1.47655217213196E-10
**DYNC1LI2**	0.782678079491949	0.221531259087289	2.96246852646814E-14
**PBRM1**	0.691572435856093	0.259819912864145	0
**IPO7**	0.622226960412176	0.232933001044647	1.83125514752192E-10
**GOSR1**	0.721189375240589	0.263372310241443	1.45050272159686E-12
**ESF1**	0.84564450863313	0.264085248548255	0
**REV3L**	0.793049202598873	0.289884712571308	0
**TRIP11**	0.791733282683808	0.331120228478137	0
**PGP**	0.703053760866298	0.309655203191577	1.97908698160991E-10
**TRIP12**	0.73102832031591	0.353514539362791	1.72345870630419E-09
**SHPRH**	0.87300145227598	0.375008987546178	0
**SMS**	0.75174110579345	0.396442454334449	0
**ITFG2**	0.87759364496442	0.391407395078733	0
**MT2A**	0.666287328572414	0.377328799820524	0
**DDX3Y**	1.48540396490485	0.382964280121462	0
**SESN1**	0.727500343584898	0.441365328477252	0
**SQLE**	1.15560803442541	0.467594149079139	0
**TSC22D1**	1.18097802167612	0.445342268578849	0
**NSRP1**	0.601479757048567	0.452118022320197	3.11078953230401E-11
**DRAM2**	0.745826973110064	0.48619787701926	0
**ERO1LB**	1.17380371591699	0.507982833488003	0
**ERICH1**	1.24104765232355	0.509545018573296	0
**ARL5B**	0.593203436287998	0.489482576518097	1.31442281041091E-11
**RBBP6**	0.755021565817818	0.526990374550597	0
**SLC16A3**	0.678231949767449	0.491710118120406	0
**COQ7**	0.597342196598683	0.550625684614853	0
**C3orf58**	0.629583179517998	0.618793673581724	0

**TPP1**	0.664984027060113	0.669312365822371	1.93217804095283E-09
**SETD8**	0.769375980970856	0.651385654767786	8.26005930321117E-16
**LDLRAD4**	0.717852884953753	0.657694129523278	0
**ANKRD37**	0.762680639959291	0.72645715159618	0
**GEM**	0.866849405203659	0.70740122946004	0
**IFIT2**	1.54869374471017	0.730874134932945	0
**SETDB2**	0.774154475478083	0.725751696033764	0
**BRI3**	0.66472615027259	0.796323094971056	3.78394194184561E-08
**IL1RN**	1.21852428480769	0.824809142724758	0
**PLIN2**	0.750459026333787	0.852935489599073	0
**KCNQ1OT1**	0.644919202245333	0.838299571519458	0
**C15orf48**	0.680754833630849	0.823846850921648	4.701596255176E-15
**CTD-2165H16.4**	0.892780106777193	0.915234344233235	0
**TMBIM4.1**	0.591653929591747	0.86957603382668	0
**SOX4**	0.819508484835402	0.966968629008108	0
**RRAD**	1.14498732158104	0.95223107303078	0
**MAFF**	0.689224942900114	0.947081518281469	0
**DBI**	0.72355243601894	1.02551227493342	0
**MARCH1**	0.817860276550325	1.05012596450491	4.62892261394555E-12
**HBEGF**	0.67230914936093	1.02559317772079	8.55358721583824E-09
**INHBA**	1.23295388139026	1.02898213990607	0
**GADD45G**	0.60087212569916	1.0215263834196	8.26005930321117E-16
**HES1**	0.631088147186793	1.11365986582901	0
**CITED2**	0.762262480400942	1.06873350081399	0
**DDIT4**	0.629907056824863	1.05635696078353	0
**SPRY2**	0.813198321129123	1.0597040525077	0
**FAM105A**	0.765782515101118	1.1314444924933	6.13104372414553E-10
**SPRY1**	0.689257739594931	1.20703610445598	0
**FAM46A**	1.04128877445386	1.30518546640371	0
**CEBPA**	0.593573092140312	1.26687956951655	0
**IL1A**	0.81382996081389	1.44457326135209	0
**RGS16**	0.702834955698722	1.54183108424342	0
**TREM2**	0.646801659905576	1.69286289186449	1.65618777951219E-11
**EIF4E**	0.7565353288414	1.79045562330179	0

**CCL2**	1.09340634889887	1.77246382083844	0
**CCL4L2**	0.599953891221256	1.78793688537434	0
**PTGS2**	0.655060759240041	1.89554484276299	0
**IFI30**	0.674824704507185	2.00408221524545	0
**USP53**	0.630318156862069	2.32748038209313	0
**G0S2**	1.0115312990123	2.62431275287941	0
**PDK4**	0.611911192560994	2.65464767871311	0
**CCL4**	0.872755195577162	3.05432996503944	0
**IL1B**	0.670107362013926	3.2009716651803	0

Supplementary Table S4 (CD8 T-Microglia PICs DEG)

genes	log2f_real_vs_exp_PIC	Mic_t_ratio	qvals
NKG7	0.597369448392332	-3.4288017412879	0
SYNE2	0.723626503400366	-2.12793039170235	2.79110068390764E-13
XCL1	0.621552561147123	-1.68883650186952	0
GZMB	0.950694934586858	-1.35217844920816	0
CCDC64	0.598560701223649	-1.23319883705043	4.07177702196427E-13
GIMAP7	0.644392001013238	-1.17637278348013	0
CD27	0.897250638608429	-1.17993256820457	0
HIST1H1D	0.683287251140331	-1.01001874588705	0
GPR65	0.621899707772318	-0.89897878225875	0
CMC1	0.613948960928901	-0.839338672813367	0
GNLY	2.28768801387775	-0.759130946703395	0
MXRA7	0.891602888833554	-0.669062196849943	0
RBM38	0.823253485465207	-0.676285233657935	0
CDC42EP3	0.617424730776478	-0.645933824963539	2.54565445028886E-14
CMC2	0.785789604043957	-0.466804830959572	0
STMN1	0.702141059599222	-0.425870854783617	0
UAP1	0.641738140345385	-0.39053997149338	0
HIST1H4C	0.684833846393878	-0.401873114766634	0
PRKACB	0.735986072968119	-0.348634041656402	0
LSM 5.00	0.757589488956716	-0.350549678339044	0
DDX3Y	1.05941136180081	-0.339583087650903	0
SF3A1	0.734597417412064	-0.315644537359831	0
SNRPE	0.750406497897706	-0.311278920752844	0
PPM1G	0.711362303582612	-0.293488220932431	0
PRKAR2A	0.694132919341625	-0.26194864065436	0
GLCCI1	0.686771064075411	-0.301268791144689	0
NDUFB6	0.609751398755887	-0.245371246521616	4.88572231804488E-09
MED17	0.76892485431204	-0.254260226320939	1.69843751702977E-14
SIRT7	0.972374900155906	-0.269166570909991	0
DCXR	0.681779449620292	-0.275512504980717	7.98215789854161E-12
B3GNT2	0.634306474948893	-0.207033068890472	3.88221073723427E-15
LSM 3.00	0.598875798235628	-0.231783067734093	1.71121130086395E-11

**GIMAP1**	0.613068038079404	-0.211421106647331	1.14480260842877E-11
**ANKRD49**	0.652406523569545	-0.174050226855136	0
**GTF2A2**	0.62902948270765	-0.207541013642924	0
**RMND5A**	0.830533568733594	-0.167350241464574	1.70136236254077E-12
**CRBN**	0.702741570944783	-0.128625335232297	0
**MSL2**	0.737738583790829	-0.139563616356949	0
**RBM4**	0.599189936148565	-0.105920515298482	1.80221047557205E-10
**UQCRC2**	0.610571540938872	-0.144516157667001	2.67230682027275E-11
**SLFN11**	1.26972738222419	-0.137005067728374	0
**RAB22A**	0.634943204190029	-0.104568716214604	0
**LDLR**	0.757054795914051	-0.137990867881212	0
**FAM126B**	0.774757545101352	-0.0779265995421168	0
**GPRIN3**	0.597858672438664	-0.068710933771985	0
**COPS6**	0.721395829748426	-0.0588847743837044	5.56423444985695E-14
**C7orf73**	0.824165655137383	-0.102495550388144	0
**BFAR**	0.813778146355496	-0.0897756026365406	0
**ENOSF1**	1.08312342669128	-0.07262744036079	0
**PTCD3**	0.895676554755575	-0.0106498394035294	0
**NUP153**	0.990281364970213	0.000149645181643438	0
**COPS5**	0.642976616096942	0.0125160944439525	7.13106503788853E-13
**ADIPOR2**	0.917239026447906	-0.0108319448784009	0
**SMARCD1**	0.708797301131384	0.0241930487969955	1.31213200570386E-13
**CCR7**	0.975356397293591	-0.0121113528791497	0
**TMEM50B**	0.69108148682747	0.0152594789777024	0
**RAB4A**	0.958487158629203	0.0754267604332185	0
**AHCTF1**	0.78568316608219	0.061224383442653	0
**IMMT**	0.638651027596524	0.0509624160004907	0
**MAT2B**	0.85274233434733	0.0661465710639069	0
**RIOK1**	0.738872535665802	0.0947383455123022	0
**LSM 2.00**	0.594933541183655	0.0398450336288056	1.26529606873147E-07
**ZNF292**	0.681921873347276	0.0519741485421612	0
**SUMO3**	0.602517729807388	0.0656264836970896	1.59651906020477E-15
**PPWD1**	0.769668193624152	0.133628443002308	0
**PDS5B**	1.11283639486819	0.110428275805389	0

**KLHL28**	0.683315796898374	0.164436429649053	2.1344231743353E-13
**SMURF2**	0.653764837027569	0.105293750122488	0
**TSEN34**	1.24370602198776	0.108816300267549	0
**HSD17B7**	0.594561389780519	0.175226409053876	4.00476555378619E-13
**PSMD1**	0.70020281737165	0.234879711685917	0
**ABHD5**	0.938922467855623	0.221660290975045	0
**TWF2**	0.717529041542572	0.22075561143118	0
**FAM13B**	0.646592294170839	0.224139207617176	1.38828238583157E-10
**CBX4**	0.872315019461327	0.210767387545051	0
**PSTPIP2**	0.90925008096442	0.22898031754718	0
**SMAD2**	0.707300338574396	0.222892087365823	8.11675656658728E-16
**TIA1**	0.665839064669346	0.273808545018676	6.70759744044365E-14
**HIST1H2AC**	0.921413090218405	0.302947663157084	0
**CKS2**	0.591248622597772	0.304621803422684	0
**CCNY**	0.687024349434393	0.246761352517198	0
**ZNF37A**	0.814824482607911	0.24957800899502	1.27272361690469E-13
**NRBF2**	0.671585215043241	0.285080555281214	0
**REEP3**	1.03797569653691	0.298417835196236	0
**SNAPC1**	0.64122431281504	0.260659147039545	0
**TLK2**	0.609881045810281	0.292545829986184	0
**CCL20**	1.49229462829366	0.345807569579432	0
**RLIM**	0.643751422233492	0.320763392606533	1.59651906020477E-15
**TCEAL8**	0.905146959059775	0.34781440537423	0
**SQLE**	0.880870841405957	0.375765468987974	0
**KLF9**	0.656704007680111	0.347764506055327	8.11675656658728E-16
**MARK3**	0.595820911880343	0.314932026462975	1.58909241437938E-12
**CMIP**	0.702136074764823	0.324806031584684	2.61052440925375E-14
**CYB5D2**	1.6820656103612	0.377590993304984	0
**NLRP1**	0.752974625256856	0.340436153236244	0
**NPC1**	0.855410066411069	0.355247415845838	0
**VPS13D**	0.653228894603193	0.423729361770344	2.08330085209073E-13
**AUP1**	0.754928321807729	0.387603284433196	0
**N4BP2**	0.619629406846273	0.383274040775202	5.94827232402751E-09
**ATRX**	0.678994125706839	0.384361706440067	1.60191179369004E-08

**SUSD3**	0.704249026713276	0.406655084782563	2.35967597253392E-15
**RAB14**	0.625707965994349	0.404668800303148	1.87473103145564E-13
**NSUN6**	0.668396010763518	0.413005347206798	0
**LMBR1L**	0.739428875076985	0.425912341200127	0
**RRAGC**	0.702122180682432	0.486181545822694	5.35757386685198E-15
**BEX4**	0.609638542437666	0.510026776413771	8.41781149889392E-13
**NFIL3**	0.643383866135193	0.463278489707524	0
**FAM111A**	0.725625680880144	0.465529861283132	0
**STAT6**	0.703558357662512	0.518456925806882	0
**TSC22D1**	1.2087806722632	0.48538811297735	0
**AIDA**	0.630003609255807	0.580728511036152	0
**METTL21A**	0.696796647059988	0.568931269577697	0
**ACSL4**	0.99289949642643	0.55706540634296	0
**KIAA2026**	0.668006894174564	0.530632734263875	4.43819079152351E-12
**PDE3B**	0.632024630666317	0.571384404739623	4.55004135396908E-08
**TRIP11**	0.677567122534255	0.545313310374202	1.82295493254649E-13
**RP5-821D11**	0.841143582855746	0.553425200594284	0
**VAMP3**	0.689254748512408	0.615989853032436	1.44690182094513E-10
**MYC**	0.882318051708019	0.607827722433812	0
**ERV3-1**	0.690617231276264	0.676810621092267	0
**CDKN1C**	0.729438395943623	0.678440045009623	0
**PRKD3**	0.926901789556815	0.73714700574339	0
**CXCL2**	1.42616821019774	0.738559169605706	0
**KCNQ1OT1**	1.10554324213994	0.764018273364772	0
**PPFIA1**	0.767926324945262	0.797572897226113	0
**CD163**	1.54574609775936	0.732028390177455	0
**ETV6**	0.852681247533714	0.768953989258265	0
**ATP2A2**	0.630488722897891	0.744787680613548	1.04501672642461E-14
**BTBD7**	0.682461371953946	0.77252548913383	1.12779890488606E-13
**SETDB2**	0.607180947207065	0.824878938235882	0
**HNRNPU-AS**	0.796897691590703	0.924167217117103	0
**INTS10**	0.653880701709834	0.87598419405083	6.82884264199103E-14
**SPRY1**	0.989644575567781	1.00763103745998	0
**SLC31A2**	0.654847048924286	0.953743664375171	0

**SLC8B1**	0.626514667444194	0.939027169132902	8.11675656658728E-16
**TLR7**	0.767573674230181	1.018147980757	0
**IGSF21**	0.785904696511156	1.11582710400295	0
**FILIP1L**	0.616901773063641	1.13960816091837	4.13200282522299E-08
**CTD-2165H1**	1.51362455449177	1.11303955279581	0
**GCNT2**	0.891855246070435	1.12015409401191	0
**SEPP1**	0.733541362667324	1.20755608701393	0
**SLC7A8**	0.886345434545531	1.17180417609548	0
**C1orf162**	0.614992276659378	1.26862276746477	1.15983840761468E-12
**RGS18**	0.79679584381698	1.24376956552464	0
**MS4A6A**	0.808647821430445	1.23971238184238	0
**HES1**	0.879830318730713	1.29163749877277	0
**HBEGF**	0.598698220157028	1.3609823863614	8.11675656658728E-16
**GRID2**	0.787539796720964	1.45137013283345	0
**CEBPA**	0.629462023333056	1.52866626134376	0
**SOX4**	0.609534614455726	1.61431669366628	0
**RGS16**	0.77745025724401	1.69490805789568	0
**BCO2**	0.639506784482159	1.63669667701949	1.16764013813045E-13
**RAB31**	0.60439316737485	1.68714012958791	0
**FGL2**	0.746598612021768	1.74427871176786	0
**HLA-DQA1**	0.738963871924594	1.93857423834904	0
**HLA-DQB1**	0.625642336903425	1.92766156913222	0
**PTGS2**	0.59066154706017	2.03725329825237	0
**EIF4E**	0.710062853011172	2.09737122794737	0
**CCL4**	0.683740033954102	2.20163219661916	0
**HLA-DRB5**	0.604880361401589	2.33463529049982	0
**SERPINE1**	0.626588713336283	2.72375246075658	0
**MAFB**	0.680930669827144	2.98747687433483	0
**G0S2**	0.730004226697268	3.18335412044046	0
**IL8**	0.870413674406803	3.4773947773314	0

Supplementary Table S5: Clinical information of the patients

Patient	Age (Gender)	Dx	Seizure onset	Seizure Semiology	Frequency	EEG	MRI/ Neuroimaging	Medication/ Surgery	Histopathology
1	9y (F)	R OLE	18m	Focal motor seizures	10 -20 per month	R occipital spike waves	Normal/ Ictal FDG PET: Hypermetabolism R occipital lobe and adjacent R infero-posterior parietal lobe.	CBZ, TPM, CLZ, PHT, PMP<br><br>R Occipital lobectomy	Chaslin subpial gliosis<br>Normal six-layered neuronal lamination without cortical thinning
2	4y (F)	FCD IIB	3y	Focal motor	> 30 per day	R fronto-centro - temporal spike waves	focal area of cortical thickening in the R frontal lobe gyrus	CLZ, OCZ, LVT, TPM, PHB<br><br>R frontal lesionectomy	Loss of 6-layer and columnar organization of the cortex<br>Presence of large, abnormally oriented dysplastic neurones with clumped Nissl substance.<br>Large balloon cells, with glassy eosinophilic cytoplasm, eccentric vesicular nuclei and distinct nucleoli are seen throughout all layers of the brain
3	18y (M)	FCD IIB	3m	Focal non-motor<br>Focal motor	1-2 per month	L fronto-centro - temporal spike waves	thickening of the L superior frontal gyrus with subcortical white matter signals extending to the L lateral ventricle	CBZ, VPA, CLZ<br><br>L frontal lesionectomy	Chaslin's subpial gliosis<br>Normal laminar configuration of cortical neurons is mildly disarrayed .<br>Dysmorphic, enlarged neurons with prominent CD34 and NeuN negative balloon neurons

4	3y (F)	TLE	10m	AS, Focal-motor	20-30 per day	L fronto temporal slow with spikes	abnormal FLAIR signal over L hippocampus and temporal tissue	LVT, TPM, OCZ, VPA<br><br>L temporal-lobectomy	Aggregates of oligodendroglial-like cells with evenly-sized, round nuclei, perinuclear clearing and thin, short dendritic processes, positive for GFAP, Olig2 and MAP2 and negative for CD34, NeuN and neurofilament stains<br>Poorly-oriented hippocampal tissue with intact granular cell layer<br>Focal areas with loss of pyramidal neurons with accompanying increased gliosis
5	22y (F)	SWS	3m	Focal non-motor	1 every 2 m	Attenuation over R posterior quadrant and sharp and spike waves over R temporal region	R cerebral atrophy with gyriform foci of susceptibility	CBZ, TPM, PHT<br><br>R- temporo-occipital lobectomy	Leptomeningeal hypervascularity c/w SWS.<br>Widespread intracortical psammomatous calcifications<br>Superficial cortical perivascular calcification<br>Astrogliosis in the neocortex<br>Neurofilament protein shows abnormal perikaryonal staining<br>A few dysplastic neurons
6	4y (F)	SRFSE	4y8 m	Focal motor	7-8 per hour	Rhythmic ictal activity over R posterior quadrant	Gyral restricted diffusion and T2 hyperintensity in R temporal lobe and R inferior parietal lobe	OCZ, LVT, TPM, PMP, THP, MDZ, KTM<br><br>R temporal lobectomy	Foci of perivascular and subpial inflammation<br>Diffusely widespread CD68 microglial cells with scattered foci forming small clusters<br>A few CD3-positive T-lymphocytes are present<br>GFAP shows reactive astrocytes, with some Chaslin gliosis in the hippocampal region

AS: absence seizures; CBZ: Carbamazepine, CLZ: Clobazam; c/w: consistent with; m: months; GFAP: glial fibrillary acidic protein; FCD: focal cortical dysplasia; LVT: Levetiracetam; MAP2: microtubule- associated protein 2; NeuN: neuronal nuclei; OCZ: Oxcarbazepine; OLE: Occipital lobe epilepsy; Olig2: oligodendrocyte transcription factor 2; PHB: Phenobarbitone; PHT: Phenytoin, PMP: Perampanel; R: right; SRFSE: Super-refractory focal status epilepticus; SWS: Sturge Weber Syndrome; TLE: temporal lobe epilepsy; TPM: Topiramate, VPA: Valproate; y: years
