# Supplementary material for: Single-cell transcriptomics and surface epitope detection in human brain epileptic lesions identifies pro-inflammatory signaling
Source: Nat Neurosci. 2022 Jun 23;25(7):956–66. doi: 10.1038/s41593-022-01095-5 (PMC9276529; doi:10.1038/s41593-022-01095-5)
Supplement: Supplementary file 2 — Reporting Summary [file 41593_2022_1095_MOESM2_ESM.pdf]

nature portfolio

Corresponding author(s): Salvatore Albani, Pavanish Kumar, Derrick Chan

Last updated by author(s): April 21, 2022

Reporting Summary

Nature Portfolio wishes to improve the reproducibility of the work that we publish. This form provides structure for consistency and transparency in reporting. For further information on Nature Portfolio policies, see our Editorial Policies and the Editorial Policy Checklist.

Statistics

For all statistical analyses, confirm that the following items are present in the figure legend, table legend, main text, or Methods section.

n/a	Confirmed	
☐	☒	The exact sample size (*n*) for each experimental group/condition, given as a discrete number and unit of measurement
☐	☒	A statement on whether measurements were taken from distinct samples or whether the same sample was measured repeatedly
☐	☒	The statistical test(s) used AND whether they are one- or two-sided<br>*Only common tests should be described solely by name; describe more complex techniques in the Methods section.*
☐	☒	A description of all covariates tested
☐	☒	A description of any assumptions or corrections, such as tests of normality and adjustment for multiple comparisons
☐	☒	A full description of the statistical parameters including central tendency (e.g. means) or other basic estimates (e.g. regression coefficient) AND variation (e.g. standard deviation) or associated estimates of uncertainty (e.g. confidence intervals)
☐	☒	For null hypothesis testing, the test statistic (e.g. *F*, *t*, *r*) with confidence intervals, effect sizes, degrees of freedom and *P* value noted<br>*Give P values as exact values whenever suitable.*
☒	☐	For Bayesian analysis, information on the choice of priors and Markov chain Monte Carlo settings
☐	☒	For hierarchical and complex designs, identification of the appropriate level for tests and full reporting of outcomes
☒	☐	Estimates of effect sizes (e.g. Cohen's *d*, Pearson's *r*), indicating how they were calculated

Our web collection on statistics for biologists contains articles on many of the points above.

Software and code

Policy information about availability of computer code

Data collection	Single cell CITE-Seq sequence data was collected using Illumina Hi-Seq platform. Raw sequence reads were obtained and analyzed.
Data analysis	For 10x single cell sequence analysis, 10x Genomics CellRanger v3 software were used. For clustering and downstream analysis R version 4.0.3 and R studio version 1.3 were used. Mouse reads were aligned to mouse genome (mm.GRCm38.97 version) using STAR2 (STAR_2.4) software. Following R packages were used : Seurat (v-3.2), edgeR (3.32), igraph(1.26), visNetwork(2.0.9), CellChat(1.1), CiteFuse(1.1), ggplot2(3.3.2)

For manuscripts utilizing custom algorithms or software that are central to the research but not yet described in published literature, software must be made available to editors and reviewers. We strongly encourage code deposition in a community repository (e.g. GitHub). See the Nature Portfolio guidelines for submitting code & software for further information.

Data

Policy information about availability of data

All manuscripts must include a data availability statement. This statement should provide the following information, where applicable:

- Accession codes, unique identifiers, or web links for publicly available datasets
- A description of any restrictions on data availability
- For clinical datasets or third party data, please ensure that the statement adheres to our policy

Raw counts data is deposited in GEO public repository (GEO accession number: GSE201048) and Raw counts and analyzed R object is also available at https://epicimmuneatlas.org/NatNeu2022

Masuda et al, data was accessed from GEO (accession number: GSE124335)

Velmeshev et al data was downloaded from https://autism.cells.ucsc.edu
Shrivastava Pk et al data accessed from GEO (accession number: GSE77578)

Field-specific reporting

Please select the one below that is the best fit for your research. If you are not sure, read the appropriate sections before making your selection.

☒ Life sciences ☐ Behavioural & social sciences ☐ Ecological, evolutionary & environmental sciences

For a reference copy of the document with all sections, see nature.com/documents/nr-reporting-summary-flat.pdf

Life sciences study design

All studies must disclose on these points even when the disclosure is negative.

Sample size	Its a proof of concept basic research study and no sample size calculation was performed.
Data exclusions	No data excluded
Replication	Multiple samples were analyzed to ensure reproducibility.
Randomization	study did not required randomization
Blinding	Blinding is not relevant for the study

Reporting for specific materials, systems and methods

We require information from authors about some types of materials, experimental systems and methods used in many studies. Here, indicate whether each material, system or method listed is relevant to your study. If you are not sure if a list item applies to your research, read the appropriate section before selecting a response.

Materials & experimental systems

n/a	Involved in the study	
☐	☒	Antibodies
☒	☐	Eukaryotic cell lines
☒	☐	Palaeontology and archaeology
☒	☐	Animals and other organisms
☐	☒	Human research participants
☒	☐	Clinical data
☐	☐	Dual use research of concern

Methods

n/a	Involved in the study	
☒	☐	ChIP-seq
☒	☐	Flow cytometry
☒	☐	MRI-based neuroimaging

Antibodies

Antibodies used

Anti-Iba1 antibody [EPR16588] : (Abcam)
Polyclonal Rabbit Anti-Human CD3 (Dako Omnis)
Monoclonal Mouse Anti-HumanCD68 (clone PG-M1, Dako Omnis)
Anti-MAP2 Antibody, clone AP20 (MAB 3418, Thermo Fisher Scientific)
IL-1 beta Antibody (P420B, Thermo Fisher Scientific)
GFAP Antibody, clone GA5 (14-9892-82, Thermo Fisher Scientific)

TotalSeq™-B Barcode 0034 Specificiy CD3 Clone UCHT11 10 µg
TotalSeq™-B Barcode 0050 Specificity CD19 Clone HIB19 10 µg
TotalSeq™-B Barcode 0072 Specificity CD4 Clone RPA-T4 10 µg
TotalSeq™-B Barcode 0080 Specificity CD8a Clone RPA-T8 10 µg
TotalSeq™-B Barcode 0081 Specificity CD14 Clone M5E2 10 µg
TotalSeq™-B Barcode 0083 Specificity CD16 Clone 3G8 10 µg
TotalSeq™-B Barcode 0084 Specificity CD56B (NCAM) Clone QA17A16 10 µg
TotalSeq™-B Barcode 0085 Specificity CD25 Clone BC96 10 µg
TotalSeq™-B Barcode 0100 Specificity CD20 Clone 2H7 10 µg
TotalSeq™-B Barcode 0101 Specificity CD335 (NKp46) Clone 9E2 10 µg
TotalSeq™-B Barcode 0146 Specificity CD69 Clone FN50 10 µg
TotalSeq™-B Barcode 0148 Specificity CD197 (CCR7) Clone G043H7 10 µg
TotalSeq™-B Barcode 0154 Specificity CD27 Clone O323 10 µg

TotalSeq™-B Barcode 0159 Specificity HLA-DR Clone L243 10 µg
TotalSeq™-B Barcode 0161 Specificity CD11b Clone ICRF44 10 µg
TotalSeq™-B Barcode 0391 Specificity CD45 Clone HI30 10 µg

Catalog Number
300477
302263
300565
301069
301857
302063
392423
302647
302361
331939
310949
353249
302851
307661
301357
304066

All antibodies were purchased from Biolegend

Validation

Antibodies were validated for research purpose use only

Human research participants

Policy information about studies involving human research participants

Population characteristics

Pediatric patients (n=6) with drug-refractory epilepsy and who had a focal lesion amenable to surgical resection were identified through detailed seizure semiology, neuroimaging, electroencephalography (EEG) monitoring studies and functional imaging (DC)

Recruitment

Pediatric patients with drug-refractory epilepsy and who had a focal lesion amenable to surgical resection were identified through detailed seizure semiology, neuroimaging, electroencephalography (EEG) monitoring studies and functional imaging (DC). The epileptogenic zone was identified in each patient and epilepsy surgery performed (DL) to resect the epileptic brain to achieve seizure control. Histopathological examination was performed to identify aetiologies such as neuronal migration disorders, cortical dysplasia etc

Ethics oversight

The study was reviewed and approved by the SingHealth Central Institutional Review Board. The Informed consent was obtained according to the SingHealth Central Institutional Review Board requirements.

Note that full information on the approval of the study protocol must also be provided in the manuscript.

Dual use research of concern

Policy information about dual use research of concern

Hazards

Could the accidental, deliberate or reckless misuse of agents or technologies generated in the work, or the application of information presented in the manuscript, pose a threat to:

No	Yes	
☒	☐	Public health
☒	☐	National security
☒	☐	Crops and/or livestock
☒	☐	Ecosystems
☒	☐	Any other significant area

Experiments of concern

Does the work involve any of these experiments of concern:

No	Yes	
☒	☐	Demonstrate how to render a vaccine ineffective
☒	☐	Confer resistance to therapeutically useful antibiotics or antiviral agents
☒	☐	Enhance the virulence of a pathogen or render a nonpathogen virulent
☒	☐	Increase transmissibility of a pathogen
☒	☐	Alter the host range of a pathogen
☒	☐	Enable evasion of diagnostic/detection modalities
☒	☐	Enable the weaponization of a biological agent or toxin
☒	☐	Any other potentially harmful combination of experiments and agents
